# A new ensemble coevolution system for detecting HIV-1 protein coevolution

**DOI:** 10.1186/s13062-014-0031-8

**Published:** 2015-01-07

**Authors:** Guangdi Li, Kristof Theys, Jens Verheyen, Andrea-Clemencia Pineda-Peña, Ricardo Khouri, Supinya Piampongsant, Mónica Eusébio, Jan Ramon, Anne-Mieke Vandamme

**Affiliations:** KU Leuven - University of Leuven, Department of Microbiology and Immunology, Rega Institute for Medical Research, Clinical and Epidemiological Virology, Leuven, Belgium; Institute of Virology, University hospital, University Duisburg-Essen, Essen, Germany; Clinical and Molecular Infectious Disease Group, Faculty of Sciences and Mathematics, Universidad del Rosario, Bogotá, Colombia; Centro de Malária e Outras Doenças Tropicais and Unidade de Microbiologia, Instituto de Higiene e Medicina Tropical, Universidade Nova de Lisboa, Lisboa, Portugal; Department of Computer Science, KU Leuven - University of Leuven, Leuven, Belgium

**Keywords:** HIV-1, Protein coevolution, Gag, Protease, Ensemble coevolution system, Sequence-based method

## Abstract

**Background:**

A key challenge in the field of HIV-1 protein evolution is the identification of coevolving amino acids at the molecular level. In the past decades, many sequence-based methods have been designed to detect position-specific coevolution within and between different proteins. However, an ensemble coevolution system that integrates different methods to improve the detection of HIV-1 protein coevolution has not been developed.

**Results:**

We integrated 27 sequence-based prediction methods published between 2004 and 2013 into an ensemble coevolution system. This system allowed combinations of different sequence-based methods for coevolution predictions. Using HIV-1 protein structures and experimental data, we evaluated the performance of individual and combined sequence-based methods in the prediction of HIV-1 intra- and inter-protein coevolution. We showed that sequence-based methods clustered according to their methodology, and a combination of four methods outperformed any of the 27 individual methods. This four-method combination estimated that HIV-1 intra-protein coevolving positions were mainly located in functional domains and physically contacted with each other in the protein tertiary structures. In the analysis of HIV-1 inter-protein coevolving positions between Gag and protease, protease drug resistance positions near the active site mostly coevolved with Gag cleavage positions (V128, S373-T375, A431, F448-P453) and Gag C-terminal positions (S489-Q500) under selective pressure of protease inhibitors.

**Conclusions:**

This study presents a new ensemble coevolution system which detects position-specific coevolution using combinations of 27 different sequence-based methods. Our findings highlight key coevolving residues within HIV-1 structural proteins and between Gag and protease, shedding light on HIV-1 intra- and inter-protein coevolution.

**Reviewers:**

This article was reviewed by Dr. Zoltán Gáspári.

**Electronic supplementary material:**

The online version of this article (doi:10.1186/s13062-014-0031-8) contains supplementary material, which is available to authorized users.

## Background

Recent structural analysis showed that the viral core of HIV-1 particles is formed by capsid hexamers and pentamers through both intra- and inter-protein interactions [1]. HIV-1 capsid protein is encoded by the *gag* gene, which contains matrix, capsid, p2, nucleocapsid, p1 and p6. In a spherical shell of an immature virus, Gag polyproteins are arranged radially in a curved hexameric lattice bound together by protein interactions [2]. The HIV-1 matrix and capsid proteins are cleaved from Gag and reorganized into tubular lattices of mature particles during the protease-mediated proteolytic processing [3]. Mutations near Gag cleavage sites (GCS) can affect the protease binding affinity [4], suggesting that HIV-1 intra- and inter-protein interactions play a key role during the viral life cycle. Previous sequence analyses have reported the association between human HLA alleles and Gag codons [5], intra-protein coevolution in capsid [6] and immunologically vulnerable sectors in Gag [7]. However, a systematic study of HIV-1 intra- and inter-protein coevolution of Gag and protease proteins is largely lacking.

Many studies have revealed position-specific coevolution in HIV-1 proteins using sequence-based methods [5,6,8-12]. For instance, coevolving positions were found to be proximal in capsid structure [6]. HIV-1 drug-resistance mutations in protease, reverse transcriptase and integrase tend to coevolve under the drug selective pressure [8-10,13]. Important coevolving residues were also found in HIV-1 Env [11], Vif [12] and Gag [5]. To model coevolution within and between proteins [11,14,15], position-specific sequence analysis has been used to detect pairs of correlated amino acid positions, so-called statistical couplings [16] (also called co-variations [17] or correlated substitutions [18]). A deep understanding of genetically coevolving residues has enriched our insights in protein folding [17], protein-protein interaction [19], allosteric communication [20] and ligand binding [21] (see review [22]). Since the first sequence-based method was proposed in 1970 [23], more than 30 methods were published and most of them were based on the principle of information theory, physicochemical properties, molecular phylogenetics and Bayesian statistics [15,22,24]. Thanks to the increase of crystalized structures in public databases, the performance of sequence-based methods is usually evaluated based on structural information, such as protein contact map [25], because spatially proximate positions tend to coevolve [26] and sequence evolution is associated with structural dynamics [27]. Nevertheless, state-of-the-art methods in different studies showed significant variability, while evaluation of long-range coevolving residues continues to be difficult in most scenarios [15,22,24].

The supervised ensemble approach in statistics and machine learning aims at creating a robust method through the integration of multiple predictive models [28]. It relies on the philosophy that the aggregation of information from several sources is usually superior to a single individual source for decision-making (e.g. jury, peer-review, voting for political candidates) [28]. Well-known ensemble methods such as random forest [29] and AdaBoost [30] provide robust predictions with outstanding performance in many applications. Other ensemble methods have also been designed for solving various problems [31-33]. For instance, the ensemble machine system XCS was made to improve self-adaptation of evolutionary algorithms [31]. While more than 27 sequence-based methods have been proposed for position-specific coevolution prediction, an ensemble coevolution system that integrates multiple methods to improve the prediction of HIV protein coevolution has not been investigated.

**Figure 1:**
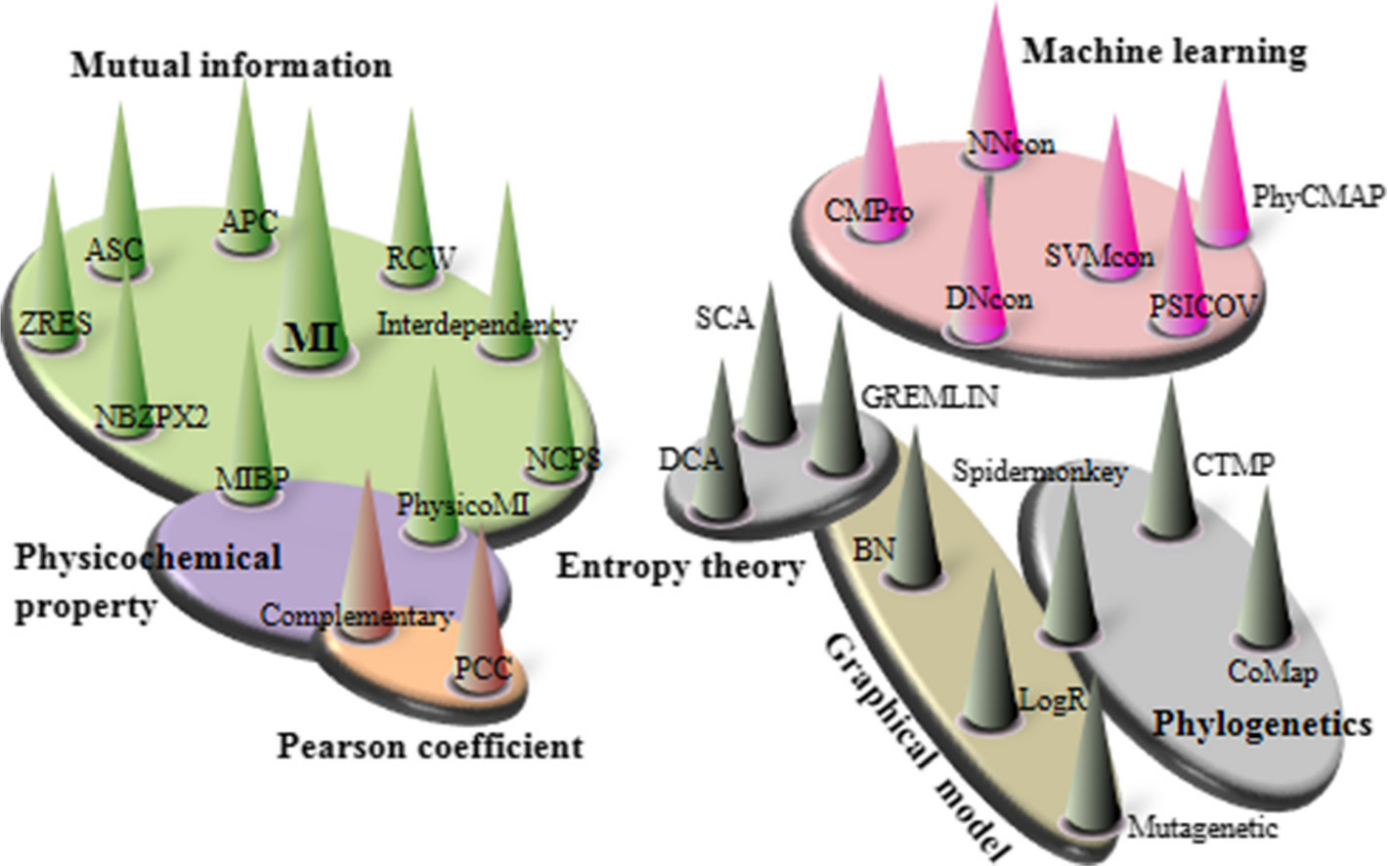
**Correlation-based networks of sequence-based methods.** Seven major methodologies are summarized, including mutual information, machine learning (random forest, support vector machine, neural networks), Pearson coefficient, entropy theory, graphical models (Bayesian networks, singly connected spanning trees, mutagenetic trees), phylogenetic models and physicochemical property models. Methods are represented by cones (see abbreviations in Table 1) and the same color is given to sequence-based methods designed from similar methodologies (e.g. APC used MI as a part of its design, phylogenetic trees are used in Spidermonkey, CTMP and CoMap).

Here, we present the first ensemble coevolution system (ECS) to detect HIV-1 position-specific coevolution by integrating 27 sequence-based methods published between 2004 and 2013 (Table 1, Figure 1). This new software platform allows for parallel coevolution predictions and systematic combinations of sequence-based methods. We collected extensive HIV-1 sequences and experimental and clinical data to evaluate the performance of individual methods and combinations of methods. Using our coevolution system, we identified combinatorial approaches with superior performance at predicting HIV-1 coevolution. We thereafter investigated intra- and inter-protein coevolving positions in HIV-1 Gag and protease using an optimized combinatorial approach that integrated four sequence-based methods.

**Table 1:** **Summary of 27 sequence-based methods in our ensemble coevolution system**

Methods*	**Statistical methodology**	**Updated**	**Ref**
ASC/APC	Mutual information	2007	[34]
BN	Bayesian network	2007	[35]
CTMP	Continuous-time Markov model, phylogenetic tree	2007	[36]
CoMap	Compensation coefficient, phylogenetic tree	2007	[37]
Complementary	AA complementary matrix, Pearson coefficient	2006	[38]
CMPro	2D recursive neural networks	2012	[39]
DCA	Maximum entropy model	2011	[25,26]
DNcon	Deep network, Bolzmann machines	2012	[40]
GREMLIN	Maximum entropy model	2013	[41]
Interdependency	Entropy, mutual information	2004	[42]
LogR	Bayesian networks, APC	2010	[43]
MI	Mutual information	2012	[44,45]
MIBP	Mutual information, physicochemical properties	2011	[46]
Mutagenetic	Maximum likelihood mixed trees	2005	[10]
NBZPX2	Normal binary, ZRES	2012	[44]
NCPS	Mutual information, sequence similarity	2009	[47]
NNcon	Neural networks	2009	[48]
PCC	Mutual information, Pearson’s coefficients	2010	[18]
PSICOV	Sparse inverse covariance	2012	[49]
PhysicoMI	Mutual information, physicochemical properties	2012	[6]
PhyCMAP	Random forest, integer linear programming	2013	[50]
RCW	Mutual information	2007	[51]
Spidermonkey	MCMC Bayesian network, phylogenetic tree	2008	[52]
SCA	Statistical free energy couplings	2009	[53]
SVMcon	Support vector machine	2006	[54]
ZRES	Mutual information	2009	[55]

*A comprehensive description of the methodology and our experimental settings are provided in section 2 of Additional file 1: Text S1.

## Methods

### HIV-1 protein sequence datasets for sequence-based coevolution prediction

As of February 2013, we retrieved 3171 HIV-1 subtype B *gag* and protease nucleotide sequences from the Los Alamos HIV database (http://www.hiv.lanl.gov) (HXB2 nucleotide positions: 1186-2549, one sequence per patient). For each Gag and protease protein, we aligned sequences against the HXB2 reference and manually curated the alignment using Seaview V4.3 [56]. To improve sequence quality, we used the criteria described in our recent study [57] to remove duplicates and sequences with any hypermutation, stop codon, ambiguous nucleotide or subtype misclassification. Afterwards, patient treatment information of the retrieved sequences was obtained from the corresponding sequence publications. Sequence data obtained from treatment-naive patients were used to detect intra-protein statistical couplings given that wild-type HIV-1 protein structures were used for evaluation. Sequence data obtained from patients receiving protease inhibitor (PI) treatment were used to detect inter-protein statistical couplings given that HIV-1 clinical datasets with PI treatment information were used for evaluation. Overall, we obtained five intra-protein sequence datasets: matrix (n = 605), capsid (n = 656), nucleocapsid (n = 768), p6 (n = 1030), protease (n = 1762), as well as two inter-protein sequence datasets, protease-p6 (n = 788) and protease-GCS (Gag cleavage sites) (n = 292).

### Sequence-based statistical methods for position-specific coevolution predictions

We integrated 27 known sequence-based statistical methods (Additional file 1: Text S1) into one software platform for position-specific coevolution predictions. Summarized in Table 1, these methods were mainly designed based on the principles of information theory, phylogenetic analysis, parametric or non-parametric statistical tests, Bayesian maximum likelihood and codon substitution models. Given the inputs of multiple sequence alignments (MSAs) and phylogenetic trees, sequence-based methods predict coevolving residues and rank them according to the method-specific measurements with either parametric or non-parametric statistical tests (Additional file 1: Text S1). The predictions were ranked according to each method. Parameter settings used in our study were either default or optimized according to method manuals or publications (Additional file 1: Text S1). To prepare the inputs of the phylogenetic-based methods, we constructed unrooted maximum likelihood phylogenetic trees using the following procedure. Given the nucleotide MSAs, neighbor-joining phylogenetic trees were obtained by IQPNNI V 3.3 [58] (nucleotide substitution model: general time reversible (GTR) model, bootstrap resampling: 1000 replicates). These neighbor-joining phylogenetic trees were used as starting trees in RAxML V7.0.4, which subsequently optimized the unrooted maximum likelihood phylogenetic trees (nucleotide substitution model: GTRGAMMA, 100 bootstrap replicates) [59].

### HIV-1 protein structural and experimental datasets for evaluating the predictive performance of sequence-based methods

We retrieved PDB data of HIV-1 proteins from the RCSB Protein Data Bank (http://www.pdb.org). The quality of crystalized structures was assessed using PDBREPORT [60] (default parameters). The PDB dataset included: 1HIW (matrix), 3H4E (capsid), 1A1T (nucleocapsid), 2C55 (p6) and 1TW7 (protease). We also collected extensive experimental and clinical data of PI-associated Gag-protease mutations from literature, which was queried in PubMed using the keywords “HIV Gag mutation”, “HIV Gag protease”, “HIV protease mutations Gag”, “HIV Gag evolution” or “HIV protease cleavage”. References in primary studies and reviews were also searched. The data is summarized in Additional file 2: Table S1.

True positives for intra-protein coevolving positions were assessed according to their proximity in protein contact maps. To construct contact maps for each protein, Euclidean distances between the C_β_ atoms of residue pairs were calculated given the atomic coordinates in PDB [50]. In cases where a HIV-1 protein has multiple functional subunits (e.g. matrix, capsid, protease), Euclidean distances between residue pairs were calculated within and between functional subunits and the minimum value for each pair was used for assignment [25]. The predicted intra-protein couplings were assigned as true positives if they were long-range pairs of residues in contact: (1) at least 6 amino acids apart in the sequence [50]; (2) not located at the same alpha-helix or beta-strand secondary structures [48] and (3) less than 8 angstroms between residue pairs on the protein contact map [25]. We used the cutoff of 8 angstroms between C_β_ atoms of residue pairs to detect the residue contact, and a strict cutoff of 5 angstroms was also examined. The predicted intra-protein couplings, which had residues less than 6 amino acids apart in the sequence or were located in the same alpha-helix or beta-strand secondary structures, were not counted during the evaluation. Above criteria were set to evaluate long-range coevolving positions in protein tertiary structures by not counting predictions of neighboring AA positions.

For the protease-p6 and protease-GCS coevolution, the predicted inter-protein residue pairs were considered as true positives if any corresponding Gag-protease mutation patterns were reported in the experimental and clinical datasets (Additional file 2: Table S1). For each row of multiple residue patterns in Additional file 2: Table S1, pairwise combinations of protease-p6 or protease-GCS residues were used for the validation of true positives.

For both intra- and inter-protein predictions, false positives were the couplings in the top-ranked long-range predictions that were not identified as true positives. We did not evaluate negative predictions because the sequence-based methods were not designed to predict residue positions that are not coevolving [22].

### Statistical measurements for method evaluation

Predictions of sequence-based methods were assessed by five statistical measurements.

1. Precision-recall curve (AUC)
  For intra- and inter-protein coevolution predictions, we assessed the area under the precision-recall curve (AUC) [61] as the relative effectiveness of sequence-based methods. Optimized by the binomial model, an unbiased estimator of AUC was calculated by taking into account biases introduced by small sample sizes and class imbalance in favor of negative examples [61]. Notably, AUC is independent of the cutoffs of the top-ranked long-range couplings and is equal to one if all the true positives are ranked higher than the false positives.

(2) Accuracy
  For intra- and inter-protein coevolution predictions, accuracy was calculated as the number of true positives divided by the total number of top-ranked predictions [40,54,62]. Particularly, the accuracy of the L/2 or L top-ranked predictions was evaluated, where L was the number of residue positions in the MSA input. In most instances, the cutoff for positive predictions of coevolving pairs of residues or couplings was set to the L top-ranked couplings. In some instances (mentioned specifically), it was set to the L/2 top-ranked predictions [62]. Thus, positive predictions for coevolution are the L top-ranked couplings, unless it is specified that L/2 is used as a cutoff.

(3) Harmonic distance
  For intra-protein coevolution predictions, the harmonic distance *X*_*d*_ was measured as a weighted harmonic average difference between the Euclidean distance distribution of the predicted couplings and the all-pair Euclidean distances [50,62]. Being popular in Critical Assessment of Protein Structure Prediction (CASP), the harmonic distance *X*_*d*_ is defined as: ${X}_d={\displaystyle {\sum}_{n=1}^{15}\left(P\left({d}_n\right)-P\left({a}_n\right)\right)/n}$, where *P*(*d*_*n*_) is the percentage of predicted couplings with Euclidean distances between 4(n − 1) and 4n, *P*(*a*_*n*_) is the percentage of all contact pairs with Euclidean distances between 4(n − 1) and 4n [50]. A higher value of the harmonic distance *X*_*d*_ indicates a better prediction performance of a method. Note that the harmonic distance does not impose a distance cutoff for the evaluation of coevolution predictions.

(4) Average Euclidean distance
  For intra-protein coevolution predictions, average Euclidean distance was measured for the top-ranked long-range couplings using the Cβ-Cβ Euclidean distances [25]. It is defined as: ${\displaystyle {\sum}_{i=1}^L Dist\left({C}_i^1,{C}_i^2\right)/L}$, where L is the number of top-ranked couplings, ${C}_i^1$ and ${C}_i^2$ are two residue positions in the i^th^ top-ranked long-range coupling. For evaluation purposes, the number of top-ranked couplings predicted by individual methods was set to L/2 or L [62]. A lower value of average Euclidean distance indicates better prediction performance of a method. Note that the average Euclidean distance does not impose a distance cutoff for the evaluation of coevolution predictions.

(5) Jaccard and association coefficients
  To quantify the predictive heterogeneity of sequence-based methods, Jaccard and association coefficients were calculated between the top-ranked long-range couplings predicted by different sequence-based methods. Given two coupling sets X and Y, Jaccard and association coefficients are defined as |*X* ∩ *Y*|/|*X* ∪ *Y*| and |*X* ∩ *Y*|/min(|*X*|, |*Y*|), respectively [63].

### Ensemble coevolution system (ECS)

**Figure 2:**
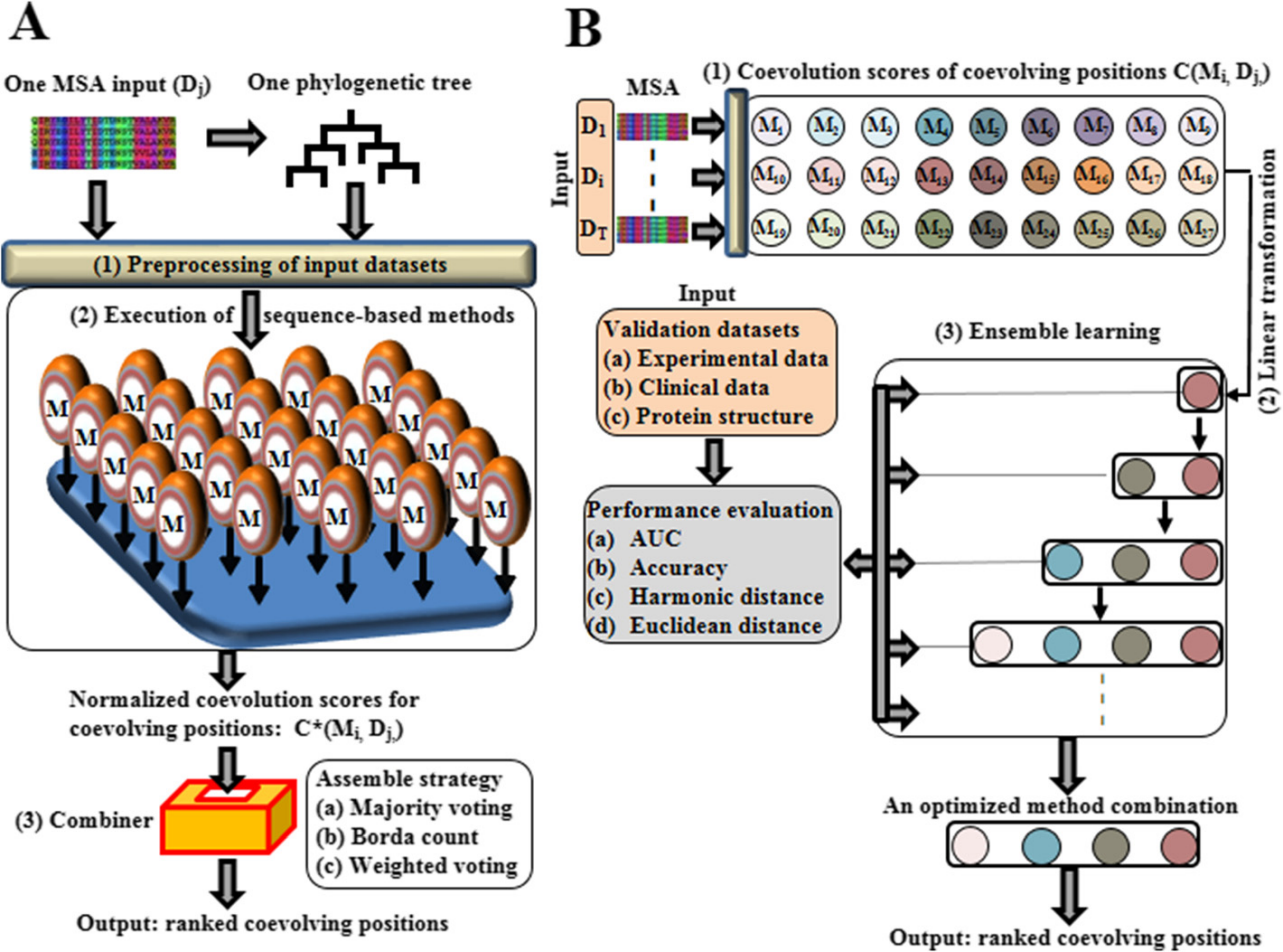
**Schematic view of ensemble coevolution system. (A)** Workflow of coevolution prediction. Input data: a multiple sequence alignment dataset D_j_ and one phylogenetic tree constructed using D_j_. (1) Preprocessing of input datasets. The method-specific input formats are preprocessed and imported into individual sequence-based methods *M*_*i*_(*i* = 1, …, 27). (2) Execution of sequence-based methods. Given the sequence-based method M_i_ and the sequence dataset D_j_, coevolution scores of coevolving positions are normalized and exported into the matrix C*(M_i_, D_j_). In addition, normalized co-evolution scores are ranked. (3) Combiner. Given a chosen combination of sequence-based methods, coevolution scores of predicted coevolving positions are assembled through the combiner, which provides the assemble strategies such as majority voting, Borda count and weighted voting. **(B)** Workflow of our procedures that optimize the combination of sequence-based methods. Input data: inputs of multiple MSAs are processed by sequence-based methods (see **(A)**). The validation datasets (e.g. experimental and clinical data) are also prepared for the method evaluation. Coevolution scores of ranked coevolving pairs in C(M_i_, D_j_) are collected after applying the sequence-based method M_i_ to the sequence dataset D_j_. (1) Linear transformation. Coevolution scores are linearly transformed between 0 and 1. (2) Ensemble learning. A heuristic algorithm identifies the combination of sequence-based methods with improved prediction performance (Additional file 1: Text S1). Each circle represents a single method and the combination of different methods is demonstrated in a group of colored circles. Using the validation datasets, prediction performance is evaluated (e.g. AUC) for the ranked statistical couplings assembled from the corresponding method combination. When adding a new method will not improve the prediction performance, the learning procedure stops and an optimized method combination is identified. Using the identified method combination, coevolving pairs are predicted as in **(A)** and returned as outputs.

To provide robust position-specific coevolution predictions, we designed an ensemble coevolution system by integrating 27 sequence-based methods published in the last decade (Table 1). Inspired by the ensemble principle [64], ECS’s workflow includes: (1) inputs of MSAs and their corresponding phylogenetic trees, (2) execution of sequence-based methods, (3) a method combiner which integrates prediction results from different methods. Figure 2 shows the schematic overview of ECS and its model is described as follows. Suppose we have a set of sequence-based methods, denoted as *M* = {*M*_1_, *M*_2_, …, *M*_*N*_} and multiple sequence datasets, denoted as: *D* = {*D*_1_, …, *D*_*T*_}, where N is the number of methods (N = 27 in our study) and T is the number of sequence datasets (T = 7 in our study).

### Execution of sequence-based methods

Sequence-based methods are applied using parallel computation. Each method quantifies the coevolution score for every possible pair of amino acid positions. Given a dataset *D*_*j*_, the method *M*_*i*_ quantifies a coevolution score for the statistical coupling between the n^th^ and the m^th^ positions (*n*, *m* ∈ {1, …, *L*}), where L is the number of amino acid positions in *D*_*j*_. The higher the score, the higher the statistical significance based on the method-specific measurements. This process generates a scoring matrix *C*(*M*_*i*_, *D*_*j*_) which has at most L × L pairs. The coevolution scores in *C*(*M*_*i*_, *D*_*j*_) are then linearly transformed between 0 and 1 (C*(*M*_*i*_, *D*_*j*_) = [*C*(*M*_*i*_, *D*_*j*_) − min(*C*)]/[max(*C*) − min(*C*)]), where the higher the score, the higher the statistical significance. Given the method *M*_*i*_ and the dataset *D*_*j*_, C*_n,*m*_(*M*_*i*_, *D*_*j*_) is the normalized coevolution score between the n^th^ and the m^th^ positions. For each MSA evaluated by each method, the normalized coevolution scores in the scoring matrix are ranked with the highest score being the top ranked (Additional file 1: Text S1, Section 2).

### Combiner

Users can choose any individual methods to combine, or use three implemented assemble strategies (majority voting, Borda count, weighted voting) [64]. Majority voting and Borda counting use the ranked scores; weighted voting first combines the normalized co-evolution scores and then ranks the combined scores. Specifically, for the majority voting, the combiner outputs the predicted coevolving residues if they were predicted in the (L or L/2) top-ranked predictions by more than half of the 27 sequence-based methods. For the Borda count, the combiner outputs only the coevolving residues if they were predicted in the (L or L/2) top-ranked coupling predictions by all the 27 sequence-based methods. For weighted voting, ranking is done after collecting the weighted votes (see the detailed description in Additional file 1: Text S1).

### Identification of method combinations using a heuristic algorithm

Using validation datasets to evaluate the method performance, we proposed a heuristic algorithm to optimize a method combination. Given a performance measurement *f* (e.g. AUC), *f*(C*(Ω, *D*_*j*_)) measures the statistical performance of the method combination Ω applied to the dataset *D*_*j*_. To identify an optimized combination of methods, an objective function *F*(Ω, *D*) is defined by a linear function [65]:

$$ F\left(\varOmega, D\right)={\displaystyle \sum_{j=1}^T{u}_j\times f\left({C}_{n,m}^{*}\left(\varOmega, {D}_j\right)\right)} $$

Where *u*_*j*_ is the weight of the training dataset *D*_*j*_ contributed to the objective function. All datasets are treated equally if every *u*_*j*_ equals to 1.

Based on the objective function, an optimized combination of methods, denoted as Ω^+^, is obtained by ${\varOmega}^{+}=\underset{\varOmega \subseteq M}{ \max }F\left(\varOmega, D\right)$. Given the 27 known sequence-based methods, we aimed at identifying a method combination Ω^+^ to achieve a high prediction performance, preferably combining only a small number of methods. The reason for this is twofold. Firstly, some coevolution methods are computationally heavy. Secondly, it is hard to implement and apply an ensemble system integrating many complex methods. To simplify the optimization procedure, we also assumed that all training datasets contributed equally (*u*_*j*_ = 1) and sequence-based methods contribute equally in a method combiner when selected (*w*_*i*_ equals to 1 or 0). Inspired by the forward selection and backward elimination approach [66], we designed a heuristic algorithm to identify the smallest method subset that maximizes the objective function. Additional file 1: Text S1 clarifies this heuristic algorithm with more mathematical details. Here we provide an overview of the underlying principle.

Our heuristic algorithm begins with the independent predictions of the 27 sequence-based methods applied on the MSA inputs (Figure 2). For each method with a MSA input, statistical couplings in the scoring matrix are ranked according to the method-specific significance measurements (Additional file 1: Text S1, Section 2). In the next step, the forward selection each time visits all methods but only adds the method with the largest increase in performance into the method subsets and assembles the coupling predictions for evaluation. The procedure ends when adding a method does not further improve the best performance score. Similar to forward selection, the backward elimination is performed (see Additional file 1: Text S1). To evaluate the performance of the score, AUC is used because it is a statistical measurement independent of the cutoffs of the top-ranked predictions.

## Results

### Estimate HIV-1 coevolution using a new ensemble coevolution system (ECS)

From the Los Alamos database, we retrieved 3171 nucleotide sequences of HIV-1 subtype B Gag and protease, resulting in five intra-protein datasets (matrix, capsid, nucleocapsid, p6, protease) and two inter-protein datasets (protease-p6, protease-GCS). These HIV-1 datasets individually contained more than 200 sequences and the percentage of gaps in each sequence dataset was less than 0.22% (Additional file 2: Table S2). In agreement with our previous study [57], the amino acid diversity of our sequence datasets was between 4.57% and 14.30% (Additional file 2: Table S2). We calculated protein contact maps based on the Euclidian distance between amino acids in the protein structures of matrix, capsid, nucleocapsid, p6 and protease. A Euclidian distance of less than 8 Å between residue pairs was considered as a biological measure of intra-protein coevolution [25]. We also performed a literature search of associated Gag and protease residues to identify inter-protein couplings confirmed by experimental and clinical studies. These data obtained from protein structure and literature review was used to validate true positive predictions of statistical couplings generated by sequence-based methods. We then designed an ensemble coevolution system (ECS) which integrates 27 sequence-based methods published between 2004 and 2013 (Figure 1, Table 1). Thereafter, we designed a heuristic algorithm to optimize the combination of sequence-based methods, which were evaluated by AUC (see Methods). Given our seven HIV-1 sequence datasets, this heuristic algorithm identified an optimized method combination, so-called CNPR, for the prediction of HIV-1 intra- and inter-protein coevolution (see section 1 of Additional file 1: Text S1). This CNPR combination comprised of four known methods (CMPro [39], NCPS [47], PhyCMAP [50] and RCW [51]), weighted equally (see section 1 in Additional file 1: Text S1).

### CNPR outperforms 27 known sequence-based methods in detecting HIV-1 coevolution

**Figure 3:**
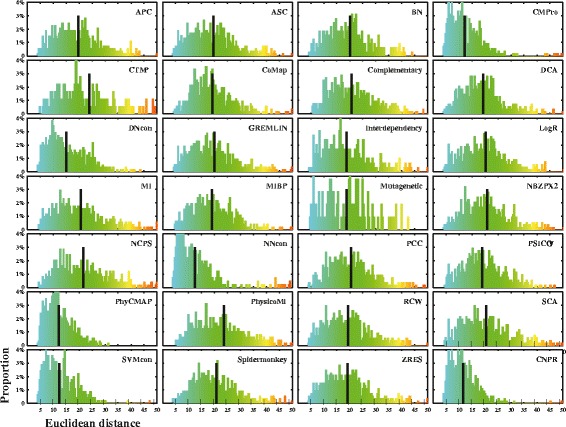
**Evaluation of sequence-based methods in predicting HIV-1 intra-protein coevolution.** Distribution plots of Euclidean distance between position pairs in the L top-ranked couplings predicted by individual methods. X- and y-axes indicate the estimated Euclidean distances and the percentage of top-ranked couplings, respectively. Black lines indicate the mean values of Euclidean distances calculated using the L top-ranked couplings. For any method, a lower value of average Euclidean distance indicates that predicted coevolving pairs are in proximity, showing a better prediction performance.

We found that CNPR outperformed each of the 27 sequence-based methods in the prediction of HIV-1 intra- and inter-protein coevolution using four statistical measurements. All the 27 methods and the CNPR combination were evaluated and ranked for 7 HIV-1 sequence datasets, displayed in Additional file 2: Figure S1. Firstly, CNPR achieved the best average ranking (2.07) over the 7 datasets followed by CMPro (5.71) and PhyCMAP (6.87) based on the AUC measurement (Table 2, Additional file 2: Table S3). Secondly, CNPR achieved the highest average accuracies over the 7 datasets for both the L/2 and L top-ranked predictions (average accuracy = 0.35, 0.27, respectively) (Additional file 2: Table S4). Comparing CNPR to the second best method NNcon, average accuracies over the 7 datasets for the L/2 and L top-ranked predictions increased by 0.061 (17.6%) and 0.031 (11.5%), respectively (Table 2, Additional file 2: Table S4). Thirdly, we measured the harmonic distance X_d_ on the five intra-protein datasets. CNPR reached the second (X_d_ = 0.78) and the first ranking (X_d_ = 0.66) on the L/2 and L top-ranked predictions, respectively (Table 2, Additional file 2: Table S5). Fourthly, the L top-ranked long-range predictions of CNPR had the lowest average Euclidean distances (mean Euclidean distance: 11.52 Å, 95% confidence interval: 4.64-20.85 Å, Figure 3). The L/2 top-ranked long-range predictions of CNPR had the second lowest average Euclidean distances (mean Euclidean distance: 10.14 Å, 95% CI: 4.53-17.43 Å).

**Table 2:** **Performance of sequence-based methods in detecting HIV-1 protein coevolution**

**Method**	**Area-under-curve (AUC)**							**Average accuracy**		**Average harmonic distance**		**Average Euclidean distance**	
	**MA**	**CA**	**NC**	**p6**	**PR**	**p6-PR**	**CSM-PR**	**L/2**	**L**	**L/2**	**L**	**L/2**	**L**
APC	0.57	0.55	0.59	0.71	0.57	0.62	0.66	10.8%	8.6%	0.039	0.027	17.38	18.6
ASC	0.56	0.53	0.59	0.75	0.59	0.63	0.62	15.0%	11.7%	0.051	0.028	16.41	18.69
BN	0.71	0.55	0.62	0.69	0.75	0.54	-*	5.9%	5.2%	0.009	0.008	19.94	20.13
CMPro	0.75	0.66	**0.85** ^**#**^	0.76	0.74	0.68	0.72	28.9%	22.5%	**0.166**	0.13	**10.05**	11.77
CTMP	0.54	0.52	-	-	0.57	0.69	-	3.3%	3.3%	0.004	0.004	16.98	16.98
CoMap	0.52	0.52	0.61	-	0.55	-	0.5	3.9%	4.3%	0.029	0.029	16.85	17.14
Complementary	0.52	0.52	0.57	0.54	0.53	0.53	0.55	4.0%	4.7%	0.008	0.003	19.08	20.01
DCA	0.55	0.55	0.59	0.78	0.51	0.64	0.67	9.2%	7.1%	0.03	0.023	17.43	18.45
DNcon	0.5	0.51	0.66	-	0.61	-	0.77	16.5%	11.3%	0.093	0.07	13.66	15.11
GREMLIN	0.56	0.54	0.6	0.81	0.6	0.6	0.63	13.8%	9.5%	0.04	0.024	17.14	18.77
Interdependency	0.63	0.58	0.68	-	0.66	-	-	7.3%	7.0%	0.028	0.026	18.4	18.58
LogR	0.55	0.54	0.54	0.8	0.55	0.58	0.55	11.4%	8.3%	0.024	0.015	18.44	19.32
MI	0.51	0.54	0.58	**0.84**	0.58	0.81	0.79	17.9%	12.6%	0.043	0.026	17.6	18.96
MIBP	0.57	0.5	0.57	0.67	0.53	0.62	0.7	4.5%	5.3%	0.021	0.023	17.8	18.12
Mutagenetic	0.53	0.66	0.71	-	0.64	0.86	0.6	15.9%	15.9%	0.027	0.027	19.13	19.13
NBZPX2	0.56	0.52	0.54	0.55	0.54	0.51	0.5	6.1%	5.2%	0.011	0.005	19.51	20.2
NCPS	0.58	0.51	0.54	0.83	0.56	0.86	0.83	17.0%	11.6%	0.018	0.011	19.37	20.27
NNcon	0.68	**0.72**	0.78	-	**0.78**	-	-	28.6%	23.8%	0.148	**0.132**	11.25	12.01
PCC	0.53	0.56	0.55	-	0.51	0.54	0.61	7.0%	5.0%	0.013	0	18.63	20.2
PSICOV	0.56	0.58	0.54	0.55	0.51	0.51	0.53	8.4%	6.2%	0.016	0.012	18.63	18.79
PhyCMAP	**0.76**	**0.7**	0.72	0.65	0.72	0.8	0.55	19.4%	17.2%	0.118	0.107	11.83	12.55
PhysicoMI	0.61	0.56	0.52	**0.84**	0.5	0.72	0.64	7.1%	4.6%	0.009	−0.001	20.46	21.06
RCW	0.54	0.53	0.58	0.82	0.56	0.8	0.78	12.3%	10.9%	0.044	0.032	16.88	18.12
SCA	0.54	0.54	0.56	0.53	0.58	0.77	0.77	15.7%	10.8%	0.027	0.016	18.26	19.08
SVMcon	0.71	**0.73**	0.67	-	**0.77**	-	-	24.6%	18.3%	0.14	0.111	11.42	12.65
Spidermonkey	0.58	0.55	0.63	0.67	0.52	0.51	0.57	6.5%	5.7%	0.018	0.01	18.89	19.77
ZRES	0.56	0.53	0.59	0.73	0.56	0.61	0.68	12%	10.7%	0.046	0.032	16.65	18.08
CNPR	0.75(2.5)	0.7(3.5)	0.83(2)	**0.84(1)**	0.77(2.5)	**0.87(1)**	**0.88(1)**	**34.7%(1)**	**26.9%(1)**	0.155(2)	**0.132(1.5)**	10.14(2)	**11.52(1)**

*AUC was not evaluated due to the lack of long-range couplings predicted. ^#^For each column, the numbers in bold indicate methods with the best score among the 28 methods. The ranking of CNPR for each dataset is provided in brackets (see others in Additional file 2: Table S3). Ranking numbers in decimals are results from the average rankings (see examples in Additional file 2: Table S3). Four statistical measurements (AUC, accuracy, harmonic distance, Euclidean distance) are defined in Methods. For the latter 3 methods, the L or L/2 top-ranked predictions were compared and the average scores over the 7 HIV-1 datasets were listed (see performance evaluation per method per dataset in Additional file 2: Table S4-S6).

### Sequence-based methods cluster according to their methodology

**Figure 4:**
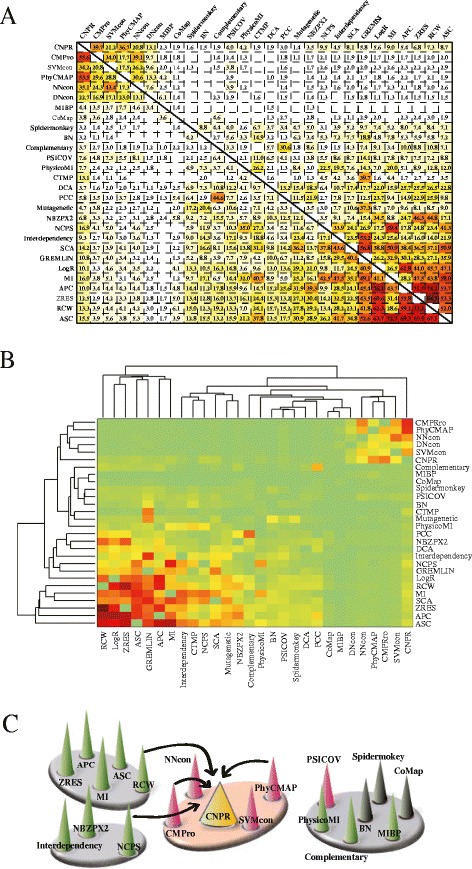
**Prediction similarity of sequence-based methods and method clustering. (A)** Jaccard and association coefficients between the L top-ranked couplings predicted by 28 sequence-based methods. **(B)** Hierarchical clustering analysis of Jaccard (bottom) and association (left) coefficients between the 28 sequence-based methods. The heat-map distinguishes the smallest (green) and highest (red) coefficients between the 28 sequence-based methods. **(C)** Four method clusters identified commonly by the two clustering trees in **(B)**. The arrows connect four methods (CMPro, NCPS, PhyCMAP, RCW) integrated in CNPR. Methods designed based on mutual information are colored in green, phylogenetics in grey, machine learning in pink.

We hypothesized that methods designed from a similar underlying methodology may output similar predictions. To measure the prediction similarities between the sequence-based methods, we calculated Jaccard and association coefficients for the top-ranked predictions between every two methods applied to the 7 HIV-1 datasets. CNPR shared the highest Jaccard and association coefficients with CMPro and PhyCMAP among the 27 sequence-based methods (Figure 4A). This observation was independent of the prediction cutoffs (Additional file 2: Figure S2). Our hierarchical clustering analysis on the Jaccard and association coefficients revealed four clusters, each of which contained methods generating similar predictions (Figure 4B). Among the four methods integrated in CNPR, CMPro and PhyCMAP shared the same cluster with CNPR, while NCPS and RCW were individually located in the other two clusters (Figure 4C). Moreover, 15 out of 19 methods grouped in the method network were designed using similar methodologies, indicating that methods designed from a similar methodology tend to generate similar predictions.

### Detection of HIV-1 intra-protein coevolution

Using HIV-1 sequence datasets, we applied CNPR to investigate coevolution within each HIV-1 protein. In this section, the predicted coevolving residues refer to the L top-ranked long-range couplings predicted by CNPR.

**Figure 5:**
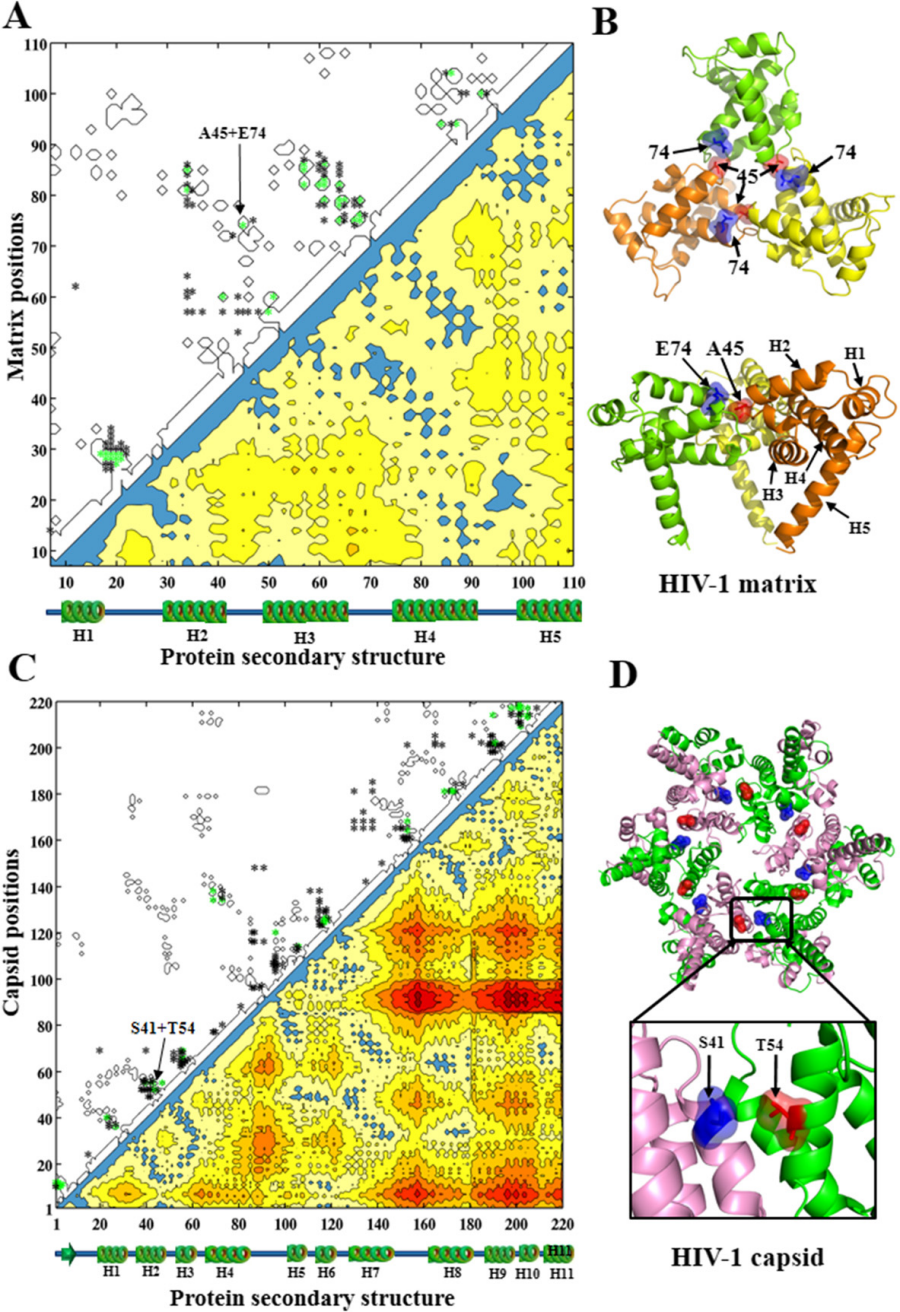
**Intra-protein couplings of HIV-1 matrix and capsid predicted by CNPR. (A)** Contact map of HIV-1 matrix protein and intra-protein coevolving pairs predicted by CNPR. Five helices (H1 to H5) and random-coil secondary structures are aligned to the x-axis. At the bottom right, protein contact map is colored according to the Euclidean distances between two amino acid positions in the 3D structure. Coevolving pairs are colored blue if Euclidean distances were less than 8 Å, otherwise gradient from yellow to red. At the upper left, the predicted coevolving residues are marked as asterisks. Green asterisks indicate true positive couplings falling within the black contours of protein contact map. **(B)** Cartoon representation of HIV-1 matrix structure. The predicted intra-subunit coupling between the residues A45 and E70 is annotated. PDB code: 1HIW. **(C)** Contact map of HIV-1 capsid protein and intra-protein coevolving pairs predicted by CNPR. Figure captions are the same as in **(A)**. **(D)** Cartoon representation of HIV-1 capsid hexamer with 6 identical subunits. The predicted intra-subunit coupling between the residues A42 and T54 is annotated. PDB code: 3H4E. The intra-protein couplings predicted by all 28 methods in HIV-1 proteins are shown in Figure S4-S7. Visualization software: PyMOL V1.5 (http://www.pymol.org/).

Of the 132 predicted coevolving residues in the HIV-1 matrix protein (L = 132), 30.3% were true positives (thus accuracy equals 30.3%), 56.8% were between two helix structures (helix-to-helix), 40.9% involved one position in the third (positions: 47-67) and 50.1% one position in the fourth (positions: 73-90) helix structures (Figure 5A). The average Euclidean distance of the predicted coevolving residues was 9.97 Å compared to 19.22 Å between all residue pairs. As an example, CNPR predicted a true positive coupling A45 + E74 (Euclidean distance: 5.69 Å) within the subunit interaction interfaces involving with the third and the fourth random-coil structures in the matrix protein (Figure 5B).

Of the 231 predicted coevolving residues in capsid (L = 231), 21.2% were true positives, 9.5% were between two random-coil structures (coil-to-coil) and 52.8% were helix-to-helix couplings involving heavily 4 of the 11 helices (helix 3: 16.9%, helix 7: 15.2%, helix 11: 19.1%, helix 12: 18.6%) (Figure 5C). Average Euclidean distance of the predicted coevolving residues was 12.78 Å compared to 26.07 Å between all residue pairs. CNPR also predicted the capsid coupling S41 + T54 (7.22 Å) within the subunit interaction interfaces located between N-terminal domains (NTDs) (Figure 5D).

**Figure 6:**
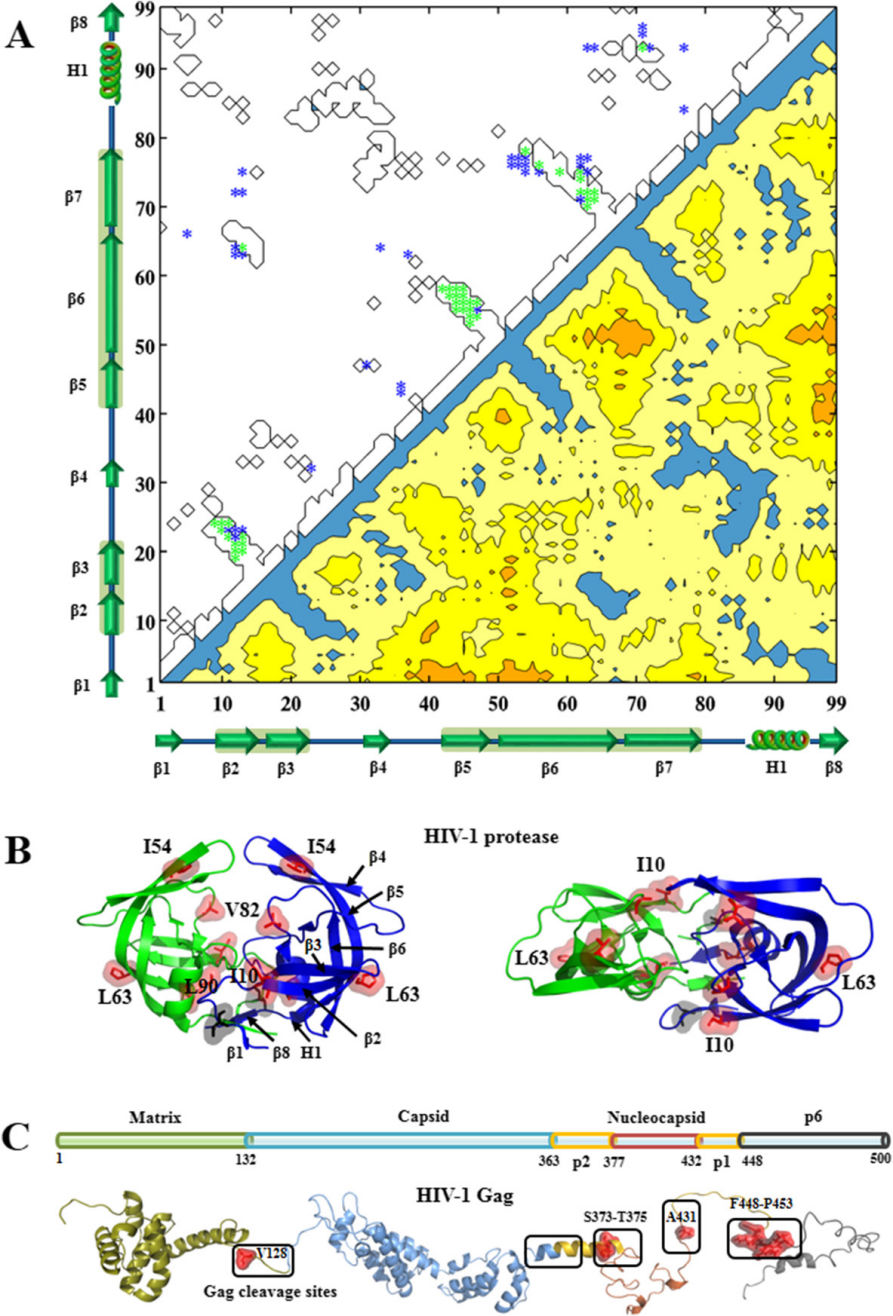
**Intra-protein couplings of HIV-1 protease and Gag cleavage sites in the protein structures. (A)** HIV-1 protease coevolving pairs predicted by CNPR. The contact map of HIV-1 protease (bottom right) and the predicted coevolving pairs (top left) are illustrated. Green dots indicate true positive predictions in the protein contact map. The random-coil (e.g. L1-L2), beta-strand (e.g. β1-β8) and helix (e.g. H1) secondary structures are shown along the x- and y-axes. **(B)** Top and side views of the residue positions (T4, L10, I54, L63, V8, L90) in HIV-1 protease. One helix (e.g. H1) and eight beta-strand (e.g. β1-β8) secondary structures are also shown. **(C)** Gag cleavage sites in the 3D protein structure of Gag proteins. Gag cleavage sites are annotated in boxes and amino acid positions (V128, S373-T375, V431, F448-P453) are colored in red. PDB code: 1HIW (matrix), 3NTE (capsid), 1U57 (p2), 2M3Z (nucleocapsid), 2C55 (p6). Visualization: PyMOL V1.5 (http://www.pymol.org/).

Of the 99 predicted coevolving residues in protease (L = 99), 44.4% were true positives, 79.8% were between two beta-strands (strand-to-strand), 6.1% were coil-to-coil couplings. Many predicted coevolving residues involved one position in the fourth (25.3%), the fifth (52.5%) and the sixth beta-strands (44.4%) (Figure 6A). Average Euclidean distance of the predicted coevolving residues was 9.87 Å compared to 17.61 Å between all pairwise residues. CNPR did not detect couplings between two monomers in protease.

Regarding the coevolution predictions in nucleocapsid (L = 52, Additional file 2: Figure S3) and p6 (L = 55), 100% and 67.05% were in the random-coil structures, respectively. No couplings between subunits were detected since both nucleocapsid and p6 are monomers.

### Detection of HIV-1 inter-protein coevolution

We applied CNPR to investigate HIV-1 inter-protein coevolution using the protease-p6 and protease-GCS sequence datasets. In this section, the predicted coevolving residues refer to the L top-ranked long-range couplings predicted by CNPR. Of the 151 predicted protease-p6 couplings (L = 151), 17.9% were true positives, 21.8% were located in the coil-to-coil couplings, 53.3% were coil-to-strand couplings, 28.5% involved 5 protease positions (T4, L10, L63, V82, L90), 76.2% involved either protease cleavage sites Q450-P453 or protease-p6 overlapping positions (Gag positions: S489-Q500) (Figure 6), 58.9% had either Gag or protease positions identified in experimental studies (Additional file 2: Table S1).

Of the 149 coevolving residues predicted between protease and GCS (L = 149), 28.9% were true positives, 84.6% had either Gag or protease positions identified in the experimental and clinical studies, 25.5% were coil-to-coil couplings, 68.5% were the coil-to-strand couplings, 25.5% involved 4 protease positions (L10, I54, L63, V82), 93.3% had GCS positions V128, S373-T375, A431 and F448-P453. Of interest, protease positions L10, I54, L63 and V82 are located near the protease active site (Figure 6).

## Discussion

To our knowledge, this study presents the first ensemble coevolution system (ECS) to predict the position-specific coevolution in HIV-1 proteins. Ensemble systems with robust predictions have been applied previously [29-33,67-70]. For instance, a super learner was created to improve the prediction of HIV-1 drug susceptibility using a set of machine learning algorithms [67]. As shown in our study, an ensemble approach can provide robust predictions of position-specific coevolution when different sequence-based methods predict different coevolving residues. The problem of discordant predictions has been reported previously. For instance, a significant variability in the performance of 13 sequence-based methods was shown using simulated and experimental MSAs [44]. A review which summarized the performance of 9 sequence-based methods also demonstrated different predictions of sequence-based methods [24]. The aim of our study was to detect HIV-1 intra- and inter-protein coevolution using the ensemble learning strategy. For this reason, our study presents a new ensemble coevolution system that integrates 27 sequence-based methods published in the last decade.

### An ensemble approach outperforms individual sequence-based methods in detecting HIV-1 coevolution

Armed with our coevolution system, HIV-1 coevolving residues were predicted and the true positive predictions were evaluated using independent evaluation datasets. For HIV-1 intra-protein coevolution, we used protein contact maps to evaluate coevolving residues in close proximity within protein structures. For HIV-1 inter-protein coevolution, we evaluated protease-GCS and protease-p6 couplings using the results reported in literature, summarized in our experimental and clinical datasets (Additional file 2: Table S1).

We designed a heuristic algorithm to identify CNPR − a combination of four methods (CMPro [39], NCPS [47], PhyCMAP [50], RCW [51]). We found that CNPR outperformed any of the 27 individual methods in the prediction of HIV-1 intra- and inter-protein coevolution. Moreover, CNPR was mostly ranked first or second using four measurements (AUC, accuracy, harmonic distance, Euclidean distance) for performance evaluation (Table 2, Additional file 2: Table S3). Interestingly, our clustering analysis showed that the four methods in CNPR originated from three method clusters (Figure 4C), suggesting that combining methods designed from different principles may establish a superior ensemble method [64]. This observation was supported by a recent study, showing that the combination of PSICOV and plmDCA can improve the prediction performance of either PSICOV or plmDCA alone [71]. Our heuristic algorithm used weighted voting as a combination strategy. During the design of our algorithm, we examined two other ensemble strategies, namely majority voting (predictions supported by more than 50% of the considered methods) and Borda count (predictions made by all the methods) [28], both of which yet failed to outperform individual methods (average rankings beyond the top 10, data not shown). Other advanced ensemble algorithms may provide alternative strategies with promising performance.

Our study aimed at comparing sequence-based methods as accurately as possible, but five factors may limit our comparisons: (1) protein contact maps obtained from crystallized structures may reveal most but not all coevolving residues. The contact map evaluation assumes that a destabilizing mutation at one position is compensated for a mutation at the other position in contact, probably due to biochemical constrains (i.e. charge, volume and polarity) [72]. Yet, two residues that are in close contact may not always coevolve [72,73]. Coevolving residues are not necessarily in physical contact due to protein dynamics in various contexts [16,20,74]. Despite these, protein contact maps remain the most popular strategy to evaluate true positive predictions in position-specific coevolution [22]. While our evaluations of the predictive performance mainly used 8 angstroms as the threshold of contact distance, our method CNPR also achieved top rankings when a strict cutoff value of 5 angstroms was applied (Additional file 2: Table S8). (2) Default parameters of sequence-based methods were mostly applied in our study but the optimization of parameters adapted to the HIV-1 datasets may provide better predictions. For instance, phylogenetic methods usually require high computation and memory consumption, forcing less optimized parameters to be used [22]. (3) Experimental and clinical studies provide some but not complete data to evaluate all true positive predictions. (4) The power of position-specific methods relies on the number of mutations observed in MSA inputs, limiting the prediction of coevolution occurring at highly conserved residues [75]. (5) Besides the above factors, phylogenetic bias, indirect coupling and stochastic effects can affect coevolution prediction [44,49].

### HIV-1 intra-protein coevolution detected by the method combination CNPR

We applied the method combination CNPR to investigate HIV-1 intra-protein coevolution in Gag and protease proteins, which play important roles in HIV-1 morphogenesis [1]. While CNPR was selected because it had the highest number of true positive predictions, we also found other interesting observations among the predicted co-evolving residues.

In our analysis of matrix intra-protein coevolution, 30.3% of the predicted coevolving residues were true positives − a promising accuracy which represents a three-fold enrichment compared to a random prediction (average percentage of residue pairs in contact: 10.5%, see Additional file 2: Table S7). Most predicted coevolving residues were located between the third (positions: 47-67) and the other helices in matrix, suggesting a role of the third helix in viral assembly. Previously, positions 54 and 68 were found to be important for matrix assembly [76]. Many positive predictions had residue positions between 45-47 and 68-74 (e.g. A45 + E74), formed as two short random-coil loops in the matrix protein. As illustrated in Figure 5B, these two loops are in contact and located in the subunit interaction interface of the matrix trimeric complex. Matrix mutations near this interaction interface can alter the subunit interactions, resulting in the impairment of viral assembly and Env incorporation [77,78].

In our analysis of capsid intra-protein coevolution, 21.2% of the predicted coevolving residues were true positives − a four-fold enrichment compared to a random prediction (the average percentage of residue pairs in contact: 5.6%, Additional file 2: Table S7). Half (52.8%) of the long-range coevolving residues were found within helices, especially the helices 3, 7, 11 and 12 (Figure 5D). These helices near the capsid intra- and inter-subunit interaction interfaces play a key role in the capsid assembly and stability [1,79-81]. The helices 3, 4 and 7 in the N-terminal domain (NTD) and helices 8 and 11 in the C-terminal domain (CTD) are essential for NTD-CTD interactions in the capsid hexamer [80-83]. When considering predicted coevolving residues within capsid subunits, E71 + L111 was previously predicted using a dataset of HIV-1, HIV-2 and SIV sequences [6]. In our analysis using CNPR, the predicted coupling S41 + T54 was ranked higher than E71 + L111. Moreover, the Euclidean distance between S41 and T54 (7.22 Å) is shorter than that between E71 and L111 (9.85 Å).

In our analysis of protease intra-protein coevolution, 44.4% of the predicted coevolving residues were true positives − a four-fold enrichment compared to a random prediction (the average percentage of residue pairs in contact: 12%, Additional file 2: Table S7). Most statistical couplings (79.8%) were between beta-strand structures; particularly, the second, third and fifth beta-strand structures. Coevolving residue clusters in these beta-strand structures have been reported previously [84,85].

Besides the intra-protein coevolution reported here, other coevolution events in HIV-1 Gag have also been reported. For instance, five groups of Gag positions were coevolving under multidimensional constraints and one of these groups contains positions in the capsid N-terminal helices [7]. Our coevolution analysis on HIV-1 capsid also identified statistical couplings at the N-terminal helices near the subunit interaction interface. In another study, phylogenetic dependency networks were used to infer patterns between human leukocyte antigen (HLA) alleles and HIV-1 Gag residues, resulting in the prediction of 149 couplings between HLA alleles and Gag codons, as well as 1386 couplings within matrix and capsid [5]. Our study observed different predictions within matrix and capsid, possibly because we focused on HIV-1 subtype B, while the coevolution analysis in [5] used a mixed subtype B and C dataset.

Our previous study showed a high amino acid diversity of Gag (18.38%) between subtypes B and C [57], which may lead to different coevolution predictions in sequence-based analyses [86]. Using the alternative sequence datasets of subtypes B and C from [57], position 280 in Capsid was predicted by CNPR to coevolve with many positions (e.g. 138, 146, 147) in subtype B, but not in subtype C. Note that at amino acid position 280 (Gag index), the prevalence of amino acids T (68.8%) and V (22.1%) in subtype B is clearly different from two most common amino acids T (1.8%) and V (97.9%) in subtype C [57]. This indicates that position 280 is much more conserved in subtype C than subtype B, thus the power to detect a significant signal is lower in subtype C. Besides position 280, we also detected such difference in many other positions (e.g. 159, 223, 248). Our findings support the hypothesis that different HIV-1 subtypes may display different coevolution patterns [86].

### HIV-1 inter-protein coevolution detected by the method combination CNPR

We applied the method combination CNPR to investigate HIV-1 inter-protein coevolution. It is known that the open reading frame of p6 (nucleotides: 120-159) overlaps with protease (nucleotides: 1-40) in the viral genome and that Gag cleavage sites (GCS) interact with protease during the protease-mediated proteolytic processing [4,87]. Since Gag cleavage sites interact with protease residues, mutations near Gag cleavage sites can be selected under the selective pressure of protease inhibitors [4,88]. CNPR predicted Gag cleavage sites 128, 373-375, 431 and 448-453 coevolving with protease residues close to the active site. This is in agreement with previous findings that amino acid substitutions at these Gag cleavage sites are associated with PI resistance [4,88].

In our analysis of p6-protease inter-protein coevolution, 17.9% of the predicted coevolving residues were true positives and 58.9% contained either a Gag or a protease position in HIV-1 clinical and experimental datasets. In the p6-protease overlapping region (Gag position: 487-500, protease position: 1-13. e.g. T4 and L10), many p6 residues (75.7%) were coupled with protease residues (e.g. T4), illustrating the HIV-1 coevolution in the p6-protease overlapping region. Moreover, p6 residues are mostly coupled with the protease position T4 and protease positions (L10, V82, L90) near the protease substrate-binding pocket (Figure 6B). Recognized by the known drug resistance algorithms (e.g. IAS-USA, HIVdb, Rega) [89], all these protease positions are associated with PI drug resistance.

Besides the protease-p6 and protease-GCS coevolution, other inter-protein relationships have been reported between Gag proteins. A recent study showed that the p6 residue S40 can partially rescue the negative effects of capsid mutants at the positions E207, A208 and P231 [90]. Matrix can fold back onto nucleocapsid to regulate Gag assembly by the lateral Gag-Gag inter-protein interaction [91]. While the matrix-nucleocapsid interaction interface remains unclear, residues between the matrix domain (positions: 114-126) were coupled with the nucleocapsid domain (positions: 379-383) in our prediction model. Since the predicted coevolving residues do not necessarily imply the spatial proximity or direct protein-protein interactions [24], structural experiments are still needed to clarify the matrix-nucleocapsid interaction interfaces.

### Limitations and future perspectives

Our ensemble approach has its limitations. (1) ECS assembles individual methods so that combinations of methods cannot reveal coevolving residues that are absent in the predictions of individual methods. (2) For some datasets, the method combination CNPR does not always perform the best compared to individual methods. However, it does provide robust predictions with the highest overall ranking in our performance evaluation (Table 2, Additional file 2: Table S3). (3) It can be computationally expensive to assemble prediction results obtained from multiple methods, especially when phylogeny-based methods are integrated. According to our experience, it usually takes more than 30 hours to test a single dataset using all 27 methods (system settings: Linux, CPU 2.8GHz × 4). High-standard file management is also needed to organize different inputs and outputs for the 27 methods.

Our study aimed at detecting coevolution in different HIV-1 proteins and our performance comparison was restricted to HIV-1 datasets. Our study mainly focused on HIV-1 subtype B because of the limited availability of structural and experimental data for the evaluation of coevolution predictions in other subtypes. As different HIV-1 subtypes may display different coevolution patterns [86], further investigations still need to distinguish coevolving residues in various HIV-1 subtypes. Moreover, we still need to improve the computation efficiency of ECS and to examine the performance of ensemble methods using large-scale protein family datasets. As new sequence-based coevolution methods continue to be reported [22], future studies also need to integrate new methods in the ensemble coevolution system.

## Conclusions

Our study presents a new ensemble coevolution system that integrates multiple sequence-based methods to improve the prediction of HIV-1 position-specific coevolution. Using HIV-1 structural and experimental data, this ensemble system enabled us to identify a combination of 4 different methods that outperformed 27 sequence-based methods for the prediction of HIV-1 inter- and intra-protein coevolution. We also investigated HIV-1 intra- and inter-protein coevolution by exploring coevolving residues in the HIV-1 Gag and protease proteins, which are responsible for viral morphogenesis. Overall, our ensemble coevolution system can detect HIV-1 intra- and inter-protein coevolution, leading to a better understanding of coevolution at the molecular level.

## Reviewers’ comments

### Reviewer report 1

Dr. Zoltán Gáspári (Faculty of Information Technology and Bionics, Pázmány Péter Catholic University, Budapest 1083, Hungary).

The problem discussed in the manuscript is important and original. Finding a reliable set of coevolving residues is a nontrivial task. The approach of combining a number of recently developed methods is promising and it can be expected that it is capable of yielding results superior to any particular singular approach included.

Comment 1: The sequence data set used in the study is selected and curated with care. However, I expected to find some information on the quality of the alignments prepared. In particular, the diversity of the sequences and the number of gaps might be critical for such a coevolutionary study.

*Author’s response: We thank the reviewer for the interesting comment. In response, for each protein sequence file, we calculated the amino acid diversity and the percentages of gaps (see the new* Additional file 2: Table S2*). In the revised manuscript, we added the information about the sequence diversity and the number of gaps as follows:*

*Line 300: “These HIV-1 datasets individually contained more than 200 sequences and the percentage of gaps in each sequence dataset was less than 0.22% (*Additional file 2: Table S2*). In a good agreement with our previous study* [57]*, the amino acid diversity of our sequence datasets was between 4.57% and 14.30% (*Additional file 2: Table S2*)”*.

Additional file 2: Table S2: *Summary of 7 sequence datasets for detecting HIV-1 coevolution*.

Comment 2: The interpretation of the results is generally acceptable, and the shortcomings of the methods and the possible pitfalls in the interpretation are discussed properly. I feel that the types of data discussed are a bit too limited (a single HIV-1 subtype).

*Author’s response: Thanks for this important suggestion. Indeed, it has been reported that genetic variation characterizing different HIV-1 subtypes may confer different coevolving mutation patterns* [86]*. Although a subtype-specific analysis may enrich the understanding of subtype-specific coevolution, our evaluation on coevolution predictions focused on HIV-1 subtype B based on the clinical and experimental data. Firstly, most crystalized HIV-1 protein structures are from the reference strains of HIV-1 subtype B. Secondly, only a few studies have reported coevolving amino acid patterns between protease and Gag proteins for HIV-1 non-B subtypes* [86]*. In contrast, clinical and experimental data of HIV-1 subtype B are sufficiently available in many studies (see* Additional file 2: Table S1*), which provide the efficient evaluation of inter-protein coevolution. In our revision, we discussed the issue of subtype-specific coevolution patterns as follows*.

*Line 563: “Our study mainly focused on HIV-1 subtype B because of the limited availability of structural and experimental data for the evaluation of coevolution predictions in other subtypes. As different HIV-1 subtypes may display different coevolution patterns, further investigations still need to distinguish coevolving residues in various HIV-1 subtypes”*.

Comment 3: The methods are generally appropriate and well-described. The choice of 8 Angstroms for contact distance should be described in more detail, I guess it refers to the closest heavy atom-heavy atom distance in the two residues. As such a choice is always arbitrary (even if it is common in the literature), it is desirable to investigate the dependence of the results on its exact value, e.g. to take into account structural variability (as the authors use single PDB structures for each protein) and possible internal dynamics of the proteins investigated.

*Regarding the contact distance of 8 angstroms, we used the definition of contact residues clarified in literature, which defines the C-beta atoms between two residues* [50]*. As the reviewer noticed, the cutoff of pre-defined contact distance seems to be arbitrary and only indicates the static protein structures rather than the dynamic protein structures. To our knowledge, there is no widely-used measurement that takes into account the structural variability. This process may face difficulties by other limitations such as the shortage of dynamic structural data (e.g. NMR) or protein modeling methods. Note that NMR data is only available for a small amount of protein structures, and protein modeling methods usually require additional parameters that are hypothesized for the simulation purpose.*

*Due to this limitation, our initial analysis evaluated sequence-based methods using four different measurements including: precision-recall curve (AUC), accuracy, harmonic distance and average Euclidean distance (see* Additional file 2: Table S3*). Among them, harmonic distance and Euclidean distance are two measurements that are independent of the cutoff of contact distance. Only AUC and accuracy are related to contact distance of 8 angstroms. More importantly, harmonic distance was originally proposed to replace contact prediction because harmonic distance can evaluate the distribution of Euclidian distances for all predicted coevolving pairs* [50,62]*. We used the contact map for our evaluation purpose because it is still one of the most widely-used standard measurements in the coevolution field, to compare different sequence-based methods* [25,48,50]*. In our comparison, we showed that our ensemble methods are mostly ranked as the top 1 or 2, even for the measurements of harmonic distance and Euclidean distances.*

*We acknowledge however that this concern of both this reviewer and ourselves was not sufficiently made clear in the first submission. To clarify this issue, we added the following sentences to the **Method** section.*

*Line 171: “We used the cutoff of 8 angstroms between C*_*β*_*atoms of residue pairs to detect residue contact, and a strict cutoff of 5 angstroms was also examined”.*

*Line 215: “Note that the harmonic distance does not impose a distance cutoff for the evaluation of coevolution predictions”.*

*Line 224: “Note that the average Euclidean distance does not impose a distance cutoff for the evaluation of coevolution predictions”.*

Comment 4: In the ensemble coevolution method the authors used equal weights according to the supplementary material, so the authors might want to consider to put less emphasis in the description of the weighting, which is obviously a further possibility to explore.

*Author’s response: We thank the reviewer for this suggestion. The weighting strategy was inspired by the weighted voting, where different methods can be weighted according to factors such as computation time and prediction power. The weighting parameters are used to efficiently combine a wide range of different models such as majority voting, Borda count and weighted voting. During our try-and-fail stage, we examined different weighting strategies. Due to the difficulty of parameter optimization using limited number of datasets, our manuscript did not extend into a discussion of the weighting results. Nevertheless, we consider that a comprehensive description of the model is needed, which shields light on weighting optimization when larger training datasets are provided in coming studies. To accommodate for the remark of the reviewer, we moved the description of the weighting strategies to the supplementary text (*Additional file 1: Text S1*).*

*Line 261: “For weighted voting, ranking is done after collecting the weighted votes (see the detailed description in* Additional file 1: Text S1*)”.*

*We moved the following section from the methods section to* Additional file 1: Text S1*:*

*“The weighted votes are collected as follows:*

*Suppose a combination of methods is denoted by *Ω*, *|Ω| *is the number of methods in the method combination *Ω*, and w*_*i*_*is the weight of sequence-based method M*_*i*_*contributed to the coevolution scores. All methods contribute equally when every w*_*i*_*equals to 1. The normalized coevolution scores*${C}_{n,m}^{*}\left(\varOmega, {D}_j\right)$*is defined as:*

$$ {C}_{n,m}^{*}\left(\varOmega, {D}_j\right)=\frac{1}{\left|\varOmega \right|}{\displaystyle \sum_{M_i\in \varOmega }{w}_i\times {C}_{n,m}^{*}\left({M}_i,{D}_j\right)} $$

${C}_{n,m}^{*}\left(\varOmega, {D}_j\right)$*is thereafter ranked and exported as outputs. Notably, *Ω* can either contain a single method or a combination of methods, which can be selected based on the performance evaluation”.*

Comment 4: The strength of the methods used is the inclusion and comparison of a number of different algorithms. Most of the weaknesses are presented properly and are generally applicable to similar coevolutionary studies. The manuscript is supported by rich supplementary data, which is important for the understanding and reproducibility of the results. I especially welcome that the authors provide the MATLAB source code for their ensemble method. The writing and organization of the manuscript is clear, the figures are appropriate and informative, although I think that Figures 3 and 4 could go as supplementary material, especially as the data presented in Figure 3A are closely related to those shown in Table 1. I would suggest that the authors consider the following points for preparing the final version of the manuscript:

*Author’s response: We thank the reviewer for this suggestion. We rearranged *Figure 3*A to* Additional file 1: Figure S1*. As for *Figure 4*, we consider it as a key representation of our clustering analysis which shows that similar sequence-based methods also provide similar predictions. While these findings could be perceived as seemingly trivial, we do believe that our visual representation is conceptually important.*

Comment 5: I would have desired a short outlook with an alternative data set (e.g. a single protein from a wider range of HIV1 subtypes, as mentioned by the authors as a potential source of conflict with results of an independent study) and the discussion of an example where coevolving residues are clearly not in spatial contact.

*Author’s response: Indeed, our discussion mentioned that: “Our study observed different predictions within matrix and capsid, possibly because we focused on HIV-1 subtype B, while the coevolution analysis in* [5] *used a mixed subtype B and C dataset. Further investigation needs to distinguish coevolving residues in HIV-1 subtypes B and C”.*

*We would like to give more details to explain this difference between different HIV-1 subtypes, in addition to our recent publications exploring such differences* [57,86]*. Currently, there are 8 HIV-1 subtypes and more than 60 classified circulating recombinant forms (CRFs) recorded in the Los Alamos National Laboratory (LANL) database (**http://www.hiv.lanl.gov/content/index **). The amino acid sequence diversities of HIV-1 Gag proteins between different subtypes and CRFs are between 15% and 20%* [86]*. More importantly, 103 (20.6%) of 500 Gag amino acid positions have different consensus AAs when 8 different subtypes and CRFs (A1, B, C, D, F1, G, CRF01_AE, CRF02_AG) are compared* [86]*. It is clear that merging sequences from a wide range of HIV-1 subtypes can give quite different amino acid populations in sequence datasets.*

*To further our discussion, let us give a simple example of Capsid in subtype B and C – two most common subtypes in the HIV epidemic. At the amino acid position 280 (Gag protein index), the prevalence of amino acids T (68.8%) and V (22.1%) in subtype B is clearly different from two most common amino acids T (1.8%) and V (97.9%) in subtype C* [86]*. Using the alternative sequence datasets of subtype B and C from* [86]*, we performed coevolution analysis and found that position 280 coevolved with many positions in subtype B (e.g. positions 138, 146, 147) but not in subtype C. As a possible explanation to this observation, position 280 is much more conserved in subtype C than subtype B. With less than 2% amino acid variation at position 280, sequence-based methods can hardly produce strong signals in subtype C. Moreover, a plenty number of such positions have been observed between subtype B and C (e.g. positions 159, 223, 248, 260). We added an example in our discussion to clarify this observation.*

*Line 508: “Our previous study showed a high amino acid diversity of Gag (18.38%) between subtypes B and C* [57]*, which may lead to different coevolution predictions in sequence-based analyses* [86]*. Using the alternative sequence datasets of subtypes B and C from* [57]*, position 280 in Capsid was predicted by CNPR to coevolve with many positions (e.g. 138, 146, 147) in subtype B, but not in subtype C. Note that at amino acid position 280 (Gag index), the prevalence of amino acids T (68.8%) and V (22.1%) in subtype B is clearly different from two most common amino acids T (1.8%) and V (97.9%) in subtype C* [57]*. This indicates that position 280 is much more conserved in subtype C than subtype B*, *thus the power to detect a significant signal is lower in subtype C. Besides position 280, we also detected such difference in many other positions (e.g. 159, 223, 248). Our findings support the hypothesis that different HIV-1 subtypes may display different coevolution patterns* [86]*”*.

Comment 6: The alignments themselves could be provided as supplementary material allowing for straightforward reproduction of the data; they could also be used as a test data set for prospective users.

*Author’s response: We thank the reviewer for the suggestion. We have made the aligned sequence datasets available as supplementary materials in *Additional file 3*.*

Comment 7: The authors might want to rerun the evaluation by using different contact thresholds in the structures: values of e.g. 7 and 9 Angstrom could be tested and evaluated as a verification of the robustness of the method.

*Author’s response: As discussed in Comment 3, harmonic distance and average Euclidean distance are two of our four evaluation measurements which provide robust evaluation of sequence-based methods and both are independent of any contact thresholds. We chose the cutoff of contact distances based on the empirical data in literature* [25,48,50]*. Actually, the 5 and 8 angstroms are the most common cutoffs to determine the Euclidean distance between two contact residues* [25,92,93]*. Besides our previous results using the threshold of 8 angstroms, we performed new evaluations using the threshold of 5 angstroms. Our results showed that our ensemble methods are still ranked as the top sequence-based methods (see* Additional file 2: Table S8*). We added this result in our revision.*

*Line 448: “While our evaluations of the predictive performance mainly used 8 angstroms as the threshold of contact distance, our method CNPR also achieved top rankings when a strict cutoff value of 5 angstroms was applied (*Additional file 2: Table S8*)”.*

Minor issues not for publication:

Comment 1: I would refrain from using the word “fullerene core” as it might be confusing (refers to a geometric similarity but has nothing to do with fullerene molecules).

*Author’s response: We thank the reviewer for this suggestion. Actually, “fullerene core” has been commonly used to describe the shape of viral core in the HIV field* [1,94]*, but we acknowledge the possible confusion. In the revision, we replaced “fullerene core” with “viral core”.*

Comment 2: The authors seem to use the word “domain” instead of “subunit” to refer to protein chains in multimeric structures, this should be corrected/clarified before final publication.

*We thank the reviewer for this suggestion. In line with the reviewer, we have used “subunit” to replace “domain” at the respective sections of our revision.*

## Additional files

Additional file 1: Text S1. Description of our ensemble algorithm (section 1) and summary of the 27 sequence-based methods published between 2004 and 2013 (section 2). Additional file 2: Figure S1. Evaluation of the method combination CNPR and the 27 individual methods applied to the 7 HIV-1 datasets. **Figure S2.** Jaccard and association coefficients between CNPR and 27 sequence-based methods. **Figure S3.** HIV-1 nucleocapsid coevolving pairs predicted by CNPR. **Figure S4.** Contact map of HIV-1 protease and coevolving pairs predicted by 28 sequence-based methods. **Figure S5.** Contact map of HIV-1 matrix and coevolving pairs predicted by 28 sequence-based methods. **Figure S6.** Contact map of HIV-1 capsid and coevolving pairs predicted by 28 sequence-based methods. **Figure S7.** Contact map of HIV-1 nucleocapsid and coevolving pairs predicted by 28 sequence-based methods. **Table S1.** Summary of PI-associated Gag and protease substitutions reported in the experimental or clinical studies. **Table S2.** Summary of 7 sequence datasets for detecting HIV-1 coevolution. **Table S3.** Ranking of sequence-based methods using individual HIV-1 datasets. **Table S4.** Accuracy of sequence-based methods on individual HIV-1 datasets (threshold of contact distance: 8 angstroms). **Table S5.** Harmonic distance of sequence-based methods using individual HIV-1 datasets (Å). **Table S6.** Average Euclidean distance (Å) of the top-ranked long-range couplings predicted by sequence-based methods. **Table S7.** Summary of long-range residue contacts derived from HIV-1 Gag and protease protein structures. **Table S8.** Accuracy of sequence-based methods on individual HIV-1 datasets (threshold of contact distance: 5 angstroms). Additional file 3:
**Software.** The Matlab toolbox and the manual of our ensemble coevolution system are provided. The sequence datasets are also available.

## Abbreviations

AA: Amino acids
AUC: The area under the precision-recall curve
CA: Capsid
CASP: Critical assessment of protein structure prediction
CTD: C-terminal domain
CRF: Circulating recombinant form
CNPR: a method integrating four sequence-based methods (CMPro, NCPS, PhyCMAP, RCW)
ECS: Ensemble coevolution system
GCS: Gag cleavage site
GTR: General time reversible model
HLA: Human leukocyte antigen
MA: Matrix
MSA: Multiple sequence alignment
NTD: N-terminal domain
NC: Nucleocapsid
PR: Protease
PI: Protease inhibitor
