# Supplementary material for: A new ensemble coevolution system for detecting HIV-1 protein coevolution
Source: Biol Direct. 2015 Jan 7;10:1. doi: 10.1186/s13062-014-0031-8 (PMC4332441; doi:10.1186/s13062-014-0031-8)
Supplement: Additional file 1: Text S1. — Description of our ensemble algorithm (section 1) and summary of the 27 sequence-based methods published between 2004 and 2013 (section 2). [file 13062_2014_31_MOESM1_ESM.pdf]

Supplement text S1: A new ensemble coevolution system for detecting HIV-1 protein coevolution

Guangdi Li[1,*], Kristof Theys[1], Jens Verheyen[2], Andrea-Clemencia Pineda-Peña[1,3], Ricardo Khouri[1], Supinya Piampongsant[1], Mónica Eusébio[4], Jan Ramon[5], Anne-Mieke Vandamme[1,4,*]

1 Clinical and Epidemiological Virology, Rega Institute for Medical Research, Department of Microbiology and Immunology, KU Leuven - University of Leuven, Leuven, Belgium

2 Institute of Virology, University hospital, University Duisburg-Essen, Essen, Germany

3 Clinical and Molecular Infectious Diseases Group, Faculty of Sciences and Mathematics, Universidad del Rosario, Bogotá, Colombia

4 Centro de Malária e outras Doenças Tropicais and Unidade de Microbiologia, Instituto de Higiene e Medicina Tropical, Universidade Nova de Lisboa, Portugal

5 Department of Computer Science, KU Leuven - University of Leuven, Leuven, Belgium

∗ **E-mail: liguangdi.research@gmail.com, annemie.vandamme@uzleuven.be**

Contents

1 Ensemble coevolution system **2**

2 Position-specific sequence-based methods in the last decade **6**

2.1 MI: mutual information [1] 7
2.2 ASC and APC: average sum and product correction [2] (2008) 8
2.3 RCW: row and column weighted MI [3] (2007) 9
2.4 ZRES: Z-residue score [4] (2009) 10
2.5 Interdependency V2.1 [5] (2004) 10
2.6 NBZPX2: normal binary ZPX2 [6] (2012) 11
2.7 MIBP: MI with physicochemical property [7] (2011) 12
2.8 PhysicoMI: physico-chemical corrected MI [8] (2012) 13
2.9 NCPS: normalized coevolutionary pattern similarity [9] (2009) 13
2.10 SCA: statistical coupling analysis [10] (2009) 14
2.11 Complementary: complementary matrix in Pearson coefficient [11] (2006) . . . 16
2.12 PCC: Pearson correlation coefficient [12] (2010) 17
2.13 LogR: disentangling direct coupling analysis [13] (2010) 17

2.14 DCA: direct coupling analysis [14] (2011) 19
2.15 PSICOV: precise structural contact prediction [15] (2012) 20
2.16 SVMcon: support vector machine contact map predictor [16] (2007) 21
2.17 NNcon: neural network-based contact map predictor [17] (2009) 21
2.18 DNcon: neural network contact prediction [18] (2012) 22
2.19 CMPro: 2D recursive neural network [19] (2012) 23
2.20 PhyCMAP: random forest, integer linear programming [20] (2013) 23
2.21 Mutagenetic: mutagenetic tree mixture model [21] (2005) 24
2.22 BN: Bayesian network [22] (2007) 25
2.23 Spidermonkey [23] (2008) 26
2.24 CTMP: continuous time Markov process [24] (2007) 27
2.25 CoMap [25] (2011) 28
2.26 GREMLIN: generative regularized models of proteins [26] (2013) 29

3 Implementation and software manual **30**

1 Ensemble coevolution system

Introduction We designed an ensemble coevolution system (ECS) to provide robust predictions of coevolving residues. Ensemble learning systems which combine different prediction methods have shown high prediction performance in many studies (see review in [27, 28]). For instance, XCS was made to improve the self-adaptation of evolutionary algorithms [29]. LCSE enhances the rule-based classification through the combination of reinforcement learning, evolutionary computing and heuristic approaches [30]. GAssist improves the performance of the ordinal classification through the assembling of several rule-based models [31]. A cascade generalization framework which combines naïve Bayesian classifiers, linear discriminant classifiers and decision trees could improve the classification accuracy compared to individual classifiers [32]. An ensemble system has recently been developed to improve the prediction of protein-protein interactions using the attributes collected mainly from gene ontology annotations [33]. This ensemble system integrates four machine learning methods (support vector machine, random forest, decision tree and naïve Bayesian network) based on the majority voting strategy [33]. Recently, an ensemble method which combines PSICOV and machine learning classifiers can improve the prediction of transmembrane inter-helix contacts [34].

Supplementary text S1: Ensemble coevolution system

An ensemble learning system is usually built to combine a set of prediction models and is popular when the prediction variability between prediction models (classifiers) is high [28]. Ensemble learning is not needed if all models predict the same results [28]. Ensemble learning systems usually comprise of three parts: (1) data sampling/selection, (2) model prediction, (3) a combiner. The combiner plays a key role to determine the strategy of how different predictions from various methods are integrated. There are many popular ensemble strategies such as: majority voting (predictions supported by more than 50% of methods), weighted voting (predictions are weighted according to the importance of models) and Borda count (predictions consistently obtained by all the models) [27, 28].

Inspired by the principle of ensemble learning, we endeavored to build a software system which integrates known sequence-based methods for coevolution prediction. It turned out to be difficult for several reasons. Firstly, there is a lack of gold standard datasets for training the ensemble learning system. Secondly, sequence-based methods predict different scores for statistical couplings and mostly do not predict true negatives, which limit our choices on data sampling and ensemble strategies. Thirdly, some sequence-based methods require heavy computational time, a limit which may restrain a broad application of ensemble learning on large protein families. For the above reasons, the potential ensemble coevolution system should provide robust predictions while combining the number of sequence-based methods as few as possible. Moreover, we considered the design of an ensemble coevolution system as an optimization problem, where the objective function was defined as a linear function (see Methods). Here, we describe the details of our heuristic algorithm which we designed to identify the combination of sequence-based methods which improves the prediction performance.

Methodology $M = \{M_i | i = 1, ..., N\}$ represents a set of sequence-based methods M_i and $D = \{D_j | j = 1, ..., T\}$ represents sequence datasets, where N is the number of methods and T is the number of sequence datasets. Given a training dataset D_j, the sequence-based method M_i predicts a coevoltuion score for each statistical coupling. This coevolution score is normalized in the matrix with $L \times L$ elements, denoted as $C^*(M_i, D_j)$, where L is the length of residue positions in D_j. Every non-empty element in this matrix represents the coevolution score of the statistical coupling between the positions n and m. To contend with varied statistical measurements used by different methods, four normalization strategies have been previously proposed [13]. Given a continuous variable x as an input, the normalized variable y satisfies:
(1) Linear transformation: $y = \frac{x - min(x)}{max(x) - min(x)}$.

(2) Power transformation: $y = 10^{(max(x)-x)(max(x)-min(x))}$.
(3) Binary transformation: if $Rank(n, m) < \alpha$, then $y = 1$; otherwise $y = 0$. The cutoff α is the number of top-ranked couplings ($\alpha = L$ in our analysis).
(4) Log transformation: $y = b \times x^a$ where $V_{max} = log_{10}[max(x)]$, $V_{min} = log_{10}[min(x)]$, if $min(x), max(x) > 0$, then $a = K/(V_{max} - V_{min}), b = -K \cdot V_{max}/(V_{max} - V_{min})$, and $K = 5$ as default.

We compared these four strategies and chose linear transformation as our normalization s-trategy because linear transformation performed the best using HIV-1 datasets (data not shown).

Suppose a combination of methods is denoted by Ω, $|\Omega|$ is the number of methods in the method combination Ω, and w_i is the weight of sequence-based method M_i contributed to the coevolution scores. All methods contribute equally when every w_i equals to 1. The normalized coevolution scores $C^*_{n,m}(\Omega, D_j)$ is defined as:

$$C^*_{n,m}(\Omega, D_j) = \frac{1}{|\Omega|} \sum_{M_i \in \Omega} w_i \times C^*_{n,m}(M_i, D_j) \tag{1}$$

$C^*_{n,m}(\Omega, D_j)$ is therefore ranked and exported as outputs. Notably, Ω can either contain a single method or a combination of methods, which can be selected based on the performance evaluation. Based on the normalized coevolution scores, the coupling (n, m) in the entire matrix is ranked, denoted as $Rank(n, m)$. Given a statistical measurement f (e.g. AUC), the performance of the sequence-based method M_i is measured by $f(C^*(M_i, D_j))$. Suppose w_i and u_j denotes the weight of the method M_i and the weight of the D_j, respectively. The objective function for the ensemble learning is defined as:

$$F(\Omega, D) = \sum_{j=1}^{T} \frac{u_j}{|\Omega|} \sum_{M_i \in \Omega} w_i \times f(C^*_{n,m}(M_i, D_j)) \tag{2}$$

An optimized combination of methods Ω^+ is identified when $\Omega^+ = \max_{\Omega \in M} F(\Omega, D)$. To simplify the learning procedure with a small number of training datasets, our study assumed that $u_j = 1$ and w_i equals to 1 or 0. We therefore designed a heuristic algorithm (**Algorithm 1**) that provides a suboptimal solution to identify Ω^+ by maximizing the performance scores. Specifically, we used the forward selection to improve the prediction performance because of the high computational complexity. The forward selection each time adds one method into the optimized method set if the added method increases the performance score. Our heuristic

Supplementary text S1: Ensemble coevolution system

Algorithm 1 A heuristic algorithm for identifying the combination of sequence-based methods

Require: Inputs: a set of sequence-based methods $M = \{M_i | i = 1, ..., N\}$ and multiple sequence datasets $D = \{D_i | i = 1, ..., T\}$.
Ensure: Output: the optimized method combination Ω^+.

$\Omega = \phi$; {Initiate the method set Ω as empty.}
$f(\Omega, D) = 0$; {Initiate the performance score of Ω as 0 given the datasets D.}
$\Delta_i = 0, i = 0, ..., N$; {Initiate the performance increase as 0 for each method.}
Step 1: Apply sequence-based methods and perform the linear transformation.
for $i = 1$ to N **do**
  for $j = 1$ to T **do**
    Obtain the coevolution scoring matrix $C(M_i, D_j)$;
    Linear transformation: $C^*(M_i, D_j) = \frac{C(M_i,D_j) - min(C(M_i,D_j))}{max(C(M_i,D_j)) - min(C(M_i,D_j))}$;
  end for
end for
Step 2: Optimization of method combination.
while $\Omega = \phi$ or $max(\Delta) > 0$ **do**
  for $i = 1$ to N **do**
    $\Omega^* = \Omega \cup M_i$ where $M_i \in \{M - \Omega\}$; {Add the unvisited method M_i to Ω^*.}
    for $j = 1$ to T **do**
      $C^*(\Omega^*, D_j) = \sum_{M_k \in \Omega^*} w_{k,j} \cdot C^*(M_k, D_j)$; {Integrate the coevolution predictions.}
    end for
    $\Delta_i = \frac{1}{T} \sum_{j=1}^{T} f(C(\Omega^*, D_j)) - f(\Omega, D)$; {Increase of average performance score.}
  end for
  if $max(\Delta) > 0$ **then**
    $M^* = arg_{M_i} max(\Delta)$; {A method with the highest increase of performance score.}
    $\Omega = \Omega \cup M^*$; {Update the best method subset.}
    $f(\Omega, D) = f(\Omega, D) + max(\Delta)$; {Update the best performance score.}
  end if
end while
Return Ω as Ω^+.

algorithm begins with the initiation of global variables (line 1-3). The coupling predictions of sequence-based methods M are performed given the sequence dataset D (line 5-10, $T = 7, N = 27$ in our analysis). Given each method with a sequence dataset, statistical couplings in the scoring matrix $C(M_i, D_j)$ are obtained according to method measurements (line 7). The scores are then linearly transformed between 0 and 1 (line 8). Thereafter, the forward selection adds one method into the method subset Ω at each loop (line 14). It also assembles the statistical coupling predictions for the AUC evaluation (line 16, see AUC definition in Methods). The increase of performance score Δ_i is calculated for each method M_i when added into the method subset Ω (line 18). In each round, one method that increases the highest performance score is added into the method subset Ω (line 20-24). The procedure terminates when adding any method does not improve the best performance score (line 12). The method subset Ω is returned as the optimized method combination Ω^+ (line 26).

The forward selection algorithm can be easily adapted into the backward elimination, which requires the initiate parameter $\Omega = \{1, ..., N\}$ and line 14 in the algorithm should remove a s-ingle method instead of adding a method. In order to achieve a promising optimization, we implemented both forward selection and backward elimination approaches. In our experiments, we found that both strategies identified the method set Ω with four methods (NCPS, RCW, PhyCMAP, CMPro), suggesting a convergence of the heuristic search. Overall, our heuristic algorithm offers a fast computation to identify a method combination with improved prediction performance using a local optimization procedure.

Our parameter settings: Parameters of sequence-based methods were initialized according to individual methods (see parameter settings in the next section).

Software availability Our toolbox.

2 Position-specific sequence-based methods in the last decade

This section provides more details about the 27 sequence-based methods (Table 1). For each method, we begin with a simple introduction and then briefly describe their key mathematical models. Lastly, we explain the parameter settings used in this study and the software availability. We order these methods according to their methodology so that methods with similar methodologies are described together.

Table 1. Summary of 27 sequence-based methods integrated in ECS

Method	Methodology	Software availability	Year	Ref
ASC	Mutual information	Our toolbox	2011	[10]
APC	Mutual information	Our toolbox	2011	[10]
BN	Bayesian network	https://code.google.com/p/bright/	2007	[22]
CTMP	Markov model, phylogenetic tree	http://www.stat.sinica.edu.tw/chyeang/	2007	[24]
CoMap	Compensation coefficient, phylogenetic tree	http://gna.org/projects/comap	2007	[25]
Complementary	Complementary matrix, Pearson coefficient	Our toolbox	2006	[11]
CMPro	Neural network	http://scratch.proteomics.ics.uci.edu/	2012	[19]
DNcon	Deep network, Bolzmann machine	http://iris.rnet.missouri.edu/dncon/	2012	[18]
GREMLIN	Maximum entropy function	http://openseq.org/	2013	[26]
Interdependency	Entropy, mutual information	http://www.uhnresearch.ca/labs/tillier/depend2/dependency.html	2004	[5]
LogR	Bayesian network, APC	Author's generosity	2010	[13]
MI	Mutual information	Our toolbox	-	[1]
MIBP	MI, physicochemical property	http://www.biomedcentral.com/1471-2105/12/206	2011	[7]
Mutagenetic	Maximum likelihood mixed tree	http://mtreemix.bioinf.mpi-inf.mpg.de/	2005	[21]
NBZPX2	Normal binary	Toolbox in [6]	2012	[6]
NCPS	Mutual information, sequence similarity	Our toolbox	2009	[9]
NNcon	Neural network	http://casp.rnet.missouri.edu/nncon.html	2009	[17]
PCC	Mutual information, Pearson coefficient	Our toolbox	2010	[12]
PhyCMAP	Random forest, integer linear programming	http://raptorx.uchicago.edu/	2013	[20]
plmDCA	Maximum entropy function	http://plmdca.csc.kth.se/	2013	[35]
PSICOV	Sparse inverse covariance	http://bioinfadmin.cs.ucl.ac.uk/downloads/PSICOV/	2012	[15]
PhysicoMI	MI, AA substitution matrix	Our toolbox	2012	[8]
RCW	Mutual information	Our toolbox	2007	[3]
SCA	Statistical free energy coupling	http://systems.swmed.edu/rr_lab/sca.html	2009	[10]
Spidermonkey	MCMC Bayesian network, phylogenetic tree	http://www.hyphy.org/w/index.php/Main_Page	2008	[23]
SVMcon	Support vector machine	http://casp.rnet.missouri.edu/svmcon.html	2006	[16]
ZRES	Mutual information	Toolbox in [6]	2009	[4]

2.1 MI: mutual information [1]

Introduction Mutual information (MI) measures the contribution of the knowledge of variable X's information in the redution of the uncertainty of the other variable Y [36]. For its simplicity, MI has been adapted to predict coevolving positions and protein contact map [37]. It was proposed based on the hypothesis that coevolving residues or residues in contact tend to share a high mutual information [1].

Methodology Let variables X, Y represent two protein positions in multiple sequence alignment (MSA) from a protein (family), $X = x$ indicates that the position X takes the amino acid x which is one of amino acid forms in MSA, $P(X = x)$ represents the marginal probability of

the position X taking the amino acid form x in MSA. Likewise, $P(X = x, Y = y)$ is the joint probability that the position X takes the amino acid form x and Y takes the amino acid form y simultaneously. The mutual information between $X = x$ and $Y = y$ is defined as:

$$MI(X = x, Y = y) = P(X = x, Y = y) \log \frac{P(X = x, Y = y)}{P(X = x)P(Y = y)} \tag{3}$$

Furthermore, the mutual information between two positions X, Y is the sum of mutual information of all possible configurations at the position X and Y, defined as:

$$MI(X, Y) = \sum_x \sum_y P(X = x, Y = y) \log \frac{P(X = x, Y = y)}{P(X = x)P(Y = y)} \tag{4}$$

Our parameter settings In cases where the marginal probability $P(X = x)$ or $P(Y = y)$ in the denominator was zero causing the impossible infinity in the log function of MI, Laplace smoothing was used by adding 1 into the both denominator and the numerator of the marginal probability $P(X)$ ($P(X = x) = n/N \rightarrow P(X = x) = (n + 1)/(1 + N)$) [38]. Gaps from the positions of interest were ignored in the calculation of probabilities [1]. To solve the heavy computation in large protein families, a fast parallel programming code is provided in our Toolbox.

Software availability Our toolbox and toolbox in [6].

2.2 ASC and APC: average sum and product correction [2] (2008)

Introduction In this study, two statistical methods were proposed to estimate the background MI in the process of protein evolution. The background MI was defined as the average MI over all position pairs [2]. The average sum correction (ASC) was defined as the sum of MI calculated at the positions i and j minus the background MI [2]. Similarly, the average product correction (APC) was defined as the product of MI calculated calculated at the positions i and j divided by the background MI.

Methodology Based on mutual information described in the previous section, the background

mutual information in ASC [2] is defined as:

$$ASC_{Backgroud}(i,j) = \frac{1}{2n}\sum_{X}[MI(i,X) + MI(X,j)] - \frac{1}{n^2}\sum_{X}\sum_{Y\neq X} MI(X,Y) \tag{5}$$

Suppose i and j are two residue positions of interest and n is the number of the overall protein positions. Given an input MSA, the second part of above formula is a constant so that the ASC correction for mutual information is defined as:

$$ASC_{MI}(i,j) = MI(i,j) - \frac{1}{2n}\sum_{X}[MI(i,X) + MI(X,j)] \tag{6}$$

Assuming that the background dependency is a product of independent factors associated with two positions, the average product correction (APC) is defined as:

$$APC_{MI}(i,j) = MI(i,j) - \frac{\sum_{X} MI(i,X) \times \sum_{Y} MI(Y,j)}{\sum_{X,Y} MI(X,Y)} \tag{7}$$

Parameter settings We used the same parameter settings as the mutual information.

Software availability Our toolbox.

2.3 RCW: row and column weighted MI [3] (2007)

Introduction Similar to the methodology of ASC and APC, the method RCW takes the average mutual information as the ”weight” for the pairwise dependency. Given simulated datasets, RCW outperformed MI, logarithm correlation and multi-dimensional amino acid representation [3].

Methodology Let i and j be the positions of interest, $MI(i,j)$ denotes the mutual information between the positions i and j. The mean value of mutual information at the position i is calculated as $\overline{MI_i} = \sum_{j=1}^{n} MI(i,j)/(n-1)$, RCW between the position pair (i,j) is defined as:

$$RCW(i,j) = \frac{MI(i,j)}{\overline{MI_i} + \overline{MI_j} - 2MI(i,j)/(n-1)} \tag{8}$$

Our parameter settings We used the same parameter settings as those in mutual information.

Software availability `http://www.cbs.dtu.dk/services/InterMap3D/`.

2.4 ZRES: Z-residue score [4] (2009)

Introduction This study provided a method which refines MI by removing strong non-coevolutionary influence and accounting for the position variability. The method is built based on the linear regression between the mutual information MI_{ij} and $\overline{MI_i} \times \overline{MI_j}$. Using protein sequences from 1592 protein families in the Pfam database (http://pfam.sanger.ac.uk/), this study showed that predicted coevolving positions tend to be in a close physical proximity [4].

Methodology As the stochastic and phylogenetic bias may affect the performance of MI, ZRES uses the linear regression to fit the mutual information MI_{ij} with $\overline{MI_i} \times \overline{MI_j}$. By doing so, the biases can be measured by $Res_{ij} = \overline{MI_i} \times \overline{MI_j} - \beta \cdot MI_{ij}$, where β is the estimated coefficient in the linear regression. Based on this principle, the statistical coupling between the positions i and j is quantified by the Z-score, denoted as $ZRes(i,j)$:

$$ZRes(i,j) = \frac{(Res_{ij} - \frac{1}{n}\sum_{j=1}^{n} Res_{ij})(Res_{ij} - \frac{1}{n}\sum_{i=1}^{n} Res_{ij})}{\sqrt{\sum_{i=1}^{n}(Res_{ij} - \frac{1}{n}\sum_{j=1}^{n} Res_{ij})^2}\sqrt{\sum_{j=1}^{n}(Res_{ij} - \frac{1}{n}\sum_{i=1}^{n} Res_{ij})^2}} \tag{9}$$

The higher the Z-score $ZRes(i,j)$, the higher the chance that two positions i and j are coevolving.

Our parameter settings We used the default parameter settings in the ZRES toolbox [6].

Software availability Toolbox in [6].

2.5 Interdependency V2.1 [5] (2004)

Introduction To reduce phylogenetic bias, the statistical couplings in this method were quantified by the statistical interdependency ratio, which measured the differences between the ob-

served residue interdependency and the expected residue interdependency [5].

Methodology The expected independency is estimated by the likelihood of equivalent residues compared to all residues at other positions. Let N be the number of non-gap residues at the position X_i, the expected interdependency of the position X_i is defined as:

$$MS(X_i) = \frac{1}{N} \sum_{j \neq i} \sum_{x_i, x_j} \log \frac{P(X_i = x_i, X_j = x_j)}{P(X_i = x_i) P(X_j = x_j)} \tag{10}$$

The interdependency is proposed using the entropy weight based on the hypothesis that residue positions do not increase the dependency when no functional correlation exists [5]. To quantify the amino acid variation at the position X_i, the entropy function $H(X_i)$ is measured through $H(X_i) = -\sum_{x_i} P(X_i = x_i) \log P(X_i = x_i)$. Based on the mutual information and the entropy function, the interdependency ratio is defined as:

$$R(X_i, X_j) = \frac{MI(X_i, X_j) H(X_i) H(X_j)}{MS(X_i) + MS(X_j)} [1 - H(X_i) H(X_j)] \tag{11}$$

Our parameter settings We performed the analyses using the default statistical parameters.
Software availability `http://www.uhnresearch.ca/labs/tillier/depend2/dependency.html`.

2.6 NBZPX2: normal binary ZPX2 [6] (2012)

Introduction The method NBZPX2 which is an adaption of ZRES improves the performance of ZRES by refining the MSA inputs. The refinement strategy uses the data transformation called the normal binary [6].

Methodology The workflow of this method can be simply described by three steps. (1) The sequences in the MSA inputs are reordered using the sequence similarity, i.e. the 1st and 2nd sequences are the most similar sequences and the 3rd sequence is the the closest one to the 2nd, etc. This reordering process terminates until all sequences have been visited. (2) The MSA input is transformed into a binary dataset through AA comparisons of two subsequent sequences. Specifically, the binary value 1 indicates that two AAs are identical, otherwise 0. (3) The ZRES algorithm is applied using the transformed dataset.

Our parameter settings We used the default parameter settings.

Software availability Toolbox in [6].

2.7 MIBP: MI with physicochemical property [7] (2011)

Introduction This study proposed a covariation model which uses mutual information accounting for residue physicochemical properties [7]. Ten groups of amino acids were classified according to physicochemical properties. The key concept of this model is to calculate the mutual information between amino acid groups, while taking into account the background amino acid distribution. Performance of this model was compared to MI and ELSC using three protein families (1JXA-A, 1B93-A and PF01053).

Methodology Based on MI, the MIBP covariation between the positions i and j is defined as:

$$MIBP(i,j) = \sum_{a_n}\sum_{b_m} P(x_i \in a_n, x_j \in b_m) \log \frac{P(x_i \in a_n, x_j \in b_m)}{P_b(x_i \in a_n) P_b(x_j \in b_m)}$$

Where x_i and x_j are residues at the positions i and j in the MSA input, a_n (n=1...10) and b_m (m=1...10) denotes the AA functional group at the positions i and j, respectively. Ten residue groups include hydrophobic (A, G, C, T, I, V, L, K, H, F, Y, W, M), aromatic (F, Y, W, H), aliphatic (I, V, L), tiny (A, S, G, C), small (P, N, D, T, C, A, G, S, V), proline (P), charged (K, H, R, D, E), negative (D, E), polar (N, Q, S, D, E, C, T, K, R, H, Y, W) and positive (K, H, R). Based on BLOSUM62 substitution matrix, $P_b(x_i \in a_n)$ is the background distribution of physicochemical properties and is defined as:

$$P_b(x_i \in a_n) = \frac{P(x_i \in a_n)/B(a_n)}{\sum_{a_n} P(x_i \in a_n)/B(a_n)}$$

Where $B(a_n)$ denotes the BLOSUM62 constants for functional groups (hydrophobic: 0.504, aromatic: 0.132, aliphatic: 0.111, tiny: 0.243, small: 0.6632, charged: 0.226, negative: 0.117, positive: 0.507, proline: 0.244, polar: 0.043) [7].

Our parameter settings We used the default parameters in the original python implementation.

Software availability `http://www.biomedcentral.com/1471-2105/12/206`.

2.8 PhysicoMI: physico-chemical corrected MI [8] (2012)

Introduction PhysicoMI was proposed to calculate residue similarities taking into account physical-chemical properties. In this method, MI and AA frequency are corrected using the BLOSUM62 substitution matrix [8].

Methodology Suppose x, y are two AAs at the positions X and Y respectively, the corrected joint probability of X in the presence of Y is modeled as:

$$f(X = x, Y = y) = \frac{n(x, y) + \sum_{(x',y')\neq(x,y)} n(x', y')S(x, x')S(y, y')/\sqrt{N}}{N + \sqrt{N}} \tag{12}$$

Where $n(x, y)$ is the number of residues x at the position X and y at the position Y. $S(x, x')$ is the substitution score when amino acid x' is replaced by x. In the same fashion, the corrected marginal probability is defined as:

$$f(X = x) = \frac{n(x) + \sum_{x'\neq x} n(x')S(x, x')/\sqrt{N}}{N + \sqrt{N}} \tag{13}$$

In the final measurement, both marginal and joint probabilities are corrected by physical-chemical properties:

$$MI_{Physico}(X, Y) = \sum_{X=x}\sum_{Y=y} f(x, y) \log \frac{f(X = x, Y = y)}{f(X = x)f(Y = y)} \tag{14}$$

Our parameter settings We used the default parameter settings.

Software availability Our toolbox.

2.9 NCPS: normalized coevolutionary pattern similarity [9] (2009)

Introduction To identify coevolving positions by MI can be complicated due to common ancestry and stochastic noise [9]. NCPS was therefore proposed to normalize sequence similarities

by reducing the background noise in the correlated mutation analysis. This study showed that the background noise could be reduced using three coevolution analyses: McBASC, OMES and MI [9].

Methodology Suppose $CM(i, j)$ represents the correlated mutation score between the positions i and j. The coevolutionary pattern similarity (CPS) between the positions i and j is modeled by the dot product of two vectors. Let n be the number of overall residue positions in the sequences, $CPS(i, j)$ is defined as:

$$CPS(i,j) = \frac{1}{n-2} \sum_{k \neq i,j} CM(i,k)CM(j,k) \tag{15}$$

Secondly, the coevolutionary pattern similarity is normalized as follows:

$$NCPS(i,j) = \frac{CPS(i,j)}{\sqrt{\frac{1}{n(n-1)} \sum_{i,j} CPS(i,j)}} \tag{16}$$

Thirdly, the NCPS score is proposed by removing the background noise.

$$aMIc(i,j) = \frac{1}{2} \left[\frac{MI(i,j) - NCPS(i,j)}{\max[MI(i,j) - NCPS(i,j)]} + \frac{E(i,j)[MI(i,j) - NCPS(i,j)]}{\max\left[E(i,j)[MI(i,j) - NCPS(i,j)]\right]} \right] \tag{17}$$

Where $E(i, j) = H(i)H(j)[1 - H(i)H(j)]$ is the entropic factor [9].

Our parameter settings We used the default parameter settings.

Software availability Our toolbox.

2.10 SCA: statistical coupling analysis [10] (2009)

Introduction Statistical coupling analysis (SCA) has been shown to reveal allosteric communications by the energetically coupled positions in the PDZ protein family [39]. Later studies showed that SCA could discover evolutionary networks which mediate the allosteric communications [40]. Moreover, SCA can be useful for protein design. For instance, artificial WW

domains were designed based on the statistical couplings predicted by SCA. The artificial WW domains can be folded and bind with peptides in high affinities as natural WW domains [41].

Several follow-up studies have been devoted to improve the performance of SCA using different protein families [10,42,43]. The SCA toolbox written in Matlab® has been updated to Version 5.0, including different functions such as coupling prediction, independent component analysis and spectral decomposition. The latest version of SCA is available online.

Methodology SCA measures the statistical couplings observed in the functional interactions of protein families [39]. The hypothesis relies on the observation that the distribution of amino acids in one position shifts due to the changes of amino acid distribution at another position. The degree of evolutionary dependence is quantified by the statistical coupling energy based on the Boltzmann equation [39]. The high coupling energy corresponds to the increased dependence between coevolving residues. Specifically, the statistical coupling energy between the positions i and j, denoted as $\Delta\Delta G_{i,j}$, is modeled as:

$$\Delta\Delta G_{i,j} = \sqrt{\sum_x (\ln P^x_{i|\delta j} - \ln P^x_i)^2} \tag{18}$$

Where P^x_i is the probability of the residue x at the position i; $P^x_{i|\delta j}$ is the probability of the residue x at the position i given the perturbation position δj. The method in SCA V5.0 extends the coevolution estimation by the covariance analysis and principle component analysis [42]. Briefly, the pairwise correlation $C^{(ab)}_{ij}$ between the residue a at the position i and the residue b at the position j is modeled as:

$$C^{(ab)}_{ij} = \ln\left[\frac{f^{(a)}_i(1-q^{(a)})}{(1-f^{(a)}_i)q^{(a)}}\right](f^{(ab)}_{ij} - f^{(a)}_i f^{(b)}_j)\ln\left[\frac{f^{(b)}_j(1-q^{(b)})}{(1-f^{(b)}_j)q^{(b)}}\right] \tag{19}$$

Where $f^{(a)}_i$ is the frequency of having the residue a at the position i, $f^{(ab)}_{ij}$ is the joint frequency of having the residue a at the position i and the residue b at the position j, $q^{(a)}$ is the background probability of residue a in all proteins and q = (0.073, 0.025, 0.050, 0.061, 0.042, 0.072, 0.023, 0.053, 0.064, 0.089, 0.023, 0.043, 0.052, 0.040, 0.052, 0.073, 0.056, 0.063, 0.013, 0.033) (alphabetic order for the 20 amino acids).

Our parameter settings We used the default parameter settings in the SCA toolbox V5.0 [42].

Software availability `http://systems.swmed.edu/rr_lab/sca.html`.

2.11 Complementary: complementary matrix in Pearson coefficient [11] (2006)

Introduction To predict inter-protein residue coevolution, this study proposed a method which calculates Pearson's coefficients accounting for the complementary residues between protein interaction interfaces [11]. It is known that residue frequencies and residue pairs at protein-protein interfaces follow certain complementary patterns [44]. For instance, abundant hydrophobic residue pairs are often found at large protein interaction interfaces, while polar residue pairs usually occur at small interfaces [44]. Integrated into Pearson's coefficients, the complementary information was proven useful for inter-protein coevolution predictions. A promising performance of this method was found in the comparison with four other methods (MI, SCA, ELSC and OMES) using a sequence dataset containing 224 protein families in the Pfam database.

Methodology Let N be the number of sequences in the MSA input, S_i be the AA exchange matrix at the position i, $S_i(k,l)$ be the exchange score between the kth and l^{th} residues at position i, $\overline{S_i}$ and σ_i be the mean and the standard deviation of residues in the exchange matrix S_i at the position i, respectively. Given the kth sequence, $C_{i(k),j(k)}$ is the estimated complementary value between the kth residues at the position i and j [44]. The corrected Pearson's coefficient between the position i and j, termed $r_{i,j}$, is modeled as:

$$r_{i,j} = \frac{1}{N^2} \sum_{k,l=1}^{N} \frac{(S_{i(k,l)} - \overline{S_i})(S_{j(k,l)} - \overline{S_j}) \times C_{i(k),j(k)} \times C_{i(l),j(l)}}{\sigma_i \sigma_j} \tag{20}$$

Our parameter settings The complementary matrix of 20 amino acids in [44] was used and the other parameters were default.

Software availability Our toolbox.

2.12 PCC: Pearson correlation coefficient [12] (2010)

Introduction To reduce background noise and phylogenetic bias, this study proposed Pearson's correlation coefficients (PCC) for statistical coupling predictions [12].

Methodology Suppose N is the number of sequences in the MSA input, S_i is the exchange matrix at the position i, $S_i(k,l)$ is the AA exchange score between the kth and the lth residues at the position i, $\overline{S_i}$ and σ_i are the mean and standard deviation of residues in the exchange matrix S_i, respectively. The Pearson's coefficient between the positions i and j, termed $r_{i,j}$, is proposed as:

$$r(i,j) = \frac{1}{N^2} \sum_{k,l=1}^{N} \frac{(S_{i(k,l)} - \overline{S_i})(S_{j(k,l)} - \overline{S_j})}{\sigma_i \sigma_j} \tag{21}$$

$$\overline{S_i} = \frac{1}{N^2} \sum_{k,l=1}^{N} S_{i(k,l)}, \qquad \sigma_i = \sqrt{\frac{1}{N^2 - 1} \sum_{k,l=1}^{N} [S_{i(k,l)} - \overline{S_i}]^2} \tag{22}$$

Using the ASC to reduce the phylogenetic bias, the significance of statistical coupling is modeled as:

$$PCC(i,j) = r(i,\overline{x}) + r(j,\overline{x}) - \overline{r} \tag{23}$$

$$r(i,\overline{x}) = \frac{1}{N} \sum_{j=1}^{N} r(i,j), \qquad \overline{r} = \frac{1}{N} \sum_{i=1}^{N} r(i,\overline{x}) \tag{24}$$

Our parameter settings We used the default parameter settings.

Software availability Our toolbox.

2.13 LogR: disentangling direct coupling analysis [13] (2010)

Introduction The sequence-based method LogR was proposed to model the weighted covariations by disentangling indirect statistical dependencies from direct dependencies. Specifically, it quantifies the statistical couplings by estimating the weights of pairwise edges in Bayesian spanning trees, which can model the dependencies between residue positions [13]. In this s-tudy, the coevolving residue chains were found to travel through spatial distances in protein 3D

structures, indicating the indirect (or transitive) statistical dependencies. To reduce phylogenetic biases, LogR used the phylogenetic correction proposed by APC . Moreover, the statistical dependency was estimated using informative prior and conservation information, both of which could improve the accuracy of contact predictions [13].

Methodology Suppose the number of residue positions is N given the MSA input D. Measured through Dirichlet prior, $P(D_{i,j})$ is the joint probability of the position i and j. The statistical dependency between the ith and j^{th} positions, termed as $R_{i,j}$, is defined by the joint probability $P(D_{i,j})$ divided by the marginal probability $P(D_i)$ and $P(D_j)$.

$$R_{i,j} = \frac{P(D_{i,j})}{P(D_i)P(D_j)} \tag{25}$$

To avoid the existence of 0 in the statistical independency, $\log R_{i,j}$ is shifted to a non-negative value by dividing the minimal value of $\log R_{i,j}$, which is $S_{i,j} = \log(R_{i,j}/\min R_{i,j})$. The APC phylogenetic correction is then calculated as:

$$\log(R^C_{i,j}) = S_{i,j} - \frac{\sum_{n=1}^{N} S_{n,i} \sum_{m=1}^{N} S_{m,j}}{\sum_{n=1}^{N} \sum_{m=1}^{N} S_{n,m}} \tag{26}$$

To disentangle the statistical couplings, the weight of the edge $j \leftarrow \pi(j)$ in the entire spanning tree space is estimated using the priors $P(\pi)$, where $\pi(j)$ is the parent node of the jth position in the spanning tree π. The weighted correlation for the positions j and $\pi(j)$ is measured as:

$$M_{j,\pi(j)} = (R^C_{j,\pi(j)})^{\alpha} \frac{\mu_{j,\pi(j)}}{1 - \mu_{j,\pi(j)}} \tag{27}$$

Where $\pi(j)$ is the neighboring node of the node j^{th} in the spanning trees and $\mu_{j,\pi(j)}$ is the probability of the edge $j - \pi(j)$ in random spanning trees.

Our parameter settings We used the default parameter settings in the software.

Software availability Author's generosity.

2.14 DCA: direct coupling analysis [14] (2011)

Introduction This study aimed at the prediction of residue couplings in the spatial proximity given folded proteins [14]. Specifically, it approximates the maximum entropy by exploring the pairwise couplings given a MSA input. The implementation in [14] was proven to be fast compared to a message-passing algorithm published in an early study [45]. Moreover, the true positive rates of the new DCA has been shown to be better than MI and LogR using 131 domain families collected from the Pfam database.

Methodology Suppose $P(X_1, X_2, ..., X_n)$ is the join distribution given the MSA input with the residue positions X_i from 1 to n. Given a maximum-entropy function, the optimization of the joint distribution is approximated by the marginal and the pairwise dependencies using the Lagrange transformation. Specifically, the approximation strategies (i.e. independent positions, mean-field approximation) are used to determine parameters in the Gibbs potential functions. Similar to mutual information, DCA models the pairwise couplings using the direct information (DI):

$$DI_{ij} = \sum_{x_i} \sum_{x_j} P_{ij}^{(dir)}(x_i, x_j) \cdot \log \left[\frac{P_{ij}^{(dir)}(x_i, x_j)}{\sum_{x_i} P_{ij}^{(dir)}(x_i, x_j) \cdot \sum_{x_j} P_{ij}^{(dir)}(x_i, x_j)} \right] \quad (28)$$

Where x_i is the residue at the position i, $P_{ij}^{(dir)}(x_i, x_j)$ is estimated through the following Gibbs potential function:

$$P_{ij}^{(dir)}(x_i, x_j) = \frac{1}{Z_{ij}} exp[-(f_{ij}(x_i, x_j) - f_i(x_i)f_j(x_j))^{-1}(x_i, x_j) + \widetilde{h}_i(x_i) + \widetilde{h}_j(x_j)] \quad (29)$$

Where f_i is the marginal probability function of the position i^{th} in the MSA input and f_{ij} is the joint distribution between the positions i and j. $\widetilde{h}_i(x_i)$ is the parameter that imposes the empirical single-residue counts of residue x_i at the position i. Z_{ij} is the normalization parameter. $(f_{ij} - f_i f_j)^{-1}(x_i, x_j)$ is the element in the inverse of an empirical correlation matrix derived from the MSA input.

Our parameter settings We used the default parameter settings.

Software availability `http://plmdca.csc.kth.se/`.

2.15 PSICOV: precise structural contact prediction [15] (2012)

Introduction PSICOV uses a graphical Lasso approach with a sparse inverse covariance estimation to reduce prediction biases, caused by functionally related residue chains in protein structures [46]. The rationale of the sparse inverse covariance method relies on the fact that residue contacts are sparse in the known protein structures. Specifically, the non-zero terms in the sparse inverse covariance matrix represent coupling positions and the zero terms indicate that two positions are conditionally independent, assuming that the underlying distribution follows a multivariate Gaussian distribution [46].

Methodology The methodology of PSICOV can be simply described as:

$$\Theta = [\lambda \cdot diag(\overline{X_1}, ..., \overline{X_N}) + (1 - \lambda) \cdot Cov(X, Y)]^{-1} \tag{30}$$

Where N is the number of residues in a sequence, $Cov(X, Y)$ is the covariance matrix over all positions X, Y in the MSA input, Θ is the concentration matrix, $\lambda \in [0, 1]$ is the shrinkage parameter which targets diagonal values in $diag(\overline{X_1}, ..., \overline{X_N})$ and $\overline{X_i}$ denotes the mean of diagonal values in the covariance matrix. The inverse covariance matrix estimates the significance of the positions i and j in contact through the function $S_{ij}^C = \sum_{ab} |\Theta_{ij}^{ab}|$, where a and b are two residues at the position i and j, respectively. The correction of phylogenetic bias has also been taken into account in the PSICOV score:

$$PC_{ij} = S_{ij}^C - \frac{\sum_{i=1}^{N} S_{ij}^C \cdot \sum_{j=1}^{N} S_{ij}^C}{\sum_{i=1}^{N} \sum_{j=1}^{N} S_{ij}^C} \tag{31}$$

Our parameter settings Recommended by the software manual, the parameter $-r$ and $-i$ were set to 0.005 and 62, respectively. Other parameters were default.

Software availability `http://bioinfadmin.cs.ucl.ac.uk/downloads/PSICOV/`.

2.16 SVMcon: support vector machine contact map predictor [16] (2007)

Introduction SVMcon was designed to predict residue-residue contacts using support vector machines (SVM). It was ranked as the second best method in the 7th critical assessment of structure prediction (CASP7). SVMcon also outperformed CMAPpro using the benchmark datasets.

Methodology SVMcon begins with the assessment of five input features for each residue pair at the positions i and j. Using over 310000 training data points, the input features include the local window features, pairwise information features, residue type features, central segment window features and protein information features. Thereafter, the classification function of the SVM learner $f(x)$ is defined for contact predictions:

$$f(x) = \sum_{x_i \in S^+} \alpha_i \cdot K(x, x_i) - \sum_{x_i \in S^-} \alpha_i \cdot K(x, x_i) + b \tag{32}$$

$$K(x, x_i) = e^{-0.0025 \cdot ||x - x_i||^2} \tag{33}$$

Where x_i is the data point in a support vector, b represents the estimation bias, α_i is a non-negative weight assigned to the training data point x_i by minimizing a quadratic objective function. S^+ indicates the data point of residue pairs in contact and S^- indicates that the data point of a residue pair which is not in contact. A new data point x is predicted to be positive if $f(x) > 0$. $K(x, x_i)$ is the Gaussian radial basis kernel and the inverse of the variance parameter is optimized to be 0.0025.

Our parameter settings We used the default parameter settings.

Software availability `http://casp.rnet.missouri.edu/svmcon.html`

2.17 NNcon: neural network-based contact map predictor [17] (2009)

Introduction The 2D-Recursive Neural Network (2D-RNN) models were trained to predict residue-residue contacts using a sequence dataset consisted of 482 protein families.

Methodology Ten 2D-RNNs are trained and validated using the 10-fold cross-validation on a sequence dataset with 482 proteins. These 10 models are assembled to predict residues in con-

tact. The residue contacts are those residue pairs which have the Euclidean distance of their C_α atoms less than 8 or 12 angstroms. Given a protein sequence with the length of n amino acids, the 2D-RNN constructs a $n \times n$ input matrix and outputs a probability matrix with $n \times n$ contact elements.

Our parameter settings We used the default parameter settings.

Software availability `http://casp.rnet.missouri.edu/nncon.html`

2.18 DNcon: neural network contact prediction [18] (2012)

Introduction DNcon was proposed to improve the predictions of residue-residue contacts based on deep networks and boosting techniques [18]. Using the standard back propagation algorithm, the weights of multiple layers in deep networks were trained based on the predicted secondary structures, solvent accessibility, amino acid features and position-specific scoring matrix [18]. The method was evaluated using the D329 dataset containing 329 proteins, the SVMCON_TEST dataset containing 48 proteins and the CASP9 dataset containing 16 proteins. The comparison experiments showed that DNcon outperformed two other methods, ProC_S3 and SVMcon.

Methodology Amino acid features (e.g. electrostatic charge, codon diversity, volume, polarity, secondary structure) are modeled in deep network classifiers with the combination of restricted Bolzmann machines. The short, medium and long range residue-residue pairs are sampled from a large database, which is used to train deep network classifiers with the improved prediction power. The final contact predictions are evaluated by the scoring function.

Our parameter settings We used the software server with the option of the top $5L$ predictions where L is the length of amino acids given an input sequence.

Software availability `http://iris.rnet.missouri.edu/dncon/`.

2.19 CMPro: 2D recursive neural network [19] (2012)

Introduction This study proposed a contact prediction architecture based on neural networks and structural alignment models. Using both CASP8 and CASP9 datasets, performance of CMPro was shown to outperform PSICOV and other methods which were tested in CASP8 and CASP9 contact prediction [19].

Methodology Neural network prediction models are constructed in three steps. Firstly, the coarse contacts and the orientations between secondary structure elements are predicted using 2D recursive neural networks. The probability of parallel contact, anti-parallel contact or no-contact is estimated using feature variables extracted from protein secondary structures and amino acid compositions in the sequence input. Secondly, the energy-based method is used to optimize the amino acid alignment of strand - strand and helix-helix secondary structures. The log-likelihood objective function is defined as:

$$E_A = -\sum_{i=1}^{n} \log P_A(\hat{a}_i, \hat{\theta}_i) \tag{34}$$

Where n is the number of anti - parallel (or parallel) contacting residue pairs, $\hat{a}_i$ and $\hat{\theta}_i$ are the true shift and phase for the ith example, respectively. Thirdly, a deep neural network architecture refines the prediction of residue contacts. Deep NN architecture has k layers and each layer contains $25 \times 9 \times 2$ residue features, $3 \times 7 \times 7$ coarse features, $4 \times 7 \times 7$ alignment features and 81 temporal features. Due to the heavy computation of backpropagated gradients in multi-layered neural networks, an incremental approach has been proposed to train the weights of neural networks. The prediction performance is further improved using 10-fold cross-validation.

Our parameter settings We used the default parameter settings.

Software availability `http://scratch.proteomics.ics.uci.edu/`

2.20 PhyCMAP: random forest, integer linear programming [20] (2013)

Introduction This study proposed a sequence-based method PhyCMAP, which integrates both evolutionary and physical restraints using random forests and integer linear programming approach [20]. The performance comparison showed that PhyCMAP outperformed NNcon, CMAP-

pro and DCA given the CASP10 dataset.

Methodology PhyCMAP has two components. The first component predicts the probability score of the residue contacts using random forests. The contact score for the position pair (i, j) is defined as:

$$HPS(i,j) = \sum_{h \in H} PS_{beta}(a_i^h, a_j^h) + PS_{helix}(a_i^h, a_j^h) \tag{35}$$

Where a_i^h is the residue in a homology sequence h aligned to the residue i in the query sequence, $PS_{beta}(a_i^h, a_j^h)$ is the probability of a residue pair (a_i^h, a_j^h) forming a contact in the β-sheet structure, $PS_{helix}(a_i^h, a_j^h)$ is the probability of residues a_i^h, a_j^h forming a contact connecting two helix structures. Both $PS_{helix}(a_i^h, a_j^h)$ and $PS_{beta}(a_i^h, a_j^h)$ are obtained from protein structures in the training dataset containing 900 non-redundant protein structures

The second component selects a set of top-ranked contacts by using the integer linear programming, which maximizes accumulative probabilities under a set of physical constraints.

$$\max_{X,R} \sum_{6 \leq j-i} (X_{i,j} \times HSP(i,j)) - g(R) \tag{36}$$

Where $X_{i,j}$ is a binary variable and $X_{i,j} = 1$ if the position pair (i, j) are in contact. $g(R) = \sum R_r$ is a linear penalty function with the parameter r defined over 8 hard and soft constrains. These constraints are mainly proposed based on the observations of residue contacts between two β-strand structures or between two α-helix structures.

Our parameter settings We used the default parameter settings.

Software availability `http://raptorx.uchicago.edu/`

2.21 Mutagenetic: mutagenetic tree mixture model [21] (2005)

Introduction Mutagenetic tree models have been designed to investigate the accumulation of drug resistance-associated mutations in HIV-1 proteins [21]. It was shown that mutagenetic tree mixture models could identify many parallel or confluent mutation pathways using sequence datasets of HIV-1 protease [21]. This method has also been applied to the field of tumor

development [47,48].

Methodology The mutagenetic tree model is built using a set of directed weighted trees. This model can approximate the joint probability distribution consisting of a set of mutational events. Based on a similar methodology proposed in the mixed tree probabilistic models [49], EM algorithm can be used to maximize the log-likelihood function. Suppose a mutagenetic tree is denoted as $T = (V, E)$ with the set of vertices V and the set of edges E, $x = \{x_i, ..., x_N\}$ represents the mutation pattern. Given one mutagenetic tree model T, the likelihood of a pattern x in T can be modeled as:

$$L(x|T) = \prod_{e \in E(V(x))} P(e) \times \prod_{e \in E(V-V(x))} (1 - P(e)) \tag{37}$$

The mixed tree model is defined by $M = \sum_{k=1}^{K} \alpha_k T_k$, where $\alpha_k \in [0, 1]$ is the weight of the kth tree T_k and $\sum_{k=1}^{K} \alpha_k = 1$. The likelihood of the trained mutagenetic tree M is defined as:

$$f(x_1, ..., x_N|M) = \sum_{i=1}^{N} \log \sum_{k=1}^{K} \alpha_k L(x_i|T_k) \tag{38}$$

The parameters α_k and T_k are thereafter optimized by the EM algorithm [49].

Our parameter settings For each input sequence dataset, one mutagenetic tree was created using default settings.

Software availability `http://mtreemix.bioinf.mpi-inf.mpg.de/`.

2.22 BN: Bayesian network [22] (2007)

Introduction Bayesian networks (BNs) have been used to model mutational pathways in HIV-1 proteins [22, 50, 51]. It was shown that Bayesian networks could be useful for improving drug resistance predictions [52].

Methodology Bayesian networks are trained based on methods adapted from the Bright software [53]. Given a MSA input, this method maximizes the posterior probability of Bayesian networks whose variables are residues or therapies. The robustness of Bayesian networks is ex-

amined by a non-parametric bootstrap resampling using 100 replicates. In a consensus Bayesian network, edges between variable nodes are considered as robust if their bootstrap supports are above 65%. Different amino acids at the same position may cluster together due to the presence of strong antagonistic effects.

Our parameter settings We used the default parameters as indicated in [22] (bootstrap resampling: 100 replicates, bootstrap support: 65%). In order to compare results of Bayesian networks with other coevolution methods, we extracted position pairs of adjacent variables in the trained Bayesian networks.

Software availability `https://code.google.com/p/bright/`.

2.23 Spidermonkey [23] (2008)

Introduction In the software platform Spidermonkey, coevolving positions are modeled using Bayesian networks trained using reconstructed ancestral sequences from a phylogenetic tree [23]. More specifically, Spidermonkey uses MCMC-based algorithms to model conditional dependencies between the non-synonymous positions in Bayesian networks. The advantage of this method relies on the fact that Bayesian networks can model high order interactions using ancestral sequences, for coevolving positions may have high-order interactions in protein families [23]. However, the convergence of MCMC in the process of Bayesian network training requires large sequence datasets and a heavy demand of computation power. As a compromise between computing power and prediction accuracy, Spidermonkey allows for at most two parent nodes of variables in Bayesian networks [23].

Methodology Given a sequence dataset, Spidermonkey estimates a substitution model and reconstructs ancestral sequences in the phylogenetic tree using a strategy of maximum-likelihood optimization. The statistical dependencies between protein positions in Bayesian networks are modeled using a MCMC-based algorithm [23].

Our parameter settings In our analysis, we used parameter settings as follows: (1) nucleotide model 012345, MG94x; (2) treatment of ambiguities: averaged; (3) the number of positions with substitution values: default, (5) maximum parents: 2; (6) the number of MCMC chains:

default (100000); (7) the number of burn-in steps before the main chain: default (10000); (8) the number of ancestral samples: default (100).

Software availability `http://www.hyphy.org/w/index.php/Main_Page`.

2.24 CTMP: continuous time Markov process [24] (2007)

Introduction This study proposed a continuous-time Markov process model augmented with the phylogenetic information [24]. To identify sequence coevolution in different protein families, the model was applied to screen all position pairs of inter- and intra-domains in the protein families [24]. The majority of coevolving protein domains was found near functionally important positions, providing an interesting information of protein structural and functional constraints in the sequence coevolution [24].

Methodology Four steps are performed to measure the residue coevolution. Firstly, the matrix of coevolutionary rates corresponding to amino acid changes is obtained by reweighting the independent coevolutionary rates. Secondly, sequences from different protein domains are mapped to the leaves of phylogenetic trees shared with the same topology. Thirdly, the log-likelihood ratio is measured by the likelihood of observed sequences in the coevolution model compared to the null model. By doing so, the joint probability of residue positions is approximated by the singlet and pairwise terms of aligned positions among all states of internal nodes in the phylogenetic tree. The CTMP model is simplified as:

$$P(x_1(t), ..., x_n(t)|x_1(0), ..., x_n(0)) = \frac{\prod\limits_{x_i - x_j \in \pi} P(x_i(t), x_j(t)|x_i(0), x_j(0))}{\prod\limits_{i=1}^{n} P(x_i(t)|x_i(0))^{d_i - 1}} \tag{39}$$

Where $x_i(t)$ is the sequence composition at the ith position with the sampling time t given the phylogenetic tree π and the MSA input. The position pair $x_i(t), x_j(t)$ is observed on the leaves of the phylogenetic tree π.

Lastly, the false positive rates of coevolving position pairs are evaluated by multiple hypothesis tests using the simulated datasets.

Our parameter settings We used maximum 500 sequences for our CTMP analysis due to

limited computation power. The maximum-likelihood phylogenetic trees were trained using RAxML V7.0.4. The threshold on the fraction of sequences coevolving with non-overlapping states was set to 1, as well as the threshold on the fraction of conserved sequences. The penalty parameter (ϵ) of the CTMP model was set to 0.25 according to the manual.

Software availability `http://www.stat.sinica.edu.tw/chyeang/`.

2.25 CoMap [25] (2011)

Introduction CoMap V1.4.1 uses Markov models to identify residue coevolution based on the phylogenetic tree [25]. This model takes into account the uncertainty of ancestral states and among-site rate variations given the phylogenetic tree inputs [25]. As an advantage, CoMap can work on both nucleotide and amino acid sequence datasets. Using a ribosomal RNA dataset including 79 bacteria species, this method identified more than 95% intra-protein predictions based on protein contact maps [25].

Methodology Firstly, for each residue position, CoMap creates a substitution vector which contains posterior estimates of the substitutions at each branch given a phylogenetic tree. By accounting for position variations, this substitution vector is defined as:

$$v_{i,b} = \sum_{c}\sum_{x_p}\sum_{x_q} n_{x_p,x_q}(t \cdot r_c) \times P(x_p, x_q, r_c|D_i, \Theta) \tag{40}$$

Where D_i is the ith position of the MSA input D, a is the number of internal nodes in the phylogenetic tree and b is a branch in the phylogenetic tree, r_c is the rate of class c, Θ is the set of parameters including branch lengths, substitution matrices and rate distribution parameters. $n_{x_p,x_q}(t{\cdot}r_c)$ is the conditional observation of substitutions expected on the branch with its branch length t and the states x_p, x_q.

Secondly, the Pearson's correlation coefficient between two substitution vectors is:

$$\rho_{i,j} = \frac{Cov(V_i, V_j)}{\sigma(V_i)\sigma(V_j)} \tag{41}$$

Where $V_i = (v_{i,1}, ..., v_{i,b}, ..., v_{i,m})$ is obtained in the first step and $\sigma(V_i)$ is the standard deviation of V_i.

Thirdly, to show the statistical significance, p-values are measured by comparing the conditional observations of substitutions with the expectation of the null hyphothesis of independence. The null distribution is estimated by simulating 100000 independent pairs [25].

Our parameter settings We used pairwise analysis to calculate the compensation coefficient for each position pair in CoMap V1.4.1 [54]. Due to the limitation of our computation power, the number of sequences in the input datasets was restrained to be less than 500 sequences. In the first round, CoMap reported varied positions in the maximum likelihood phylogenetic tree. We thereafter removed positions reported with infinite maximum likelihood according to the software manual. In the second round, the maximum-likelihood phylogenetic trees were prepared using RAxML V7.0.4. As suggested in the software manual, we used the following parameters: (1) nijt_aadist.sym=no, (2) aadist.type=grantham, (3) statistic=Compenstation, (4) model=LG08, (5) statistic.null=yes, (6) statistic.null.compute_pvalue=yes, and (7) statistic.null.nb_rep_CPU=8. The coevolving residues were collected if their p-values were less than 0.05.

Software availability `http://gna.org/projects/comap`.

2.26 GREMLIN: generative regularized models of proteins [26] (2013)

Introduction GREMLIN was originally proposed to learn an undirected probabilistic graphical model of the amino acid compositions given the inputs of MSA [55]. GREMLIN outperformed hidden Markov models using the datasets of 71 protein families extracted from the PFAM database [55]. As sequence-based methods usually require a large amount of sequences to achieve robust predictions, this paper contributes to incorporate prior information on residue pairs so that fewer sequences are needed for robust coevolution predictions [26]. Performance of GREMLIN was compared to MI, PSICOV, DCA, plmDCA and MIc using a large sequence dataset with 329 protein families.

Methodology Markov random field is used to model the probability distribution given a set of independent sequences $X = \{X^1, X^2, ..., X^N\}$ in the MSA input. Due to the intractable computation of the global maximum likelihood function, the pseudo-likelihood function is proposed for the efficient approximation to make the problem solvable. Specifically, the pseudo-

likelihood is defined as:

$$pll(\Phi) = \frac{1}{N} \sum_{X^i \in X} \sum_{j=1}^{N} \left[\log \phi_j(X_j^i) + \sum_{k \in ne(V_j)} \log \phi_{jk}(X_j^i, X_k^i) - \log Z_j \right] \tag{42}$$

Where X_j^i is the residue at the jth position of the ith sequence given the MSA input, ϕ_j is the potential function of the position j, ϕ_{jk} is the potential function for the edge $j-k$, Z_j is a local normalization constant, $ne(V_j)$ is the set of vertices connected with the node j in the undirected graphical model.

For both structure learning and parameter estimation, the L1 regularization is used for the optimization based on the projected gradients.

$$max_{\Phi,\alpha} \quad pll(\Phi) - \lambda_{node} \sum_{s=1}^{N} ||V^s||_2^2 - \lambda_{edge} \sum_{s=1}^{N} \sum_{t=s+1}^{N} \alpha_{st} \tag{43}$$

$$subject \quad to: \quad \forall (1 \leq s < t \leq N) : \alpha_{st} \geq ||W^{st}||_2 \tag{44}$$

Where λ_{node} and λ_{edge} are regularization parameters that determine the weights of the penalty level. α_{st} is the differentiable proxy of $||W^{st}||_2$, which solves the calculation using a smooth convex optimization.

Our parameter settings We used the default parameter settings.

Software availability `http://openseq.org/`.

3 Implementation and software manual

Several sequence-based methods (e.g. SCA, LogR, DCA, GREMLIN, ZERS, NBZPX2) were designed using Matlab, we thus decided to build up the ensemble coevolution system in Matlab by integrating the available methods and implementing 7 methods without public sources. We newly implemented 7 methods in our toolbox including ASC, APC, Complementary, NCPS, PCC, PhysicoMI and RCW. Other methods designed in non-Matlab platform were requested from the authors or downloaded from public sources (see Table 1).

Due to the copy right issue, users who wish to test methods implemented in other publica-

tions need to install software independently. Nevertheless, our toolbox offers users the platform to prepare the input files, the command lines (if any), the extraction of output results and the assembly of all 27 methods integrated in our system. Currently, the toolbox V0.1 has only been tested in Linux (Ubuntun 12.04, 64-bit). If system comparability or other issues were encountered, please write an email to liguangdi.research@gmail.com to obtain the latest version.

References

1. Brandman R, Brandman Y, Pande VS (2012) Sequence coevolution between rna and protein characterized by mutual information between residue triplets. PloS one 7: e30022.
2. Dunn SD, Wahl LM, Gloor GB (2008) Mutual information without the influence of phylogeny or entropy dramatically improves residue contact prediction. Bioinformatics 24: 333–340.
3. Gouveia-Oliveira R, Pedersen AG, et al. (2007) Finding coevolving amino acid residues using row and column weighting of mutual information and multi-dimensional amino acid representation. Algorithms Mol Biol 2: 12.
4. Little DY, Chen L (2009) Identification of coevolving residues and coevolution potentials emphasizing structure, bond formation and catalytic coordination in protein evolution. PloS one 4: e4762.
5. Tillier ER, Lui TW (2003) Using multiple interdependency to separate functional from phylogenetic correlations in protein alignments. Bioinformatics 19: 750–755.
6. Ackerman SH, Tillier ER, Gatti DL (2012) Accurate simulation and detection of coevolution signals in multiple sequence alignments. PloS one 7: e47108.
7. Gao H, Dou Y, Yang J, Wang J (2011) New methods to measure residues coevolution in proteins. BMC bioinformatics 12: 206.
8. Kalinina OV, Oberwinkler H, Glass B, Kräusslich HG, Russell RB, et al. (2012) Computational identification of novel amino-acid interactions in hiv gag via correlated evolution. PloS one 7: e42468.
9. Lee BC, Kim D (2009) A new method for revealing correlated mutations under the structural and functional constraints in proteins. Bioinformatics 25: 2506–2513.
10. Reynolds KA, McLaughlin RN, Ranganathan R (2011) Hot spots for allosteric regulation on protein surfaces. Cell 147: 1564–1575.
11. Halperin I, Wolfson H, Nussinov R (2006) Correlated mutations: Advances and limitations. a study on fusion proteins and on the cohesin-dockerin families. Proteins: Structure, Function, and Bioinformatics 63: 832–845.
12. Ashkenazy H, Kliger Y (2010) Reducing phylogenetic bias in correlated mutation analysis. Protein Engineering Design and Selection 23: 321–326.

13. Burger L, van Nimwegen E (2010) Disentangling direct from indirect co-evolution of residues in protein alignments. PLoS computational biology 6: e1000633.

14. Morcos F, Pagnani A, Lunt B, Bertolino A, Marks DS, et al. (2011) Direct-coupling analysis of residue coevolution captures native contacts across many protein families. Proceedings of the National Academy of Sciences 108: E1293–E1301.

15. Jones DT, Buchan DW, Cozzetto D, Pontil M (2012) Psicov: precise structural contact prediction using sparse inverse covariance estimation on large multiple sequence alignments. Bioinformatics 28: 184–190.

16. Cheng J, Baldi P (2007) Improved residue contact prediction using support vector machines and a large feature set. BMC bioinformatics 8: 113.

17. Tegge AN, Wang Z, Eickholt J, Cheng J (2009) Nncon: improved protein contact map prediction using 2d-recursive neural networks. Nucleic Acids Research 37: W515–W518.

18. Eickholt J, Cheng J (2012) Predicting protein residue–residue contacts using deep networks and boosting. Bioinformatics 28: 3066–3072.

19. Di Lena P, Nagata K, Baldi P (2012) Deep architectures for protein contact map prediction. Bioinformatics 28: 2449–2457.

20. Wang Z, Xu J (2013) Predicting protein contact map using evolutionary and physical constraints by integer programming. Bioinformatics 29: i266–i273.

21. Beerenwinkel N, Rahnenführer J, Däumer M, Hoffmann D, Kaiser R, et al. (2005) Learning multiple evolutionary pathways from cross-sectional data. Journal of Computational Biology 12: 584–598.

22. Deforche K, Silander T, Camacho R, Grossman Z, Soares M, et al. (2006) Analysis of hiv-1 pol sequences using bayesian networks: implications for drug resistance. Bioinformatics 22: 2975–2979.

23. Poon AF, Lewis FI, Frost SD, Pond SLK (2008) Spidermonkey: rapid detection of co-evolving sites using bayesian graphical models. Bioinformatics 24: 1949–1950.

24. Yeang CH, Haussler D (2007) Detecting coevolution in and among protein domains. PLoS computational biology 3: e211.

25. Dutheil J, Pupko T, Jean-Marie A, Galtier N (2005) A model-based approach for detecting coevolving positions in a molecule. Molecular biology and evolution 22: 1919–1928.

26. Kamisetty H, Ovchinnikov S, Baker D (2013) Assessing the utility of coevolution-based residue–residue contact predictions in a sequence-and structure-rich era. Proceedings of the National Academy of Sciences 110: 15674–15679.

27. Polikar R (2006) Ensemble based systems in decision making. Circuits and Systems Magazine, IEEE 6: 21–45.

28. Polikar R (2012) Ensemble learning. In: Ensemble Machine Learning, Springer. pp. 1–34.

29. Troć M, Unold O (2010) Self-adaptation of parameters in a learning classifier system ensemble machine. International Journal of Applied Mathematics and Computer Science 20: 157–174.

30. Gao Y, Huang JZ, Wu L (2007) Learning classifier system ensemble and compact rule set. Connection Science 19: 321–337.

31. Bacardit J, Krasnogor N (2008) Empirical evaluation of ensemble techniques for a pittsburgh learning classifier system. In: Learning Classifier Systems, Springer. pp. 255–268.

32. Gama J, Brazdil P (2000) Cascade generalization. Machine Learning 41: 315–343.

33. Saha I, Zubek J, Klingstrom T, Forsberg S, Wikander J, et al. (2014) Ensemble learning prediction of protein-protein interactions using proteins functional annotations. Molecular BioSystems .

34. Yang J, Jang R, Zhang Y, Shen HB (2013) High-accuracy prediction of transmembrane inter-helix contacts and application to gpcr 3d structure modeling. Bioinformatics 29: 2579–2587.

35. Ekeberg M, Lövkvist C, Lan Y, Weigt M, Aurell E (2013) Improved contact prediction in proteins: Using pseudolikelihoods to infer potts models. Physical Review E 87: 012707.

36. Cover TM, Thomas JA (2012) Elements of information theory. Wiley-interscience.

37. Bremm S, Schreck T, Boba P, Held S, Hamacher K (2010) Computing and visually analyzing mutual information in molecular co-evolution. BMC bioinformatics 11: 330.

38. Bielza C, Li G, Larranaga P (2011) Multi-dimensional classification with bayesian networks. International Journal of Approximate Reasoning 52: 705–727.

39. Lockless SW, Ranganathan R (1999) Evolutionarily conserved pathways of energetic connectivity in protein families. Science 286: 295–299.

40. Süel GM, Lockless SW, Wall MA, Ranganathan R (2002) Evolutionarily conserved networks of residues mediate allosteric communication in proteins. Nature Structural & Molecular Biology 10: 59–69.

41. Russ WP, Lowery DM, Mishra P, Yaffe MB, Ranganathan R (2005) Natural-like function in artificial ww domains. Nature 437: 579–583.

42. Halabi N, Rivoire O, Leibler S, Ranganathan R (2009) Protein sectors: evolutionary units of three-dimensional structure. Cell 138: 774–786.

43. Bartlett GJ, Taylor WR (2008) Using scores derived from statistical coupling analysis to distinguish correct and incorrect folds in de-novo protein structure prediction. Proteins: Structure, Function, and Bioinformatics 71: 950–959.

44. Glaser F, Steinberg DM, Vakser IA, Ben-Tal N (2001) Residue frequencies and pairing preferences at protein–protein interfaces. Proteins: Structure, Function, and Bioinformatics 43: 89–102.

45. Weigt M, White RA, Szurmant H, Hoch JA, Hwa T (2009) Identification of direct residue contacts in protein–protein interaction by message passing. Proceedings of the National Academy of Sciences 106: 67–72.

46. Friedman J, Hastie T, Tibshirani R (2008) Sparse inverse covariance estimation with the graphical lasso. Biostatistics 9: 432–441.

47. Rahnenführer J, Beerenwinkel N, Schulz WA, Hartmann C, Von Deimling A, et al. (2005) Estimating cancer survival and clinical outcome based on genetic tumor progression scores. Bioinformatics 21: 2438–2446.

48. Desper R, Jiang F, Kallioniemi OP, Moch H, Papadimitriou CH, et al. (1999) Inferring tree models for oncogenesis from comparative genome hybridization data. Journal of Computational Biology 6: 37–51.

49. Meila M, Jordan MI (2001) Learning with mixtures of trees. The Journal of Machine Learning Research 1: 1–48.

50. Deforche K, Camacho R, Grossman Z, Silander T, Soares M, et al. (2007) Bayesian network analysis of resistance pathways against hiv-1 protease inhibitors. Infection, Genetics and Evolution 7: 382–390.

51. Deforche K, Camacho RJ, Grossman Z, Soares MA, Van Laethem K, et al. (2008) Bayesian network analyses of resistance pathways against efavirenz and nevirapine. Aids 22: 2107–2115.

52. Deforche K, Camacho R, Van Laethem K, Lemey P, Rambaut A, et al. (2008) Estimation of an in vivo fitness landscape experienced by hiv-1 under drug selective pressure useful for prediction of drug resistance evolution during treatment. Bioinformatics 24: 34–41.

53. Myllymäki P, Silander T, Tirri H, Uronen P (2002) B-course: A web-based tool for bayesian and causal data analysis. International Journal on Artificial Intelligence Tools 11: 369–387.

54. Dutheil J, Galtier N (2007) Detecting groups of coevolving positions in a molecule: a clustering approach. BMC evolutionary biology 7: 242.

55. Balakrishnan S, Kamisetty H, Carbonell JG, Lee SI, Langmead CJ (2011) Learning generative models for protein fold families. Proteins: Structure, Function, and Bioinformatics 79: 1061–1078.
