# Supplementary material for: A new ensemble coevolution system for detecting HIV-1 protein coevolution
Source: Biol Direct. 2015 Jan 7;10:1. doi: 10.1186/s13062-014-0031-8 (PMC4332441; doi:10.1186/s13062-014-0031-8)
Supplement: Additional file 2: Figure S1. — Evaluation of the method combination CNPR and the 27 individual methods applied to the 7 HIV-1 datasets. Figure S2. Jaccard and association coefficients between CNPR and 27 sequence-based methods. Figure S3. HIV-1 nucleocapsid coevolving pairs predicted by CNPR. Figure S4. Contact map of HIV-1 protease and coevolving pairs predicted by 28 sequence-based methods. Figure S5. Contact map of HIV-1 matrix and coevolving pairs predicted by 28 sequence-based methods. Figure S6. Contact map of HIV-1 capsid and coevolving pairs predicted by 28 sequence-based methods. Figure S7. Contact map of HIV-1 nucleocapsid and coevolving pairs predicted by 28 sequence-based methods. Table S1. Summary of PI-associated Gag and protease substitutions reported in the experimental or clinical studies. Table S2. Summary of 7 sequence datasets for detecting HIV-1 coevolution. Table S3. Ranking of sequence-based methods using individual HIV-1 datasets. Table S4. Accuracy of sequence-based methods on individual HIV-1 datasets (threshold of contact distance: 8 angstroms). Table S5. Harmonic distance of sequence-based methods using individual HIV-1 datasets (Å). Table S6. Average Euclidean distance (Å) of the top-ranked long-range couplings predicted by sequence-based methods. Table S7. Summary of long-range residue contacts derived from HIV-1 Gag and protease protein structures. Table S8. Accuracy of sequence-based methods on individual HIV-1 datasets (threshold of contact distance: 5 angstroms). [file 13062_2014_31_MOESM2_ESM.pdf]

Supplementary figures and tables

Title: A new ensemble coevolution system for detecting HIV-1 protein coevolution

Authors and Affiliations

Guangdi Li[1], Kristof Theys[1], Jens Verheyen[2], Andrea-Clemencia Pineda-Peña[1,3], Ricardo Khouri[1], Supinya Piampongsant[1], Mónica Eusébio[4], Jan Ramon[5], Anne-Mieke Vandamme[1,4,*]

Affiliations

1 Clinical and Epidemiological Virology, Rega Institute for Medical Research, Department of Microbiology and Immunology, KU Leuven - University of Leuven, Leuven, Belgium

2 Institute of Virology, University hospital, University Duisburg-Essen, Essen, Germany

3 Clinical and Molecular Infectious Disease Group, Faculty of Sciences and Mathematics, Universidad del Rosario, Bogotá, Colombia

4 Centro de Malária e Outras Doenças Tropicais and Unidade de Microbiologia, Instituto de Higiene e Medicina Tropical, Universidade Nova de Lisboa, Lisboa, Portugal

5 Department of Computer Science, KU Leuven - University of Leuven, Leuven, Belgium

1. Supplementary figures

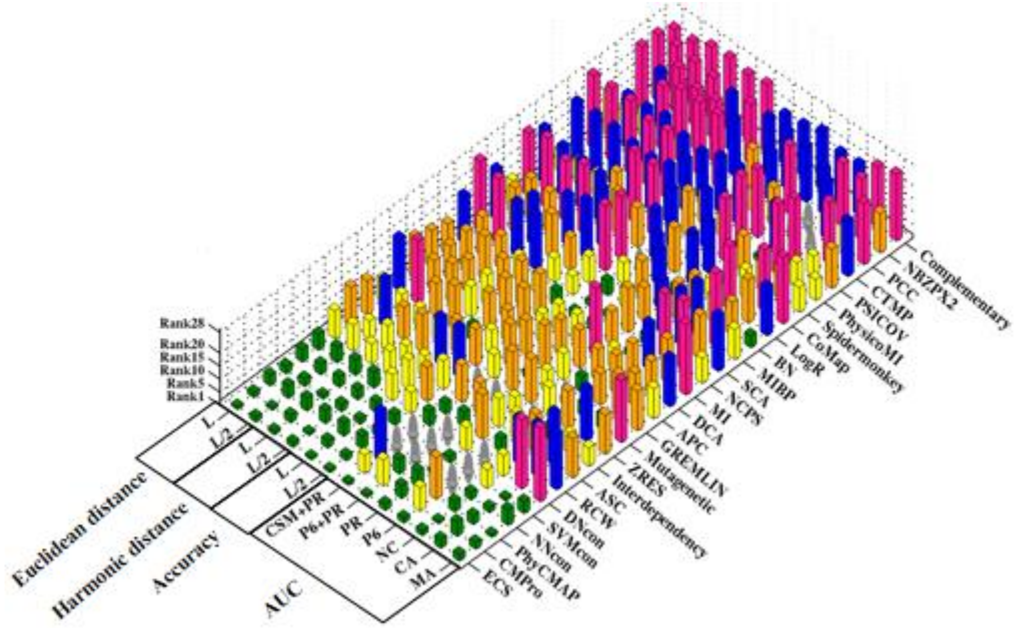

Figure S 1: Evaluation of the method combination CNPR and the 27 individual methods applied to the 7 HIV-1 datasets. The x-axis indicates the 28 assessed methods which are ordered according to their performance ranking (CNPR with the best ranking is positioned on the left). The y-axis indicates four statistical measurements (AUC, accuracy, harmonic distance, Euclidean distance) used for the assessment of coevolution predictions given 7 HIV-1 datasets. The L and L/2 top-ranked predictions are evaluated by the measurements of accuracy, harmonic distance and Euclidean distance. The z-axis indicates the performance ranking of individual methods, where one method with the best ranking has the shortest bar. Based on the performance ranking, bars are colored by green (ranking: 1-5), yellow (ranking: 6-10), orange (ranking: 11-15), blue (ranking: 16-20) and red (ranking: 21-28). Grey cones are used when no long-range couplings were predicted by the corresponding sequence-based methods (e.g. AUC is not evaluated for NNcon in predicting long-range couplings in the p6 protein). Ranking data is provided in Table S3.

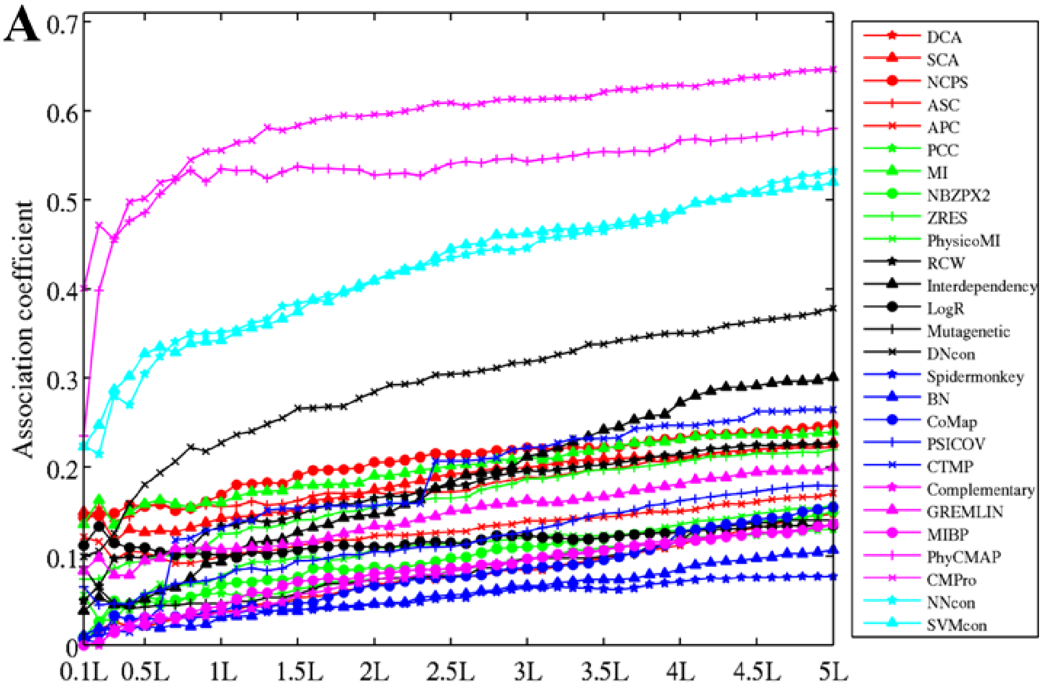

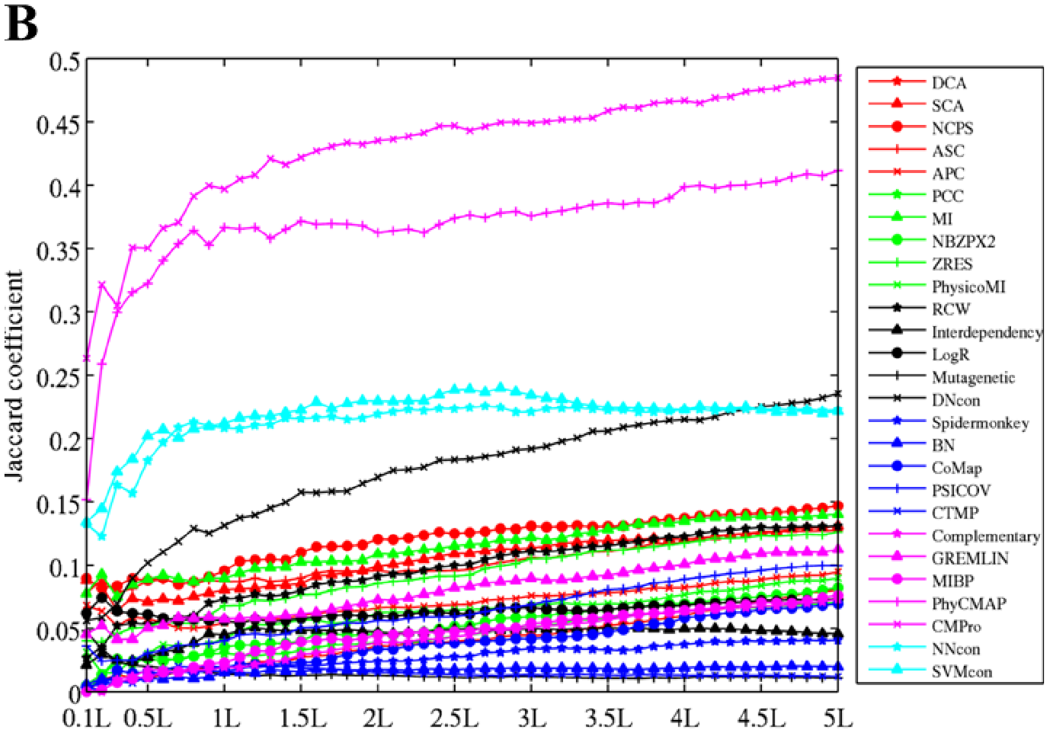

Figure S 2: Jaccard and association coefficients between CNPR and 27 sequence-based methods. The x-axis indicates the cutoff of the top-ranked couplings used for the calculation of Jaccard and association coefficients, where L is the number of AA sequence in the sequence inputs.

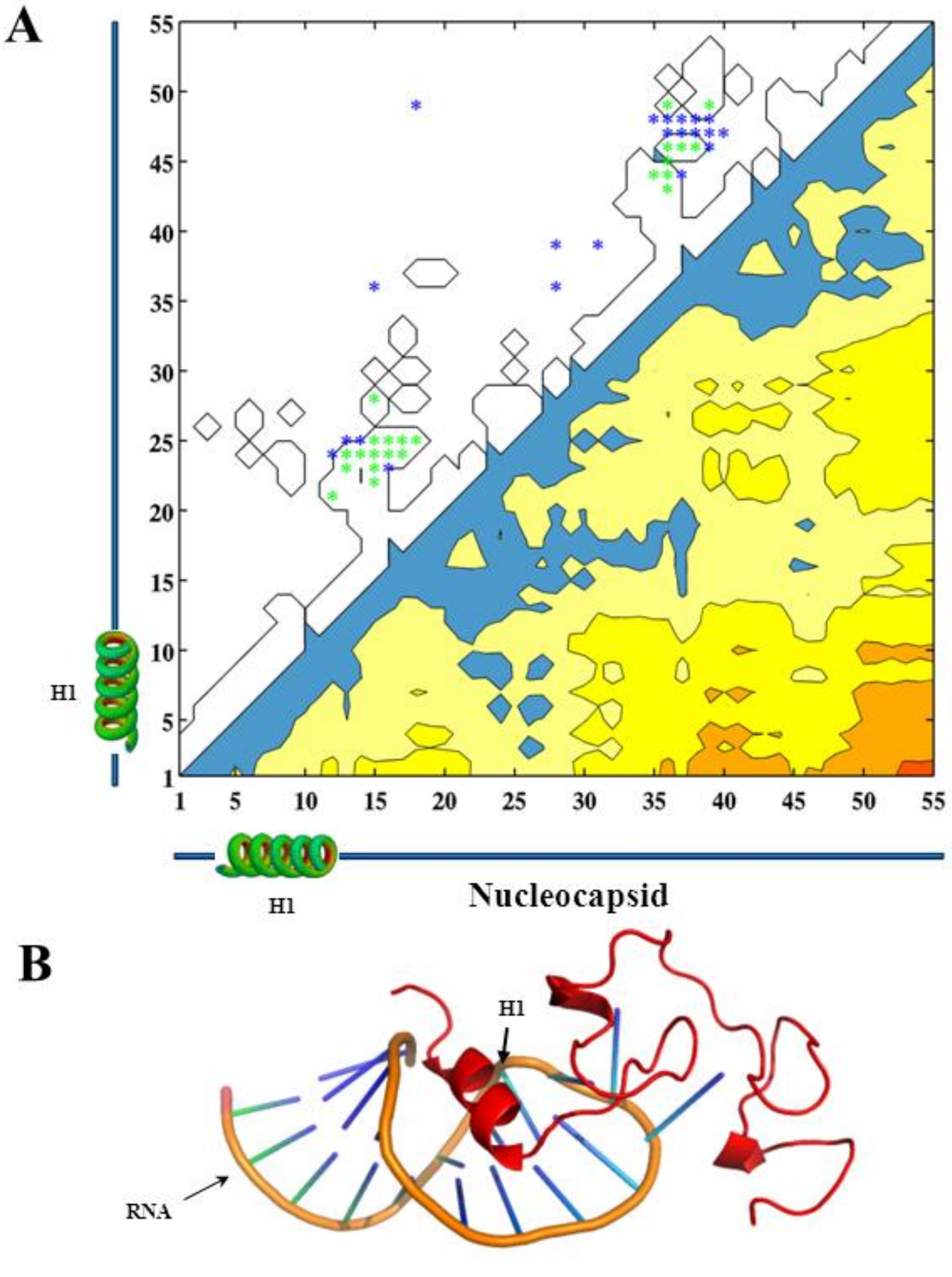

Figure S 3: HIV-1 nucleocapsid coevolving pairs predicted by CNPR. (a) The contact map of HIV-1 nucleocapsid (bottom right) and the predicted coevolving pairs (top left) are illustrated. Green dots indicate true positive pairs predicted in protein contact map. The helix secondary structure H1 is shown along the x- and y-axes. (b) HIV-1 nucleocapsid structure crystalized with a RNA stem loop. PDB code: 1A1T. Visualization software: Matlab and PyMOL V1.5.

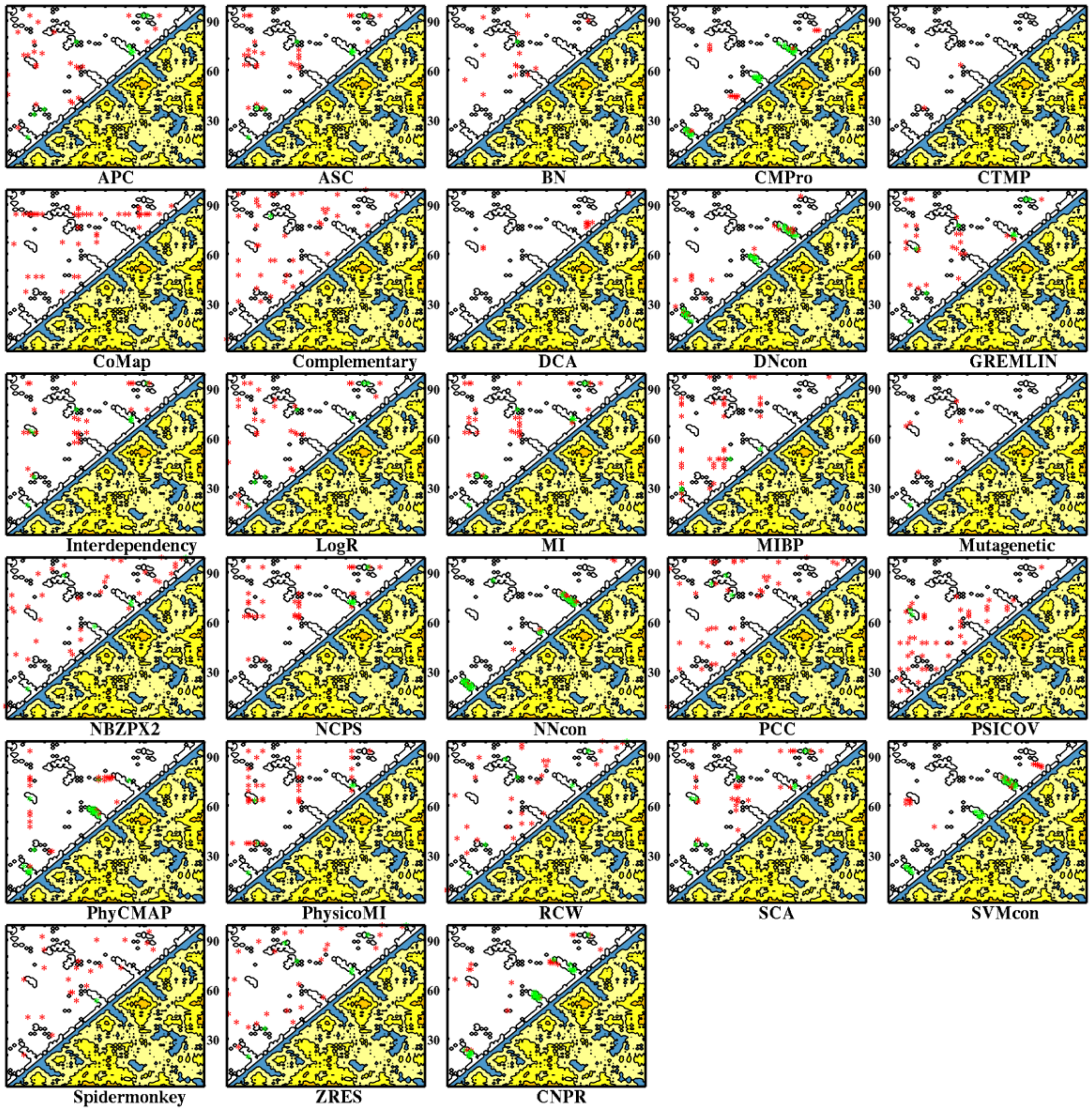

Figure S 4: Contact map of HIV-1 protease and coevolving pairs predicted by 28 sequence-based methods. For each subplot, the protein contact map is shown at the bottom right and the top-ranked coevolving residues (L=99) predicted by sequence-based methods are shown as asterisk in the upper left side. True positive coevolving pairs are those falling within the contours of the protein contact map, and indicated as green asterisk. The others are red.

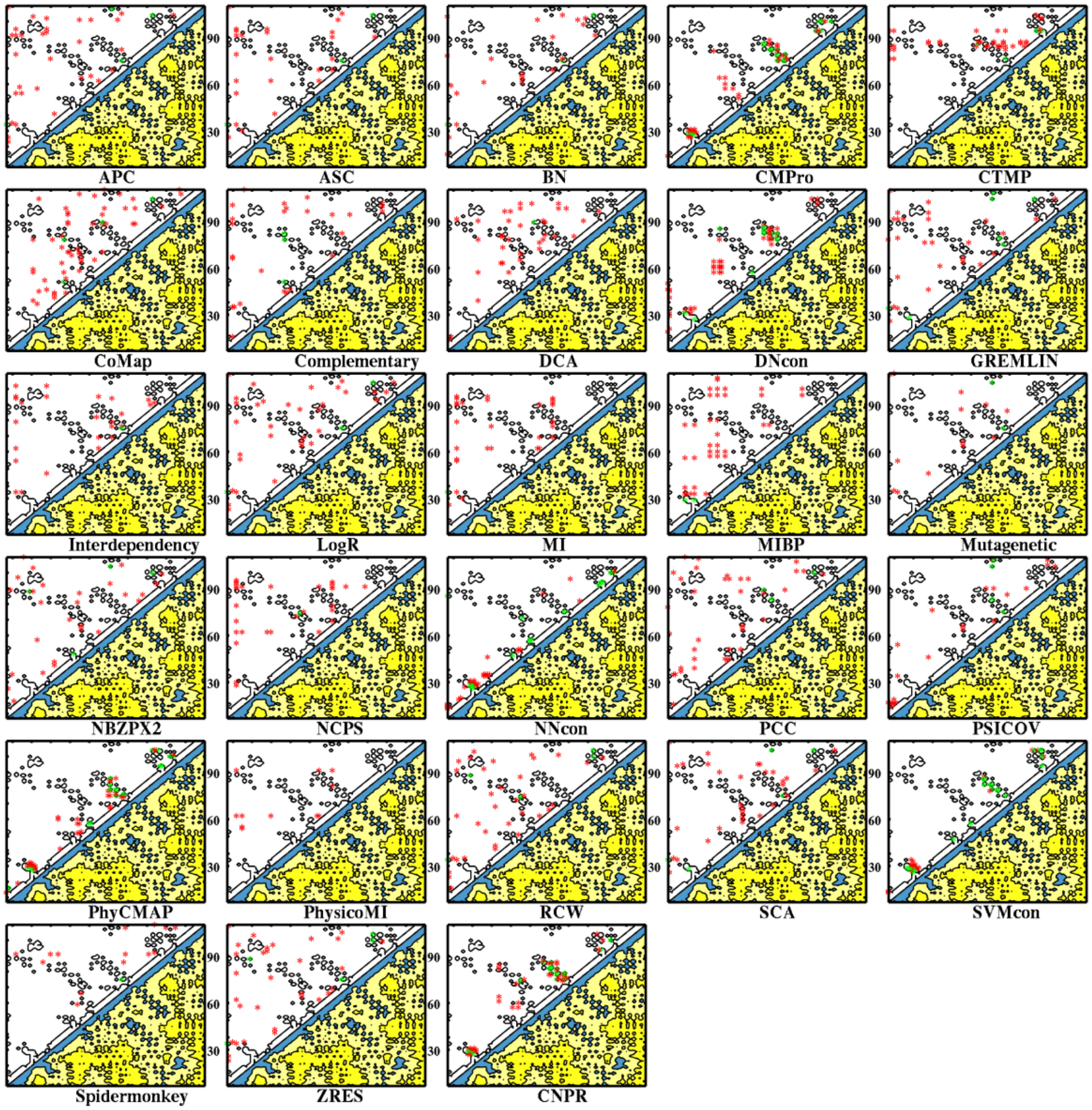

Figure S 5: Contact map of HIV-1 matrix and coevolving pairs predicted by 28 sequence-based methods. For each subplot, the protein contact map is shown at the bottom right and the top-ranked coevolving residues (L=99) predicted by sequence-based methods are shown as asterisk in the upper left side. True positive coevolving pairs are those falling within the contours of the protein contact map, and indicated as green asterisk. The others are red.

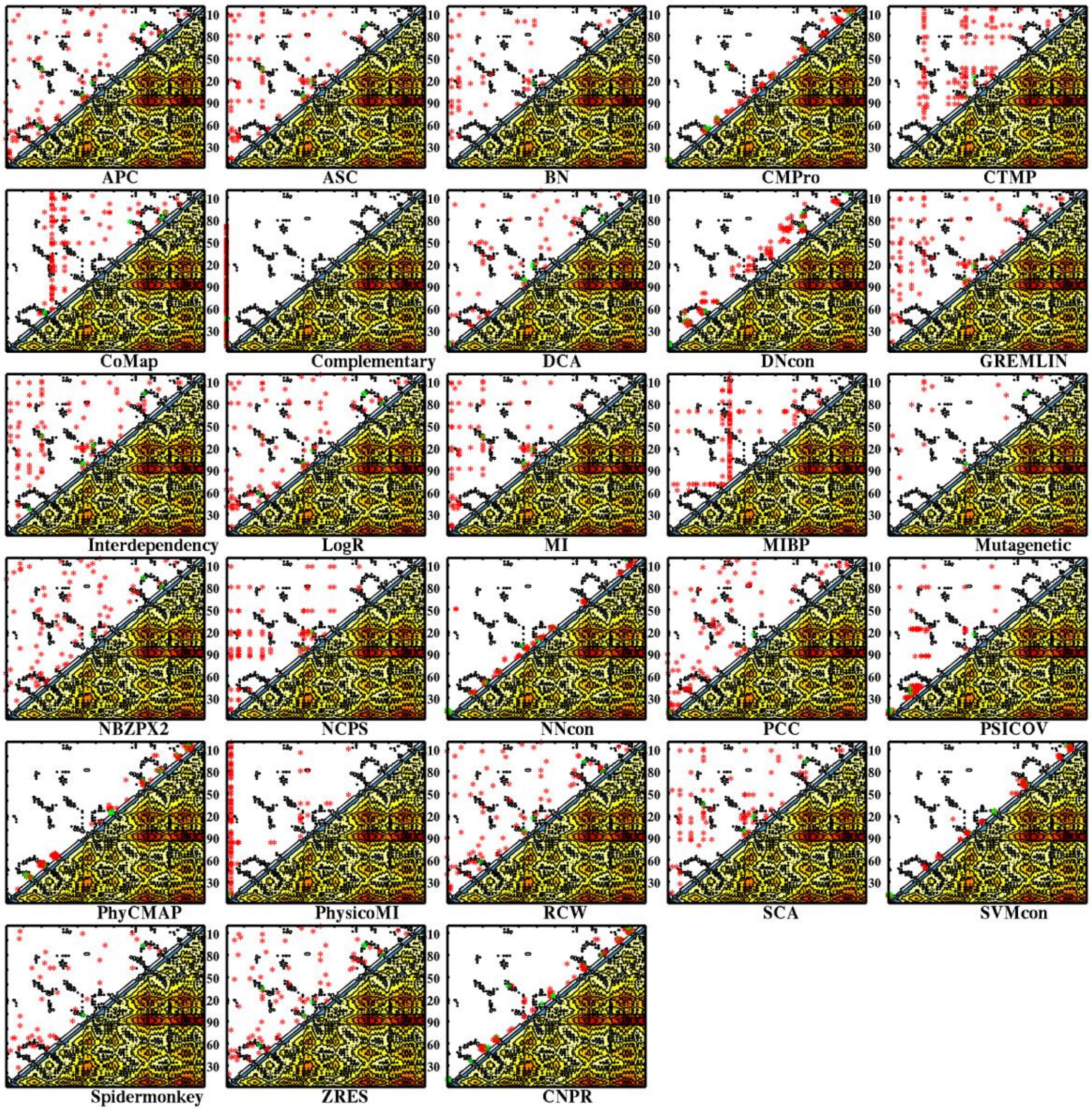

Figure S 6: Contact map of HIV-1 capsid and coevolving pairs predicted by 28 sequence-based methods. For each subplot, the protein contact map is shown at the bottom right and the top-ranked coevolving residues (L=231) predicted by sequence-based methods are shown as asterisk in the upper left side. True positive coevolving pairs are those falling within the contours of the protein contact map, and indicated as green asterisk. The others are red.

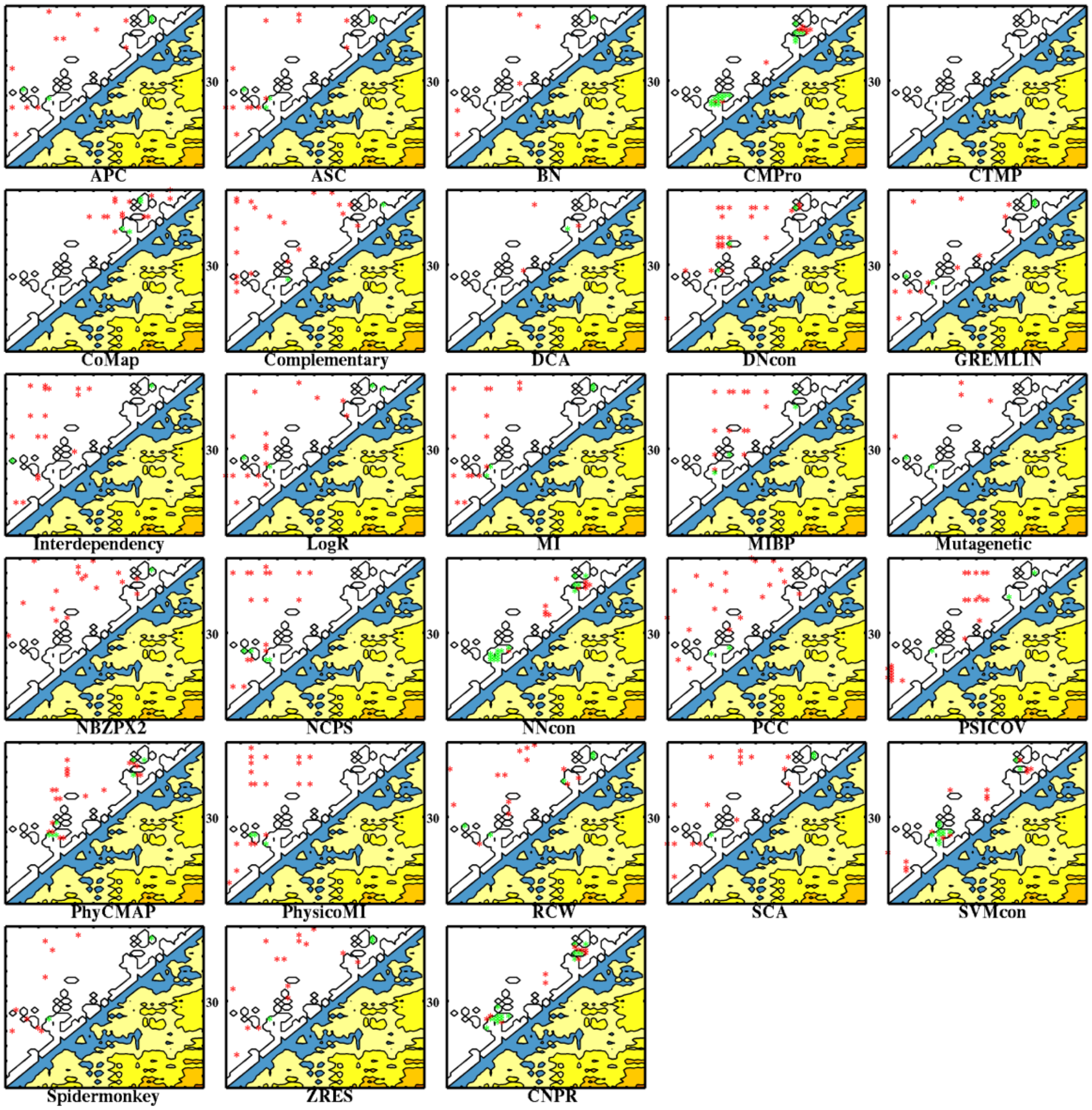

Figure S 7: HIV-1 nucleocapsid contact map and coevolving pairs predicted by 28 sequence-based methods. For each subplot, the protein contact map is shown at the bottom right and the top-ranked coevolving residues (L=55) predicted by sequence-based methods are shown as asterisk in the upper left side. True positive coevolving pairs are those falling within the contours of the protein contact map, and indicated as green asterisk. The others are red.

2. Supplementary tables

Table S 1: Summary of 7 sequence datasets for detecting HIV-1 coevolution.

HIV-1 protein	MA	CA	NC	p6	PR	p6-PR	CSM-PR
Number of sequences	605	656	768	1030	1762	788	292
Percentage of gaps	0.21%	0.01%	0.05%	0.09%	0.02%	0.02%	0.22%
Amino acid diversity	12.46%	4.57%	9.37%	14.30%	7.59%	11.95%	9.77%

Table S 2: Summary of PI-associated Gag and protease substitutions reported in experimental or clinical studies.

Gag substitutions #	Protease substitutions	Ref
L449F+P453T	L10F+G16E+K20T+A28S+M46I+A71V	[1]
R452K	Q58E+A71V	[1]
L449F	L10F	[2]
L449F	L10F+I84V	[2]
L449F	L10F+M46I+I50V	[2]
L449F	L10F+M46I+I47V+I50V	[2]
R452S	L10F+K20I+M36I+M46I+I54V+L63P+A71V+G73S+I84A	[3]
L449F	L10F+M46I+L63P+A71V+I84A	[2, 3]
A431V+L449Q	L10V+M46I+L63A/P+A71V+I84A	[3]
A431V	L10I+M46I+L63P+A71V+L76V+I84A	[3]
A431V+S451I	L10F+L19I+M46I+I47V+I54V+L63P+A71V+I84A	[3]
A431V	L10V+K20I+M36I+M46I+A71V+G73S+L76V+I84A	[3]
L449F	L10I+M46I+L63P+A71V+V77I+I84A	[3]
A431V	L10I+M46I+L63H+A71V+V77I+I84A	[3]
A431V+L449V+R452K	L10F+K20I+M46I+I54M+L63P+A71V+G73T+V77I+I84A	[3]
A431V+L449F	L10F+M46I+L63P+A71V+V77I+I84A	[3]
A431V+L449Q+S451T	L10I+L33F+M46I+I54V+L63P+A71V+L76V+I84A	[3]
R452S	L10F+K20I+M36I+M46I+I54V+L63P+A71V+G73S+I84A	[3]
S451N	K20I+M36I+V82I+I84C	[3]
L449F	L10I+L19I+L24I+L63H+I84C	[3]
A431V	L10I+G16A+M46I+L63P+L76V+I84C	[3]
A431V+L449F	L10F+L19V+L24I+M46L+L63P+I84C	[3]
A431V+L449F	L10I+L24I+M46L+L63P+A71T+G73S+V77I+I84C	[3]
A431V	M46I+L76V	[4]
A431V	M46I	[4]
A431V	L76V	[4]
P453L	D30N+N88D	[5]
E12K+L75R+H219Q+V390D+R409K	L10F+D30N+K45I+A71V	[6]
E12K+L75R+H219Q+V390D+R409K	D30N+M46I+V77I	[6]
L75R+H219Q+V390D	L10F+V32I+M46I+I84V	[7]
A431V	L10F+K20T+L33F+M36I+M46I+I54V+L63P	[8]
A431V	L10I+K20R+L33F+M46L+I54L+L63P+A71V+G73S+V82A+L90M	[8]
I437V	L10I+G48V+I54V+L63P+V77I+V82A	[8]
L483P+K490R	L10I+I15V+E34Q+M36I+T37N+I54A+Q58E+V82A	[9]
I376V+ L483P+K490R	L10I+I15V+E34Q+M36I+T37N+I54A+Q58E+V82A	[9]
I376V+E398V+L483P+K490R	L10I+I15V+E34Q+M36I+T37N+I54A+Q58E+V82A	[9]
L449P+P453L	L19Ins+E21D+A22V+M46I/L63P+A71V+I84V+I93L	[10]
L449P+P453L	M46I+L63P+A71V+I84V+I93L	[10]
L449F	I50V	[11]
L449F	M46I+I50V	[11]
P453L	I50V	[11]

Gag substitutions #	Protease substitutions	Ref
P453L	M46I+I50V	[11]
A431V	L90M	[12]
A431V	N88D+L90M	[12]
A431V	N88D	[12]
A431V	D30N+N88D+L90M	[12]
A431V	D30N+N88D	[12]
A431V	I84V	[12]
A431V	V82A	[12]
A431V	I50L	[12]
A431V	I84V+L90M	[12]
A431V	V82A+L90M	[12]
K436R	L90M	[12]
K436R	I84V+L90M	[12]
K436R	I84V	[12]
K436R	I50V	[12]
I437V	D30N+N88D	[12]
I437V	I50V	[12]
I437V	I84V+L90M	[12]
I437V	V82A	[12]
I437V	V82A+L90M	[12]
I437V	I84V	[12]
L449F	D30N+N88D	[12]
L449F	V82A	[12]
L449F	I50V	[12]
L449F	V82A+L90M	[12]
L449F	L90M	[12]
L449F	N88D	[12]
R452S	I84V+L90M	[12]
R452S	I84V	[12]
R452S	L90M	[12]
P453L	I84V	[12]
P453L	I84V+L90M	[12]
P453L	L90M	[12]
P453L	V82A	[12]
P453L	V82A+L90M	[12]
A431V	M46I +L76V	[13]
P453L	I84V	[14]
A431V	M46I/L,V82A/F/T	[14]
I437A	V82A	[15]
I437V	G48V,I50V,I54A/V,V82A/T	[15]
P459Ins	V82A/F/T/S	[16]
S451N	L10I	[17]
I437T/V	L76V	[18]
A431V	M46I	[19]
N382A	I15V	[20]
A431V	M46L/I+I54V+ V82A	[21]
L449P,S451N,P453L	D30N+N88D	[22]
A431V	L24I+M46I/L+I54V+V82A	[23]
I437V	I54V+V82F/T/S	[23]
L449V	I54M/L/S/T/A	[23]

Gag substitutions #	Protease substitutions	Ref
L449F+R452S+P453L	D30N+I84V	[23]
P453L	V82A	[23]
L449F,S451N/T	D30N+N88D	[24]
A431V	M46I/L,I54V,V82A/T/F	[25]
S373Q,L449P	K20I/R/M,L89M/I	[25]
S125K+Y132F+G62R+I437V	L10I+A71V+N88S	[26]
P453L	N88D	[27]

#: Substitution: the symbol "+" indicates multiple amino acid substitutions observed simultaneously (e.g. Y79F+T81A indicates the presence of both Y79F and T81A).

Table S 3: Ranking of sequence-based methods using individual HIV-1 datasets

Sequence-based method	Area-under-curve (AUC)							Accuracy(1)		Harmonic distance(2)		Euclidean distance(3)	
	MA	CA	NC	p6	PR	PR-p6	PR-GCS	L/2	L	L/2	L	L/2	L
APC	11.5*	12.5	14.5	12	14.5	13.5	11	16	15	11.5	12.5	13	14
ASC	15	20	14.5	10	11	12	14	11	8	7	10	7	15
BN	4.5	12.5	10	13	4	18.5	0	24	22	26.5	23	27	24
CMPro	2.5	5.5	1	9	5	10	7	2	3	1	3	1	2
CoMap	25.5	23.5	11	0	19.5	0	22.5	27	27	14.5	9	9	8
Complementary	25.5	23.5	19.5	19	22.5	20	19	26	25	26.5	26	23	23
CTMP	21	23.5	0	0	14.5	9	0	28	28	28	24.5	11	7
DCA	18.5	12.5	14.5	8	26	11	10	17	17	13	17	14	12
DNcon	28	26.5	8	0	9	0	5.5	8	10	6	6	6	6
GREMLIN	15	16.5	12	6	10	16	13	12	14	11.5	15.5	12	16
Interdependency	7	7.5	6	0	7	0	0	19	18	14.5	12.5	18	13
LogR	18.5	16.5	24.5	7	19.5	17	19	15	16	18	18.5	19	21
MI	27	16.5	17.5	2	12.5	4	3	6	7	9.5	12.5	15	18
MIBP	11.5	28	19.5	14.5	22.5	13.5	8	25	22	19	15.5	16	10.5
Mutagenetic	23.5	5.5	5	0	8	2.5	16	9	6	16.5	12.5	24	20
NBZPX2	15	23.5	24.5	17.5	21	22	22.5	23	22	23.5	24.5	26	25.5
NCPS	9.5	26.5	24.5	4	17	2.5	2	7	9	20.5	20.5	25	27
NNcon	6	2	3	0	1	0	0	3	2	3	1.5	3	3
PCC	23.5	9.5	22	0	26	18.5	15	21	24	23.5	27	20.5	25.5
PhyCMAP	1	3.5	4	16	6	5.5	19	5	5	5	5	5	4
PhysicoMI	8	9.5	27	2	28	8	12	20	26	25	28	28	28
PSICOV	15	7.5	24.5	17.5	26	22	21	18	19	22	20.5	20.5	17
RCW	21	20	17.5	5	17	5.5	4	13	11	9.5	7.5	10	10.5
SCA	21	16.5	21	20	12.5	7	5.5	10	12.5	16.5	18.5	17	19
Spidermonkey	9.5	12.5	9	14.5	24	22	17	22	20	20.5	22	22	22
SVMcon	4.5	1	7	0	2.5	0	0	4	4	4	4	4	5
ZRES	15	20	14.5	11	17	15	9	14	12.5	8	7.5	8	9
CNPR	2.5	3.5	2	2	2.5	1	1	1	1	2	1.5	2	1

(1): Rankings are obtained by the average accuracy of individual methods (Table S4)
(2): Rankings are obtained by the average Harmonic distance of individual methods (Table S5)
(3): Rankings are obtained by the average Euclidean distance of individual methods (Table S6).

*: the average ranking of the method following the calculation procedures in [28]. We give a simple example to explain the calculation of this average ranking:

	AUC		Ranking		AUC		Ranking
Method 1:	0.9	=>	1	Method 1:	0.8	=>	(1+2) = 1.5
Method 2:	0.8	=>	2	Method 2:	0.8	=>	(1+2) = 1.5
Method 3:	0.7	=>	3	Method 3:	0.7	=>	3

In the example on right, the average ranking of the first and second methods is calculated as (1+2)/2 = 1.5. For the method 3, it remains the same ranking as 3.

Table S 4: Accuracy of sequence-based methods on individual HIV-1 datasets (threshold of contact distance: 8 angstroms)

Sequence-based method	Accuracy of the L/2 top-ranked long-range couplings								Accuracy of the L top-ranked long-range couplings							
	MA	CA	NC	p6	PR	PR-p6	PR-GCS	Average	MA	CA	NC	p6	PR	PR-p6	PR-GCS	Average
APC	9%	6.9%	17.9%	0%#	22%	9.2%	10.7%	10.8%	9%	4.3%	19.6%	0%	14%	4.6%	8.7%	8.6%
ASC	7.5%	6.9%	21.4%	0%	20%	17.1%	32%	15%	6.8%	4.7%	17.9%	1.9%	19%	11.2%	20.7%	11.7%
BN	6%	2.5%	7.1%	0%	16%	6.6%	2.9%	5.9%	5.6%	2.5%	6.5%	1.9%	11%	6.4%	2.9%	5.2%
CMPro	41.8%	26.7%	64.3%	0%	64%	0%	5.3%	28.9%	30.8%	17.7%	50%	1.9%	49%	0%	8%	22.5%
CoMap	7.5%	5.2%	10.7%	0%	0%	0%	4%	3.9%	6.8%	3.9%	8.9%	0%	4%	0%	6.7%	4.3%
Complementary	4.5%	5.2%	7.1%	0%	6%	5.3%	0%	4%	6%	4.3%	3.6%	0%	6%	5.3%	8%	4.7%
CTMP	9.3%	2.7%	0%	0%	0%	11.1%	0%	3.3%	9.3%	2.7%	0%	0%	0%	11.1%	0%	3.3%
DCA	3%	9.5%	17.9%	3.7%	4%	5.3%	21.3%	9.2%	6%	6%	14.3%	1.9%	4%	3.9%	13.3%	7.1%
DNcon	20.9%	18.1%	10.7%	0%	62%	0%	4%	16.5%	17.3%	14.2%	8.9%	0%	35%	0%	3.7%	11.3%
GREMLIN	10.4%	5.2%	17.9%	0%	26%	13.2%	24%	13.8%	6.8%	3%	12.5%	0%	17%	9.2%	18%	9.5%
Interdependency	4.5%	7.7%	11.1%	4.2%	20%	0%	3.3%	7.3%	5.7%	7.7%	11.1%	4.2%	17%	0%	3.3%	7%
LogR	6%	7.8%	17.9%	0%	18%	9.2%	21.3%	11.4%	6%	5.2%	14.3%	0%	15%	5.3%	12.7%	8.3%
MI	7.5%	5.2%	17.9%	7.4%	24%	23.7%	40%	17.9%	4.5%	3.9%	16.1%	3.8%	16%	18.4%	25.3%	12.6%
MIBP	6%	0%	17.9%	0%	8%	0%	0%	4.5%	5.3%	1.7%	17.9%	0%	12%	0%	0%	5.3%
Mutagenetic	10%	9.1%	20%	0%	22.2%	14.3%	35.7%	15.9%	10%	9.1%	20%	0%	22%	14.3%	35.7%	15.9%
NBZPX2	10.4%	3.4%	7.1%	0%	14%	2.6%	5.3%	6.1%	6.8%	3%	8.9%	0%	10%	2%	6%	5.2%
NCPS	0%	3.4%	17.9%	7.4%	12%	31.6%	46.7%	17%	0.8%	2.2%	10.7%	3.8%	14%	21.1%	28.7%	11.6%
NNcon	37.2%	22.4%	67.9%	0%	70%	2.6%	0%	28.6%	37.2%	20%	44.6%	0%	62%	2.6%	0%	23.8%
PCC	7.5%	3.4%	7.1%	0%	6%	7.9%	17.3%	7%	4.5%	4.3%	3.6%	0%	9%	3.9%	0.1%	5%
PhyCMAP	34.3%	23.3%	28.6%	0%	44%	2.6%	2.7%	19.4%	33.8%	15.9%	30.4%	0%	33%	4.6%	2.7%	17.2%
PhysicoMI	3%	0.9%	10.7%	7.4%	12%	5.3%	10.7%	7.1%	1.5%	0.4%	7.1%	3.8%	7%	5.3%	7.3%	4.6%
PSICOV	10.4%	13.8%	10.7%	0%	4%	11.8%	8%	8.4%	7.5%	6.9%	5.4%	0%	9%	7.9%	6.7%	6.2%
RCW	9%	6.9%	21.4%	0%	14%	13.2%	21.3%	12.3%	8.3%	4.3%	19.6%	1.9%	14%	10.5%	18%	10.9%
SCA	7.5%	5.2%	10.7	0%	20%	28.9%	37.3%	15.7%	6%	3.9%	08.9%	0%	14%	17.8%	24.7%	10.8%
Spidermonkey	4.5%	4.3%	14.3	0%	8%	2.6%	12%	6.5%	3.8%	3.4%	10.7%	0%	9%	2.6%	0.1%	5.7%
SVMcon	47.8%	19.8%	46.4	0%	58%	0%	0%	24.6%	38.5%	18.8%	32.1%	0%	39%	0%	0%	18.3%
ZRES	10.4%	6.9%	17.9	3.7%	16%	7.9%	21.3%	12%	8.3%	4.3%	19.6%	3.8%	13%	7.9%	18%	10.7%
CNPR	38.8% (2)	25% (2)	57.1% (3)	7.4% (2.5)	56% (3)	19.7% (4)	38.7% (3)	34.7% (1)	30.3% (5)	21.2% (1)	42.9% (3)	3.8% (4)	44% (3)	17.9% (3)	28.9% (2)	26.9% (1)

Table S 5: Harmonic distance of sequence-based methods using individual HIV-1 datasets

Sequence-based method	Harmonic distance of the L/2 top-ranked long-range couplings						Harmonic distance of the L top-ranked long-range couplings					
	MA	CA	NC	p6	PR	Average	MA	CA	NC	p6	PR	Average
APC	0.032	0.037	0.043	0.012	0.073	0.039	0.018	0.024	0.034	0.017	0.04	0.027
ASC	0.025	0.029	0.066	0.057	0.078	0.051	0.015	0.02	0.024	0.022	0.058	0.028
BN	0.014	0.004	0	-0.013	0.04	0.009	0.01	0.004	-0.01	0.002	0.035	0.008
CMPro	0.177	0.161	0.222	0.07	0.198	0.166	0.139	0.113	0.175	0.064	0.16	0.13
CoMap	0.033	0.023	0.032	0.078	-0.022	0.029	0.032	0.017	0.027	0.078	-0.007	0.029
Complementary	-0.003	0.034	-0.013	0.005	0.016	0.008	0.003	0.023	-0.025	0.001	0.01	0.003
CTMP	0.045	-0.003	0	-0.038	0.017	0.004	0.045	-0.003	0	-0.038	0.017	0.004
DCA	0.027	0.048	0.047	0.028	-0.003	0.03	0.023	0.037	0.045	0.007	0.002	0.023
DNcon	0.119	0.103	0.059	0.003	0.182	0.093	0.104	0.086	0.054	-0.013	0.119	0.07
GREMLIN	0.034	0.024	0.041	0.015	0.087	0.04	0.019	0.005	0.023	0.009	0.061	0.024
Interdependency	0.016	0.028	-0.022	0.046	0.071	0.028	0.016	0.028	-0.022	0.046	0.062	0.026
LogR	0.012	0.037	0.029	-0.01	0.053	0.024	0.006	0.024	0.016	-0.007	0.037	0.015
MI	0.017	0.017	0.023	0.078	0.08	0.043	-0.002	0.009	0.018	0.045	0.06	0.026
MIBP	0.048	0.009	0.049	-0.016	0.017	0.021	0.041	0.013	0.057	-0.029	0.035	0.023
Mutagenetic	0.029	0.016	0.034	-0.034	0.09	0.027	0.029	0.016	0.034	-0.034	0.09	0.027
NBZPX2	0.031	0.018	-0.004	-0.011	0.023	0.011	0.009	0.017	-0.003	-0.011	0.011	0.005
NCPS	-0.013	-0.005	0.006	0.066	0.037	0.018	-0.018	-0.01	-0.007	0.038	0.053	0.011
NNcon	0.134	0.135	0.197	0.064	0.209	0.148	0.134	0.116	0.146	0.064	0.2	0.132
PCC	0.024	0.027	-0.003	0.008	0.008	0.013	0.001	0.02	-0.023	-0.01	0.011	0
PhyCMAP	0.155	0.159	0.121	0.025	0.131	0.118	0.157	0.135	0.118	0.018	0.108	0.107
PhysicoMI	-0.002	-0.041	-0.016	0.051	0.052	0.009	-0.014	-0.029	-0.01	0.031	0.018	-0.001
PSICOV	0.051	0.063	-0.022	-0.007	-0.003	0.016	0.032	0.036	-0.019	0	0.013	0.012
RCW	0.03	0.042	0.048	0.057	0.042	0.044	0.02	0.024	0.04	0.03	0.043	0.032
SCA	0.032	0.018	0.015	-0.008	0.079	0.027	0.015	0.007	0.004	0.001	0.052	0.016
Spidermonkey	0.009	0.012	0.016	0.011	0.041	0.018	0.001	0.013	0.003	0.004	0.031	0.01
SVMcon	0.192	0.128	0.152	0.037	0.189	0.14	0.165	0.124	0.113	0.027	0.128	0.111
ZRES	0.034	0.044	0.053	0.061	0.041	0.046	0.022	0.024	0.037	0.044	0.035	0.032
CNPR	0.175 (2)	0.155 (3)	0.19 (3)	0.08 (1)	0.178 (5)	0.155 (2)	0.144 (3)	0.137 (1)	0.16 (2)	0.068 (1)	0.152 (2)	0.132 (1.5)

Table S 6: Average Euclidean distance of the top-ranked long-range couplings predicted by sequence-based methods

Sequence-based method	Average Euclidean distance of the L/2 top-ranked long-range couplings (Å)						Average Euclidean distance of the L top-ranked long-range couplings (Å)					
	MA	CA	NC	p6	PR	Average	MA	CA	NC	p6	PR	Average
APC	16.45	21.64	14.74	20.81	13.25	17.38	17.72	22.9	16.69	20.65	15.06	18.6
ASC	16.69	22.84	13.53	16.67	12.31	16.41	17.73	23.54	17.63	20.54	14.01	18.69
BN	18.27	25.53	17.65	23.8	14.46	19.94	18.92	25.53	19.09	22.34	14.76	20.13
CMPro	9.01	11.19	7.47	14.2	8.38	10.05	10.51	14.7	8.8	15.33	9.49	11.77
CoMap	15.63	21.88	14.56	14.31	17.87	16.85	15.95	22.87	15.14	14.31	17.46	17.14
Complementary	19.27	20.32	19.32	20.95	15.52	19.08	18.82	22.6	19.91	22.35	16.37	20.01

CTMP	15.36	27.88	-	27.72	13.91	16.98	15.36	27.88	-	27.72	13.91	16.98
DCA	15.41	20.27	14.53	19.76	17.18	17.43	16.52	21.6	15.18	21.96	17	18.45
DNcon	11.18	15.69	12.55	19.74	9.14	13.66	11.6	16.62	13.87	22.35	11.12	15.11
GREMLIN	16.58	22.21	14.8	19.7	12.39	17.14	17.78	24.97	16.2	21.52	13.37	18.77
Interdependency	17.87	23.22	20.34	17.54	13.02	18.4	18.41	23.22	20.34	17.54	13.38	18.58
LogR	17.68	21.95	15.72	22.64	14.2	18.44	18.24	22.92	16.99	23.08	15.36	19.32
MI	17.66	24.05	16.98	16.73	12.59	17.6	19.17	25.57	17.81	18.79	13.45	18.96
MIBP	14.06	21.77	13.89	23.39	15.9	17.8	14.52	21.93	13.49	25.55	15.13	18.12
Mutagenetic	17.43	23.12	15.53	27.77	11.82	19.13	17.43	23.12	15.53	27.77	11.82	19.13
NBZPX2	16.52	23.51	17.53	23.77	16.23	19.51	18.22	23.68	18.31	24.03	16.76	20.2
NCPS	19.91	26.33	19.29	16.98	14.34	19.37	20.6	27.69	19.87	19.63	13.57	20.27
NNcon	10.87	14.15	8.35	15.26	7.6	11.25	10.87	15.52	10.46	15.26	7.93	12.01
PCC	17.02	21.43	17.38	21.22	16.09	18.63	18.71	22.86	19.26	23.57	16.59	20.2
PhyCMAP	9.58	11.17	10.11	17.6	10.7	11.83	9.55	12.21	10.39	19.01	11.6	12.55
PhysicoMI	18.76	32.18	19.4	18.33	13.65	20.46	20.03	30.06	19.03	20.09	16.08	21.06
PSICOV	14.74	19.37	19.22	22.49	17.33	18.63	16.18	20.36	18.15	22.48	16.77	18.79
RCW	16.71	21.14	15.21	16.67	14.67	16.88	17.36	22.56	15.93	20.07	14.66	18.12
SCA	16.36	24.03	16.07	22.61	12.22	18.26	17.78	24.77	17.39	21.86	13.6	19.08
Spidermonkey	17.81	24.4	16.88	20.56	14.83	18.89	19.06	23.98	18.15	22.24	15.41	19.77
SVMcon	8.52	13.3	9.54	16.82	8.89	11.42	9.48	13.43	11.02	17.96	11.38	12.65
ZRES	16.4	20.75	14.35	16.81	14.94	16.65	17.23	22.69	16.32	18.93	15.24	18.08
CNPR	9.0 (1)	11.23 (3)	7.77 (2)	13.67 (1)	8.65 (3)	10.06 (2)	10.06 (2)	12.78 (2)	9.41 (2)	15 (2)	9.77 (3)	11.4 (1)

Table S 7: Summary of long-range residue contacts derived from HIV-1 Gag and protease protein structures

Protein	Number of intra-domain long-range contact (1)				Number of inter-domain long-range contact (2)				Percentage(3)
	helix-helix	strand-strand	helix-strand	others(4)	helix-helix	strand-strand	helix-strand	others	
Matrix	346	0	0	277	0	0	4	23	650/5995=10.46%
Capsid (5)	520	15	22	680	46	0	19	8	1310/22993=5.59%
Nucleocapsid	4	0	0	192	0	0	0	0	196/1225=14.78%
P6	7	0	0	54	0	0	0	0	61/1081=5.19%
Protease	3	233	42	248	0	0	13	8	547/4371=12.00%
Total	880	248	64	1451	46	0	36	39	2764/36712=7.53%

Long-range residues are defined as two residues have at least 6 amino acids apart in the protein sequence.Two residues are in contacts if the Euclidean distance of their Cα atoms is less than 8 Å in the protein 3D structure.

(1) Intra-domain long-range contacts: the number of long-range contacts of residue pairs (see definitions in Methods) within a protein domain, which are classified according to the type of secondary structures involved (e.g. a helix-strand contact indicates contact between a protein residue in an alpha-helix structure and a protein residue in a beta strand structure).

(2) Inter-domain contacts: residue contacts between different protein domains.

(3) Percentage: the proportion of long-range residue pairs in contact, calculated using PDB data (e.g. for the matrix protein, 650/5995=10.46% indicates that 5995 possible

long-range pairs of positions are resolved in the crystal structure and 650 of them are in direct contact).
(4) Others: all the other residue contacts (helix-to-coil, strand-to-coil and coil-to-coil contacts)
(5) Capsid contact map is based on crystalized hexamer.
PDB code: 1HIW (matrix), 3H4E (capsid), 1A1T (nucleocapsid), 2C55 (p6), 1TW7 (protease).

Table S 8: Accuracy of sequence-based methods on individual HIV-1 datasets (threshold of contact distance: 5 angstroms)

Sequence-based method	Accuracy of the L/2 top-ranked long-range couplings								Accuracy of the L top-ranked long-range couplings							
	MA	CA	NC	p6	PR	PR-p6	PR-GCS	Average	MA	CA	NC	p6	PR	PR-p6	PR-GCS	Average
APC	4.5%	1.7%	3.6%	0%	2%	9.2%	10.7%	4.5%	3%	1.3%	3.6%	0%	1%	4.6%	8.7%	3.2%
ASC	4.5%	0.9%	7.1%	0%	2%	17.1%	32%	9.1%	3%	1.3%	3.6%	0%	1%	11.2%	20.7%	5.8%
BN	3%	0%	3.6%	0%	2%	6.6%	2.9%	2.6%	2.8%	0%%	3.2%	0%	1.2%	6.4%	2.9%	2.4%
CMPro	9%	3.4%	10.7%	0%	6%	0%	5.3%	4.9%	5.3%	2.6%	12.5%	0%	6%	0%%	8%	4.9%
CoMap	1.9%	0%	0%	0%	0%	11.1%	0%	1.9%	1.9%	0%	0%%	0%	0%	11.1%	0%%	1.9%
Complementary	1.5%	0%	0%	0%	0%	0%	4%	0.8%	1.5%	0.4%	1.8%	0%	1%	0%	6.7%	1.6%
CTMP	0%	0%	0%	0%	0%	5.3%	0%	0.8%	1.5%	0.4%	0%	0%	0%	5.3%	8%	2.2%
DCA	1.5%	1.7%	3.6%	0%	0%	5.3%	21.3%	4.8%	2.3%	0.9%	3.6%	0%	0%	3.9%	13.3%	3.4%
DNcon	3%	2.6%	3.6%	0%	8%	0%	4%	3%	3%	1.3%	3.6%	0%	6%	0%	3.8%	2.5%
GREMLIN	3%	0.9%	3.6%	0%	2%	13.2%	24%	6.7%	2.3%	0.4%	3.6%	0%	2%	9.2%	18%	5.1%
Interdependency	0%	1.1%	0%	0%	0%	0%	3.3%	0.6%	1.1%	1.1%	0%	0%	0%	0%	3.3%	0.8%
LogR	3%	1.7%	3.6%	0%	2%	9.2%	21.3%	5.8%	2.3%	1.3%	3.6%	0%	1%	5.3%	12.7%	3.7%
MI	3%	0.9%	7.1%	0%	2%	23.7%	40%	11%	2.3%	0.9%	3.6%	0%	1%	18.4%	25.3%	7.3%
MIBP	1.5%	0%	0%%	0%	0%	0%	0%	0.2%	0.8%	0%%	3.6%	0%	1%	0%	0%	0.8%
Mutagenetic	3.3%	0%	13.3	0%	0%	14.3%	35.7%	9.5%	3.3%	0%	13.3%	0%	0%	14.3%	35.7%	9.5%
NBZPX2	3%	0%	0%	0%	0%	2.6%	5.3%	1.6%	2.3%	1.3%	0%	0%	1%	2%	6%	1.8%
NCPS	0%	0.9%	3.6%	0%	0%	31.6%	46.7%	11.8%	0%	0.4%	1.8%	0%	2%	21.1%	28.7%	7.7%
NNcon	7%	2.6%	7.1%	0%	6%	2.6%	0%	3.6%	7%	3.3%	8.9%	0%	8%	2.6%	0%	4.3%
PCC	3%	0.9%	0%	0%	0%	7.9%	17.3%	4.2%	1.5%	0.4%	0%	0%	0%	3.9%	10%	2.3%
PhyCMAP	1.5%	0.9%	3.6%	0%	0%	11.8%	8%	3.7%	1.5%	0.4%	1.8%	0%	1%	7.9%	6.7%	2.8%
PhysicoMI	7.5%	3.4%	7.1%	0%	4%	2.6%	2.7%	3.9%	6.8%	1.7%	5.4%	0%	3%	4.6%	2.7%	3.4%
PSICOV	1.5%	0%	3.6%	0%	0%	5.3%	10.7%	3%	0.8%	0%	1.8%	0%	0%	5.3%	7.3%	2.2%
RCW	4.5%	2.6%	3.6%	0%	0%	13.2%	21.3%	6.4%	3%	1.3	5.4%	0%	2%	10.5%	18%	5.7%
SCA	3%	0.9%	3.6%	0%	4%	28.9%	37.3%	11.1%	1.5%	0.4	3.6%	0%	2%	17.8%	24.7%	7.1%
Spidermonkey	13.4%	2.6%	10.7%	0%	6%	0%	0%	4.7%	9.4%	2.3	7.1%	0%	3%	0%	0%	3.1%
SVMcon	1.5%	0.9%	7.1%	0%	2%	2.6	12%	3.7%	0.8%	0.9	3.6%	0%	1%	2.6%	10%	2.7%
ZRES	6%	1.7%	3.6%	0%	0%	7.9	21.3%	5.8%	3%	1.3	5.4%	0%	1%	7.9%	18%	5.2%
CNPR	7.5% (2.5)	3.4% (2)	17.9% (1)	0%	4% (6)	19.7% (4)	38.7% (3)	13% (1)	5.3% (3)	2.6% (2.5)	10.7% (3)	0%	6% (2.5)	17.8% (3.5)	28.7% (1.5)	10.1% (1)

References

1. Yates PJ, Hazen R, St Clair M, Boone L, Tisdale M, Elston RC: **In vitro development of resistance to human immunodeficiency virus protease inhibitor GW640385.** *Antimicrob Agents Chemother* 2006, **50:**1092-1095.
2. Prado JG, Wrin T, Beauchaine J, Ruiz L, Petropoulos CJ, Frost SD, Clotet B, D'Aquila RT, Martinez-Picado J: **Amprenavir-resistant HIV-1 exhibits lopinavir cross-resistance and reduced replication capacity.** *AIDS* 2002, **16:**1009-1017.
3. Mo H, Parkin N, Stewart KD, Lu L, Dekhtyar T, Kempf DJ, Molla A: **Identification and structural characterization of I84C and I84A mutations that are associated with high-level resistance to human immunodeficiency virus protease inhibitors and impair viral replication.** *Antimicrob Agents Chemother* 2007, **51:**732-735.
4. Nijhuis M, Wensing AM, Bierman WF, de Jong D, Kagan R, Fun A, Jaspers CA, Schurink KA, van Agtmael MA, Boucher CA: **Failure of treatment with first-line lopinavir boosted with ritonavir can be explained by novel resistance pathways with protease mutation 76V.** *J Infect Dis* 2009, **200:**698-709.
5. Shibata J, Sugiura W, Ode H, Iwatani Y, Sato H, Tsang H, Matsuda M, Hasegawa N, Ren F, Tanaka H: **Within-host co-evolution of Gag P453L and protease D30N/N88D demonstrates virological advantage in a highly protease inhibitor-exposed HIV-1 case.** *Antiviral Res* 2011, **90:**33-41.
6. Aoki M, Venzon DJ, Koh Y, Aoki-Ogata H, Miyakawa T, Yoshimura K, Maeda K, Mitsuya H: **Non-cleavage site gag mutations in amprenavir-resistant human immunodeficiency virus type 1 (HIV-1) predispose HIV-1 to rapid acquisition of amprenavir resistance but delay development of resistance to other protease inhibitors.** *J Virol* 2009, **83:**3059-3068.
7. Gatanaga H, Suzuki Y, Tsang H, Yoshimura K, Kavlick MF, Nagashima K, Gorelick RJ, Mardy S, Tang C, Summers MF, Mitsuya H: **Amino acid substitutions in Gag protein at non-cleavage sites are indispensable for the development of a high multitude of HIV-1 resistance against protease inhibitors.** *J Biol Chem* 2002, **277:**5952-5961.
8. Dam E, Quercia R, Glass B, Descamps D, Launay O, Duval X, Krausslich HG, Hance AJ, Clavel F, Group AS: **Gag mutations strongly contribute to HIV-1 resistance to protease inhibitors in highly drug-experienced patients besides compensating for fitness loss.** *PLoS Pathog* 2009, **5:**e1000345.
9. Ho SK, Coman RM, Bunger JC, Rose SL, O'Brien P, Munoz I, Dunn BM, Sleasman JW, Goodenow MM: **Drug-associated changes in amino acid residues in Gag p2, p7(NC), and p6(Gag)/p6(Pol) in human immunodeficiency virus type 1 (HIV-1) display a dominant effect on replicative fitness and drug response.** *Virology* 2008, **378:**272-281.
10. Brann TW, Dewar RL, Jiang MK, Shah A, Nagashima K, Metcalf JA, Falloon J, Lane HC, Imamichi T: **Functional correlation between a novel amino acid insertion at codon 19 in the protease of human immunodeficiency virus type 1 and polymorphism in the p1/p6 Gag cleavage site in drug resistance and replication fitness.** *J Virol* 2006, **80:**6136-6145.
11. Maguire MF, Guinea R, Griffin P, Macmanus S, Elston RC, Wolfram J, Richards N, Hanlon MH, Porter DJ, Wrin T, et al: **Changes in human immunodeficiency virus type 1 Gag at positions L449 and P453 are linked to I50V protease mutants in vivo and cause reduction of sensitivity to amprenavir and improved viral fitness in vitro.** *J Virol* 2002, **76:**7398-7406.
12. Kolli M, Stawiski E, Chappey C, Schiffer CA: **Human immunodeficiency virus type 1 protease-correlated cleavage site mutations enhance inhibitor resistance.** *J Virol* 2009, **83:**11027-11042.

13. Knops E, Kemper I, Schulter E, Pfister H, Kaiser R, Verheyen J: **The evolution of protease mutation 76V is associated with protease mutation 46I and gag mutation 431V.** *AIDS* 2010, **24:**779-781.
14. Bally F, Martinez R, Peters S, Sudre P, Telenti A: **Polymorphism of HIV type 1 gag p7/p1 and p1/p6 cleavage sites: clinical significance and implications for resistance to protease inhibitors.** *AIDS Res Hum Retroviruses* 2000, **16:**1209-1213.
15. Knops E, Brakier-Gingras L, Schulter E, Pfister H, Kaiser R, Verheyen J: **Mutational patterns in the frameshift-regulating site of HIV-1 selected by protease inhibitors.** *Med Microbiol Immunol* 2012, **201:**213-218.
16. Lastere S, Dalban C, Collin G, Descamps D, Girard PM, Clavel F, Costagliola D, Brun-Vezinet F, Group NT: **Impact of insertions in the HIV-1 p6 PTAPP region on the virological response to amprenavir.** *Antivir Ther* 2004, **9:**221-227.
17. Kaufmann GR, Suzuki K, Cunningham P, Mukaide M, Kondo M, Imai M, Zaunders J, Cooper DA: **Impact of HIV type 1 protease, reverse transcriptase, cleavage site, and p6 mutations on the virological response to quadruple therapy with saquinavir, ritonavir, and two nucleoside analogs.** *AIDS Res Hum Retroviruses* 2001, **17:**487-497.
18. Lambert-Niclot S, Flandre P, Malet I, Canestri A, Soulie C, Tubiana R, Brunet C, Wirden M, Katlama C, Calvez V, Marcelin AG: **Impact of gag mutations on selection of darunavir resistance mutations in HIV-1 protease.** *J Antimicrob Chemother* 2008, **62:**905-908.
19. Cote HC, Brumme ZL, Harrigan PR: **Human immunodeficiency virus type 1 protease cleavage site mutations associated with protease inhibitor cross-resistance selected by indinavir, ritonavir, and/or saquinavir.** *J Virol* 2001, **75:**589-594.
20. Larrouy L, Charpentier C, Landman R, Capitant C, Chazallon C, Yeni P, Peytavin G, Damond F, Brun-Vezinet F, Descamps D, group As: **Dynamics of gag-pol minority viral populations in naive HIV-1-infected patients failing protease inhibitor regimen.** *AIDS* 2011, **25:**2143-2148.
21. Zhang YM, Imamichi H, Imamichi T, Lane HC, Falloon J, Vasudevachari MB, Salzman NP: **Drug resistance during indinavir therapy is caused by mutations in the protease gene and in its Gag substrate cleavage sites.** *J Virol* 1997, **71:**6662-6670.
22. Roquebert B, Malet I, Wirden M, Tubiana R, Valantin MA, Simon A, Katlama C, Peytavin G, Calvez V, Marcelin AG: **Role of HIV-1 minority populations on resistance mutational pattern evolution and susceptibility to protease inhibitors.** *AIDS* 2006, **20:**287-289.
23. Verheyen J, Litau E, Sing T, Daumer M, Balduin M, Oette M, Fatkenheuer G, Rockstroh JK, Schuldenzucker U, Hoffmann D, et al: **Compensatory mutations at the HIV cleavage sites p7/p1 and p1/p6-gag in therapy-naive and therapy-experienced patients.** *Antivir Ther* 2006, **11:**879-887.
24. Kolli M, Lastere S, Schiffer CA: **Co-evolution of nelfinavir-resistant HIV-1 protease and the p1-p6 substrate.** *Virology* 2006, **347:**405-409.
25. Malet I, Roquebert B, Dalban C, Wirden M, Amellal B, Agher R, Simon A, Katlama C, Costagliola D, Calvez V, Marcelin AG: **Association of Gag cleavage sites to protease mutations and to virological response in HIV-1 treated patients.** *J Infect* 2007, **54:**367-374.
26. Chang MW, Oliveira G, Yuan J, Okulicz JF, Levy S, Torbett BE: **Rapid deep sequencing of patient-derived HIV with ion semiconductor technology.** *J Virol Methods* 2013, **189:**232-234.
27. Rossi AH, Rocco CA, Mangano A, Sen L, Aulicino PC: **Sequence variability in p6 gag protein and gag/pol coevolution in human immunodeficiency type 1 subtype F genomes.** *AIDS Res Hum Retroviruses* 2013, **29:**1056-1060.

28. Demšar J: **Statistical comparisons of classifiers over multiple data sets.** *The Journal of Machine Learning Research* 2006, **7:**1-30.
